# Targeting METTL3 Attenuates Thyroid Inflammatory Injury by Restoring Th17/Treg Balance through a YTHDC2‐m6A‐Dependent KDR/VEGFA Loop

**DOI:** 10.1002/advs.202522453

**Published:** 2026-06-23

**Authors:** Qingyi Hu, Huan Liu, Anwen Ren, Zimei Tang, Jie Tan, Wen Yang, Jie Ming, Tao Huang

**Affiliations:** ^1^ Department of Breast and Thyroid Surgery Union Hospital Tongji Medical College Huazhong University of Science and Technology Wuhan China

**Keywords:** autoimmune thyroiditis disease, m^6^A modification, METTL3, Th17/Treg balance, VEGFA‐KDR loop

## Abstract

Autoimmune thyroiditis (AIT) is characterized by extensive lymphocytic infiltration and progressive destruction of thyroid follicular cells (TFCs), yet the molecular mechanisms underlying persistent thyroid injury remain poorly defined. Here, methyltransferase‐like 3 (METTL3) mediated N^6^‐methyladenosine (m^6^A) modification is identified as a central driver of thyroid inflammation. METTL3 expression and m^6^A levels were markedly increased in TFCs from AIT patients and EAT mice, and are positively associated with immune inflammation scores. Genetic or pharmacological inhibition of METTL3 suppressed the KDR (kinase insert domain receptor, also known as vascular endothelial growth factor receptor 2, VEGFR2)/VEGFA signaling loop, reduced inflammatory cytokine release and lymphocyte infiltration, restored Th17/Treg homeostasis, and alleviated thyroid injury. Mechanistically, ROS promoted METTL3 transcription through inhibition of SIRT1‐dependent epigenetic repression, while METTL3‐mediated m6A modification stabilized KDR mRNA in a YTHDC2‐dependent manner. In turn, activated KDR established an autocrine‐paracrine KDR/VEGFA amplification circuit that sustained inflammatory signaling. Together, these findings uncover the METTL3‐KDR axis as a critical epitranscriptomic mechanism driving chronic thyroid inflammation and nominate it as a promising therapeutic target for AIT.

## Graphical abstract

METTL3‐mediated m^6^A modification stabilizes KDR/*Kdr* mRNA through YTHDC2, amplifying the VEGFA‐KDR feedback loop and disturbing Th17/Treg balance. This pathway promotes persistent inflammation and tissue damage in patients with AIT, and inhibiting the METTL3‐KDR axis effectively disrupts this circuit, alleviating thyroid tissue injury and disease progression.

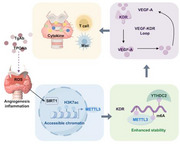

## 1 Introduction

Autoimmune thyroiditis (AIT) is a chronic organ‐specific autoimmune disease characterized by autoantibody‐mediated immune attacks on thyroid tissue [1]. The disease typically manifests as diffuse thyroid enlargement, dense lymphocytic infiltration, and elevated serum levels of thyroid peroxidase antibody (TPOAb) and/or thyroglobulin antibody (TgAb) [2]. As the disease progresses, persistent injury to thyroid follicular cells (TFCs) leads to parenchymal atrophy and eventually results in subclinical or overt hypothyroidism [3]. Moreover, patients with AIT are at increased risk of developing autoimmune polyendocrine syndromes [4] and thyroid carcinoma [5]. The activation of helper T (Th) cells, B cells, and cytotoxic T cells represents a central mechanism driving TFCs injury and death [2]. Both Th1 and Th17 cells have been shown to accelerate AIT progression, among which Th17 cells and their signature cytokine IL‐17A are recognized as key inflammatory contributors to disease initiation and progression. In contrast, regulatory T (Treg) cells exert anti‐inflammatory effects largely by restraining Th17 responses [6]. Therefore, an imbalance in the Th17/Treg axis plays a critical role in the pathogenesis of AIT, particularly during the early stage of the disease. Unfortunately, the key molecular mechanisms underlying AIT remain poorly defined, and no targeted therapies are currently available to effectively halt thyroid inflammatory injury or prevent progressive functional deterioration.

Immune dysregulation‐driven inflammatory injury is a hallmark of thyroiditis, yet the intrinsic contribution of thyroid follicular cells (TFCs) to this process remains poorly understood. Beyond its canonical role in endothelial cells, KDR (VEGFR2) has increasingly been implicated in non‐endothelial inflammatory and remodeling responses. In thyroid disorders, KDR is specifically upregulated in goiter, and the VEGFA/KDR axis in TFCs has been shown to govern TSH‐dependent follicular remodeling and goiter development [7, 8]. Given that TFCs can also produce VEGFA and that circulating VEGFA levels are markedly elevated in untreated AIT patients, these findings collectively raise the possibility that KDR‐VEGFA signaling may contribute directly to thyroid inflammation and structural remodeling in AIT.

N6‐methyladenosine (m^6^A) is the most abundant internal modification of eukaryotic mRNA and plays a key role in post‐transcriptional regulation. This modification is catalyzed by m^6^A methyltransferases (METTL3, METTL14, WTAP, RBM15/15B, VIRMA, and ZC3H13, collectively known as “writers”), removed by demethylases (FTO, ALKBH5, and ALKBH3, “erasers”), and recognized by m^6^A‐binding proteins (YTHDC1/2, YTHDF1/2/3, IGF2BP1/2/3, HNRNPs, and eIF3, “readers”) [9]. m^6^A modifications dynamically and reversibly regulate mRNA stability, splicing, localization, and translation. Accumulating evidence has shown that m^6^A ‐mediated epitranscriptomic regulation is broadly involved in inflammation [10], autoimmune diseases [11, 12], infections [13], and cancer [14]. In addition, genetic studies have suggested a link between METTL3 polymorphisms and susceptibility to AIT [15]. However, the roles of m^6^A and METTL3 in AIT remains largely unexplored.

In this study, we found that METTL3 was upregulated in TFCs from AIT patients and EAT mice. We hypothesized that METTL3 upregulation in TFCs promotes KDR‐VEGFA axis via an m^6^A‐dependent mechanism, thereby inducing IL‐17A/Th17 response and inflammatory amplification, ultimately driving AIT progression toward irreversible thyroid destruction. This work provides new mechanistic insights into AIT pathogenesis and proposes METTL3‐KDR as promising molecular targets for clinical intervention.

## 2 Results

### 2.1 METTL3 is Upregulated in TFCs and may Drive AIT Initiation and Progression

**FIGURE 1:**
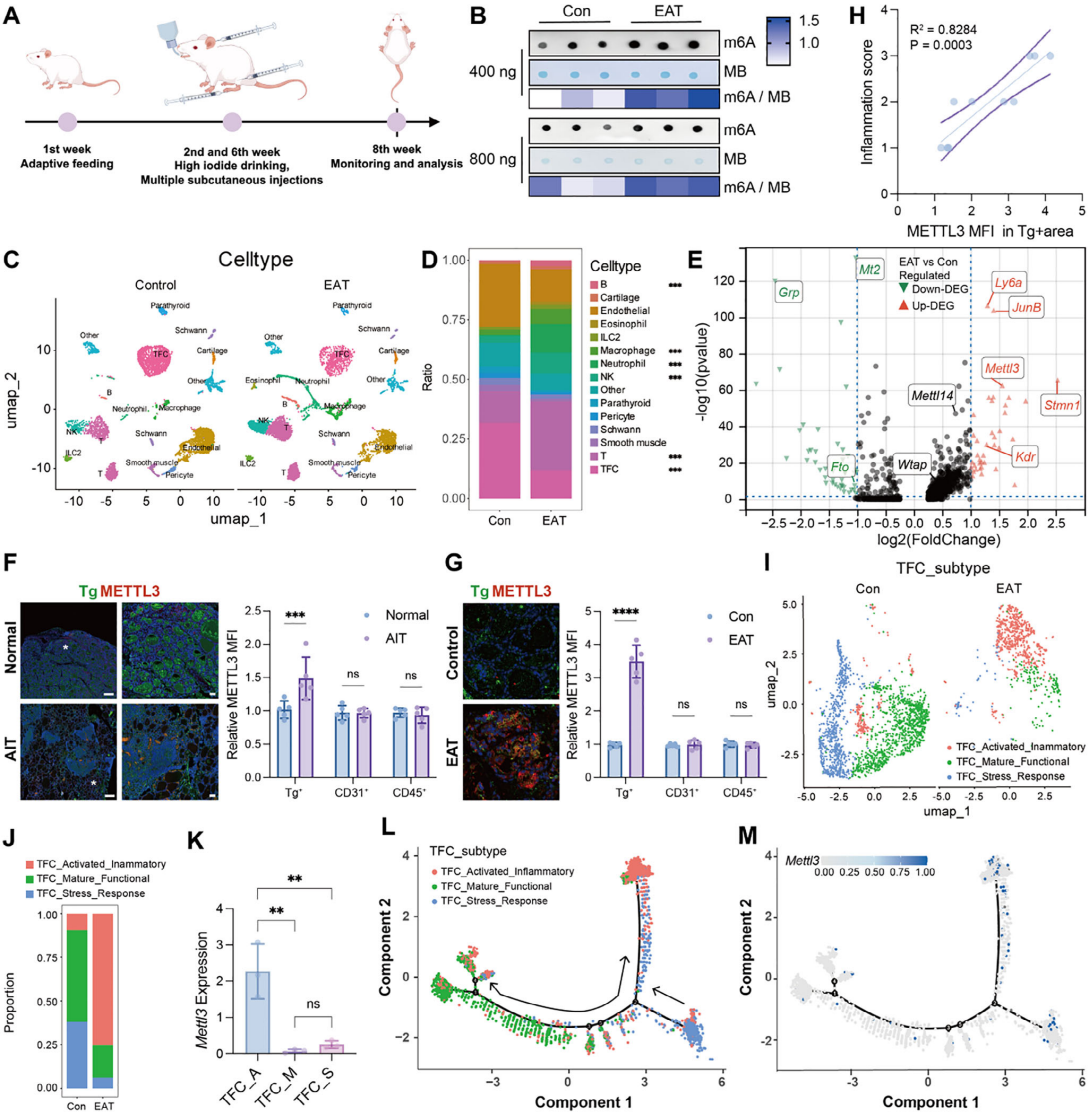
Autoimmune thyroiditis is associated with elevated m6A methylation and METTL3‐driven inflammatory reprogramming of TFCs. (A) Schematic overview of the experimental workflow used to establish the EAT model. (B) Dot blot analysis (*n* = 3) showing increased total m6A RNA methylation in thyroid tissues from EAT mice relative to controls. (C) UMAP projection of scRNA‐seq data from thyroid tissues of control and EAT mice (*n* = 3). (D) Cell composition analysis showing marked expansion of infiltrating immune populations. (E) Volcano plot of differentially expressed genes of TFCs in EAT mice compared with controls. (F) Representative METTL3/Tg co‐immunostaining in AIT and normal thyroid tissues, with quantitative analysis of relative METTL3 MFI (*n* = 10). Scale bars, 200 µm for low‐magnification images and 50 µm for high‐magnification images. White asterisks in the low‐magnification panels indicate the areas shown at higher magnification. (G) Representative METTL3/Tg co‐immunostaining in thyroid tissues from EAT and control mice, and relative METTL3 MFI quantitative analysis (*n* = 5). Scale bars, 50 µm. (H) METTL3 MFI in TFCs positively correlates with the degree of lymphocytic infiltration, data are presented as Mean ± 95% CI. (I, J) TFCs subclustering identified three major states: Mature, Stress, and Inflammatory. (K) *Mettl3* expression is preferentially elevated in the Inflammatory TFCs subcluster. (L) Pseudotime analysis reconstructing the trajectory of TFCs state transition from Mature to Inflammatory, with Stress TFCs occupying an intermediate branch. (M) *Mettl3* dynamics along pseudotime showing progressive upregulation trend toward late pseudotime. Data information: Data are presented as mean ± SD or as appropriate. Statistical analyses were performed using unpaired two‐tailed Student's t‐test (B, E, F, G) or one‐way ANOVA with Tukey's post hoc test (K), as appropriate. Correlations were analyzed using Spearman's rank correlation test (H). ns, not significant; *p < 0.05; **p < 0.01; ***p < 0.001; ****P < 0.0001.

To determine whether m^6^A modification is involved in AIT, we established a rodent model of experimental autoimmune thyroiditis (EAT) [16, 17] (Figure 1A). Elevated plasma TgAb and TPOAb levels, increased thyroid weight and H&E staining showing follicular structure disruption and lymphocyte infiltration confirmed the successful establishment of the EAT model (Figure S1A–D and Table S1). To further establish the translational relevance of the EAT model, we compared thyroid histopathology between patients with autoimmune thyroiditis (AIT) and normal controls (Figure S1E and Table S2). Human AIT thyroid tissues showed substantial TFCs destruction and massive lymphocytic infiltration, closely resembling the pathological alterations observed in the EAT model, whereas normal thyroid tissues maintained intact follicular structure and preserved colloid. These results demonstrate that the EAT model closely phenocopies the core histopathological manifestations of human AIT. In the thyroid tissues of EAT mice, ELISA (Figure S1F) and dot blot analysis (Figure 1B) revealed significantly increased m^6^A RNA modifications levels compared with the control group.

To minimize confounding effects from non‐thyroid cell populations on gene expression changes, we performed single‐cell RNA sequencing (scRNA‐seq) on thyroid tissues from EAT and control mice (Figure 1C). Compared with controls, EAT thyroids exhibited markedly increased immune cell infiltration, characterized by elevated proportions of macrophages, neutrophils, T cells, B cells, and NK cells (Figure 1D). Given that m6A modification is dynamically regulated by methyltransferases (“writers”) and demethylases (“erasers”), we first examined the expression of key m6A regulators in TFCs. Differential expression analysis of the TFC cluster revealed that, relative to controls, *Mettl3* was significantly upregulated, whereas *Fto* was downregulated in EAT‐derived TFCs (Figure 1E).

To further define the cell type‐specific alteration of *Mettl3* in the thyroid, we isolated CD45^+^ immune cells and CD31^+^ endothelial cells from freshly harvested mouse thyroid tissues by flow cytometric sorting, and EpCAM^+^ epithelial cells were subsequently collected from the CD45^−^ CD31^−^ fraction (Figure S2A). To verify cell identity and sorting purity, the isolated populations were subjected to RT‐qPCR analysis of *Tpo*, *Tg*, *Ptprc* (CD45) and *Pecam1* (CD31). *Tpo* and *Tg* were highly expressed in EpCAM^+^ epithelial cells, but were barely detectable in CD45^+^ or CD31^+^ populations, confirming that the sorted EpCAM^+^ epithelial cells from thyroid tissues were predominantly TFCs (Figure S2B). RT‐qPCR and western blot analyses further showed that *Mettl3/*METTL3 was specifically upregulated in EpCAM^+^ TFCs from the EAT group, but not in CD45^+^ immune cells or CD31^+^ endothelial cells (Figure S2C,D). To further localize the aberrant expression of METTL3, we performed co‐immunostaining of METTL3 with Tg (a thyroid follicular cell marker), CD31 (an endothelial cell marker), and CD45 (an immune cell marker) in thyroid tissues from patients with AIT (Figure 1F; Figure S2E) and EAT mice (Figure 1G; Figure S2F). These analyses consistently demonstrated that the abnormal upregulation of METTL3 was predominantly confined to TFCs rather than endothelial or immune cells. Moreover, METTL3 MFI within TFCs was positively correlated with the degree of lymphocytic infiltration (Figure 1H).

Subcluster analysis of the scRNA‐seq data resolved TFCs into three distinct states, termed Mature, Stress, and Inflammatory (Figure 1I,J; Figure S2G,H). Notably, the Mature and Stress subclusters were predominantly enriched in the control group, whereas TFCs in the EAT group were almost exclusively assigned to the Inflammatory subcluster, indicating that EAT is characterized not merely by a reduction in TFCs number, but by a global shift of TFCs toward an inflammatory phenotype. Consistently, *Mettl3* expression was further elevated in the Inflammatory subcluster (Figure 1K, Figure S2I). We next performed pseudotime trajectory analysis of TFCs to reconstruct their state transition during disease progression (Figure 1L). Mature TFCs were positioned at the root of the trajectory, Stress TFCs formed an intermediate branch, and the trajectory ultimately converged on the terminal Inflammatory state. Dynamic gene expression analysis along the trajectory revealed that *Mettl3* expression showed an increasing trend toward late pseudotime (Figure 1M).

### 2.2 METTL3 Depletion Alleviates IL‐17A/Th17 Response and Thyroid Injury

**FIGURE 2:**
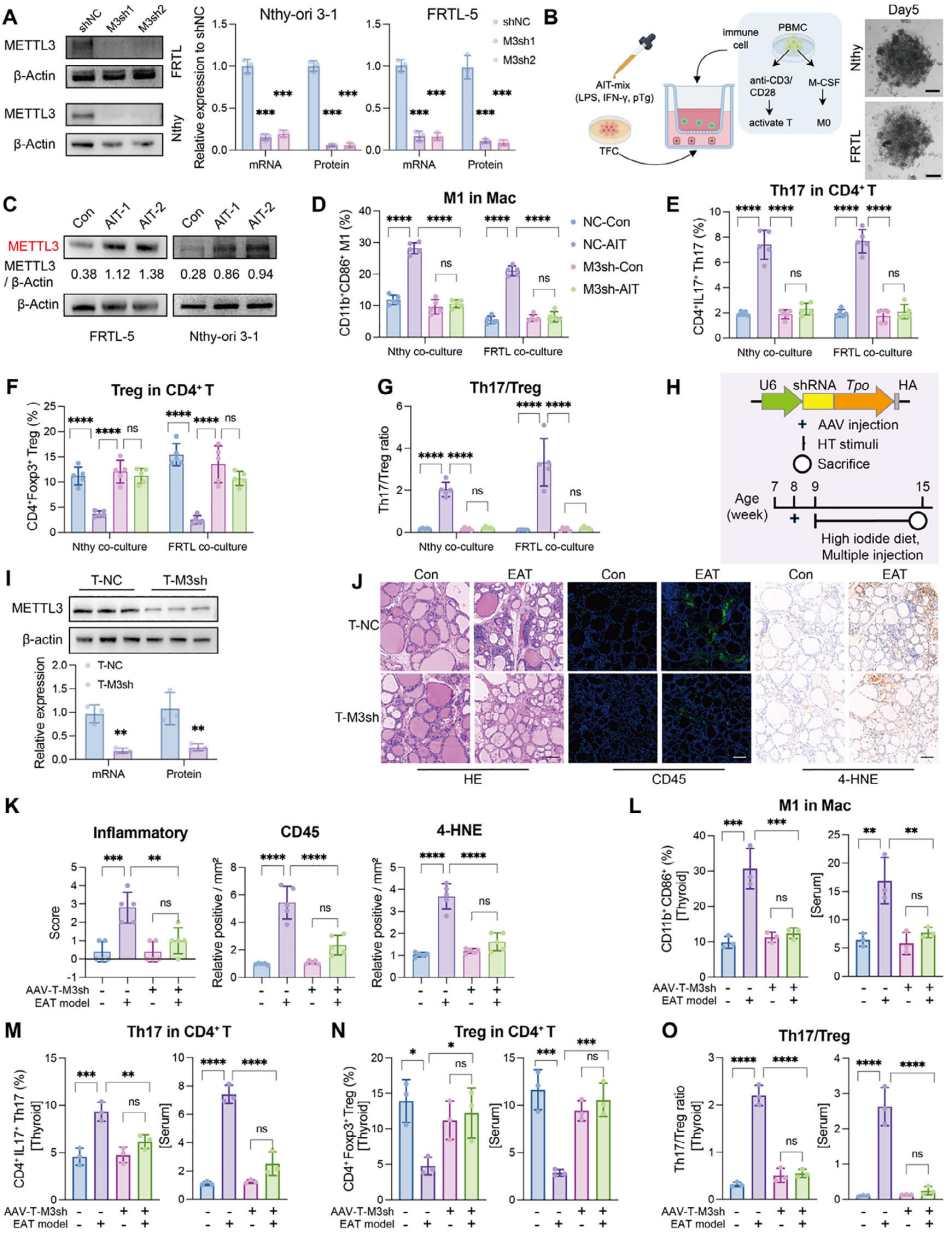
TFC‐intrinsic METTL3 drives inflammatory immune remodeling in autoimmune thyroiditis. (A) RT‐qPCR and western blot validation of shRNA‐mediated METTL3 knockdown in TFCs. (B) Schematic of the 3D biomimetic thyroid follicle co‐culture system used to model the inflammatory microenvironment of autoimmune thyroiditis. TFCs were stimulated with AIT‐mix, consisting of LPS, IFN‐γ, and pTg peptide antigen, then co‐cultured with syngeneic BMMCs and activated splenic cells. Scale bars, 500 µm. (C) METTL3 expression in TFCs after AIT‐mix stimulation. (D) Flow cytometric analysis showing macrophage M1/M2 polarization in co‐culture system. (E–G) flow cytometric analysis showing Th17/Treg balance in co‐culture system. (H) Schematic of the thyroid‐targeting AAV8‐U6‐*Tpo*‐sh*Mettl3* vector (T‐M3sh) used for in vivo *Mettl3* knockdown. (I) RT‐qPCR and western blot validation demonstrating efficient and selective METTL3 knockdown in TFCs. (J,K) Representative H&E, CD45 and 4‐HNE staining, and quantitative analysis showing that thyroid‐specific *Mettl3* knockdown attenuated thyroid tissue injury, immune infiltration, and oxidative stress in EAT mice. Scale bars, 100 µm. (L) Flow cytometric analysis showing that thyroid‐specific *Mettl3* knockdown reduced M1 polarization in vivo. (M–O) Flow cytometric analysis showing that thyroid‐specific *Mettl3* knockdown restored Th17/Treg balance in vivo. **Data information**: Values are presented as mean ± SD, *n* = 5. Statistical analyses were performed using unpaired two‐tailed Student's t‐test for I, and one‐way ANOVA with Tukey's post hoc test for others. ns, not significant; *p < 0.05; **p < 0.01; ***p < 0.001; ****p < 0.0001.

To investigate the functional role of METTL3 in AIT, we silenced its endogenous expression in TFCs using shRNAs (Figure 2A). A 3D biomimetic thyroid follicle co‐culture system was then established to mimic the inflammatory microenvironment of AIT (Figure 2B). Specifically, TFCs were stimulated with an AIT‐mix, including lipopolysaccharide (LPS), interferon‐γ (IFN‐γ), and porcine thyroglobulin (pTg) peptide antigen to induce innate immune activation. Upon treatment with the AIT‐mix, both METTL3 expression (Figure 2C; Figure S3A) and global m6A level (Figure S3B,C) were markedly increased. Disease‐associated cellular injury was further evaluated by measuring thyroid cell viability (Figure S3D) and intracellular ROS levels (Figure S3E).

PBMC‐derived monocytes were differentiated into M0 macrophages using M‐CSF, while PBMC‐derived T cells were pre‐activated with anti‐CD3/CD28. These immune cells were then seeded in the upper chamber of the Transwell co‐culture system, whereas AIT‐mix‐treated TFCs were plated in the lower chamber to assess the effects of inflamed TFCs on Th17/Treg balance and macrophage M1 polarization during the early stage of AIT. Flow cytometric analysis showed that co‐culture with AIT‐treated TFCs significantly increased the proportion of M1 macrophages (CD11b^+^ CD86^+^) (Figure 2D; Figure S3F) and induced a shift in the Th17/Treg balance toward a proinflammatory state, as evidenced by increased proportions of Th17 cells (CD4^+^ IL‐17A^+^) and an elevated Th17/Treg ratio (Figure 2E–G; Figure S3G,H). In addition, the levels of Th1‐ and Th17‐associated inflammatory cytokines, including IL‐6 and IL‐17A, were markedly increased in the culture supernatant (Figure S3I). In contrast, METTL3 knockdown in TFCs substantially attenuated these proinflammatory phenotypes in the co‐cultured immune cells.

To validate the role of METTL3 in vivo, we generated thyroid‐specific *Mettl3* knockdown mice using AAV8‐U6‐*Tpo*‐sh*Mettl3* (T‐M3sh), and littermate controls (AAV8‐U6‐*Tpo*‐Scramble, T‐NC) (Figure 2H). Flow‐based isolation of EpCAM^+^ TFCs, CD45^+^ immune cells, and CD31^+^ endothelial cells, together with marker validation of sorting purity (Figure S4A), confirmed that *Mettl3* knockdown occurred specifically in TFCs, but not in immune or endothelial cells (Figure 2I; Figure S4B). Functionally, thyroid‐specific *Mettl3* silencing markedly attenuated EAT‐induced thyroid injury, including tissue destruction, inflammatory infiltration, and ROS accumulation (Figure 2J,K), and also reduced circulating autoantibody levels (Figure S4C).

Flow cytometric analysis demonstrated that thyroid‐specific *Mettl3* knockdown markedly attenuated M1 macrophage polarization and rebalanced the Th17/Treg axis in both the thyroid immune microenvironment and peripheral blood (Figure 2L–O; Figure S4D–F). In line with this, serum IL‐6 and IL‐17A levels were significantly decreased in T‐M3sh EAT mice relative to T‐NC EAT controls (Figure S4G). Moreover, RT‐qPCR analysis of spleen and thyroid tissues showed that the EAT‐associated decrease in *Foxp3* and increases in *Ror‐γt* and *Il17a* were partially reversed by T‐M3sh treatment (Figure S4H,I). Together, these findings demonstrate that METTL3 silencing in TFCs alleviates local and systemic immune‐inflammatory imbalance in EAT.

To further elucidate the regulatory effects of METTL3 on thyroid inflammation and immune responses, we performed RNA sequencing (RNA‐seq) and Gene Ontology (GO) enrichment analyses in EAT mice treated with T‐M3sh or T‐NC mice (Figure S5A,B). DEGs were mainly enriched in pathways related to ribonucleoprotein complex biogenesis, protein‐RNA complex organization, and mitochondrial gene expression (Figure S5C). KEGG analysis showed that the Notch signaling pathway, Wnt signaling pathway, and calcium signaling pathway were enriched (Figure S5D). GSEA revealed that *Mettl3* knockdown was negatively associated with vascular development (ES = −0.4496, p = 0.0040), cytokine–cytokine receptor interaction (ES = −0.3281, p = 0.0012), chemokine signaling (ES = −0.6091, p = 0.0221), and NF‐κB signaling (ES = −0.3353, p = 0.0311) (Figure S5E).

### 2.3 ROS Promotes METTL3‐Dependent m6A Modification Through SIRT1‐Mediated Epigenetic Regulation

**FIGURE 3:**
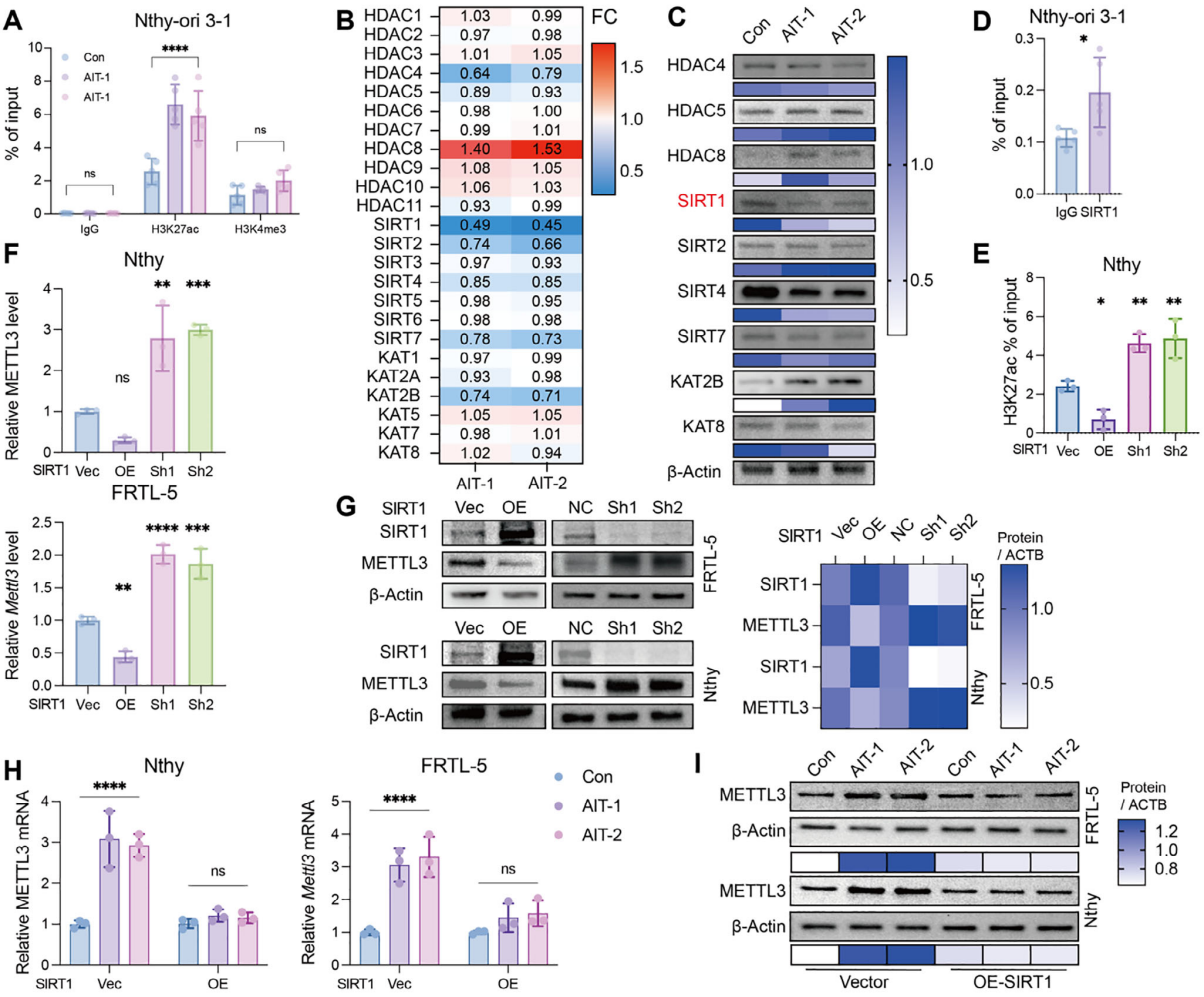
Loss of SIRT1 promotes H3K27ac‐dependent transcriptional activation of METTL3 in TFCs. (A) ChIP‐qPCR analysis showing enrichment of H3K4me3 and H3K27ac at the METTL3 promoter. H3K27ac occupancy was further increased after AIT‐mix treatment. (B) Heatmap showing fold changes in mRNA expression of histone acetylation regulators in TFCs after AIT‐mix treatment relative to the control group. Among the differentially expressed regulators, SIRT1 showed the most prominent downregulation. (C) Western blot validation of reduced SIRT1 protein expression in AIT‐mix‐treated TFCs. (D) ChIP‐qPCR analysis showing no significant enrichment of SIRT1 at the METTL3 promoter. (E) Modulation of SIRT1 expression altered H3K27ac enrichment at the METTL3 promoter, with SIRT1 overexpression reducing and SIRT1 knockdown enhancing local H3K27ac signals. (F, G) SIRT1 overexpression suppressed, whereas SIRT1 knockdown increased, METTL3/*Mettl3* mRNA and protein expression in TFCs. (H, I) SIRT1 overexpression attenuated AIT‐mix‐induced METTL3/*Mettl3* upregulation. **Data information**: Data are presented as mean ± SD. Statistical analyses were performed using unpaired two‐tailed Student's t‐test for D, and one‐way ANOVA with Tukey's post hoc test for others. ns, not significant; *p < 0.05; **p < 0.01; ***p < 0.001; ****p < 0.0001.

Thyroid hormone synthesis depends on ROS‐mediated iodide oxidation, making the thyroid gland inherently vulnerable to oxidative stress [18]. As cellular stress has been reported to reshape m6A landscapes, we examined whether oxidative stress regulates METTL3 expression in TFCs. Both mRNA and protein levels increased progressively after H_2_O_2_ exposure for 6, 12, and 24 h (Figure S6). To explore the underlying mechanism, we analyzed the METTL3 promoter region and identified substantial enrichment of the transcription‐activating histone marks H3K27ac and H3K4me3 (Figure S7). Notably, AIT‐mix treatment further enhanced H3K27ac occupancy at the METTL3 promoter, as confirmed by ChIP‐qPCR in Nthy‐ori 3‐1 cells (Figure 3A). Histone acetyltransferases (HATs) and histone deacetylases (HDACs) are key regulators of gene transcription [19]. We examined the effect of AIT‐mix on the mRNA levels of histone acetylation regulators in Nthy‐ori 3‐1 (Figure 3B). Compared with the control, AIT‐mix reduced the expression levels of HDAC4, HDAC5, SIRT1, SIRT2, SIRT4, SIRT7, and KAT2B, while increasing HDAC8 expression, with the most significant change observed in SIRT1. Western blot analysis showed that AIT‐mix downregulated SIRT1 protein levels in TFCs (Figure 3C).

We next asked whether SIRT1 mediates the epigenetic regulation of METTL3. Although SIRT1 was not significantly enriched at the METTL3 promoter, as determined by ChIP‐qPCR (Figure 3D), SIRT1 overexpression significantly reduced H3K27ac occupancy at the METTL3 promoter and suppressed the expression (Figure 3E–G). In contrast, SIRT1 silencing enhanced promoter H3K27ac enrichment and increased METTL3 expression. Notably, enforced SIRT1 expression also blunted the EAT‐induced upregulation of METTL3 (Figure 3H,I). Given that oxidative stress in AIT is associated with SIRT1 downregulation, these findings support a model in which the oxidative microenvironment promotes METTL3 expression through SIRT1 loss‐driven H3K27ac activation at its promoter.

To determine whether the inhibitory effect of SIRT1 on METTL3 depends on its deacetylase activity, we reconstituted wild‐type SIRT1 (WT) or the deacetylase‐deficient H363Y mutant [20, 21] in SIRT1‐knockdown cells. Whereas SIRT1‐WT markedly reduced H3K27ac enrichment at the METTL3 promoter and downregulated METTL3 expression, the H363Y mutant showed minimal effects on either promoter acetylation or METTL3 expression (Figure S8). Together, these data demonstrate that SIRT1 suppresses METTL3 transcription by restraining promoter H3K27ac in a deacetylase‐dependent manner.

### 2.4 METTL3 Mediates KDR m^6^A Modification in a YTHDC2‐Dependent Manner

**FIGURE 4:**
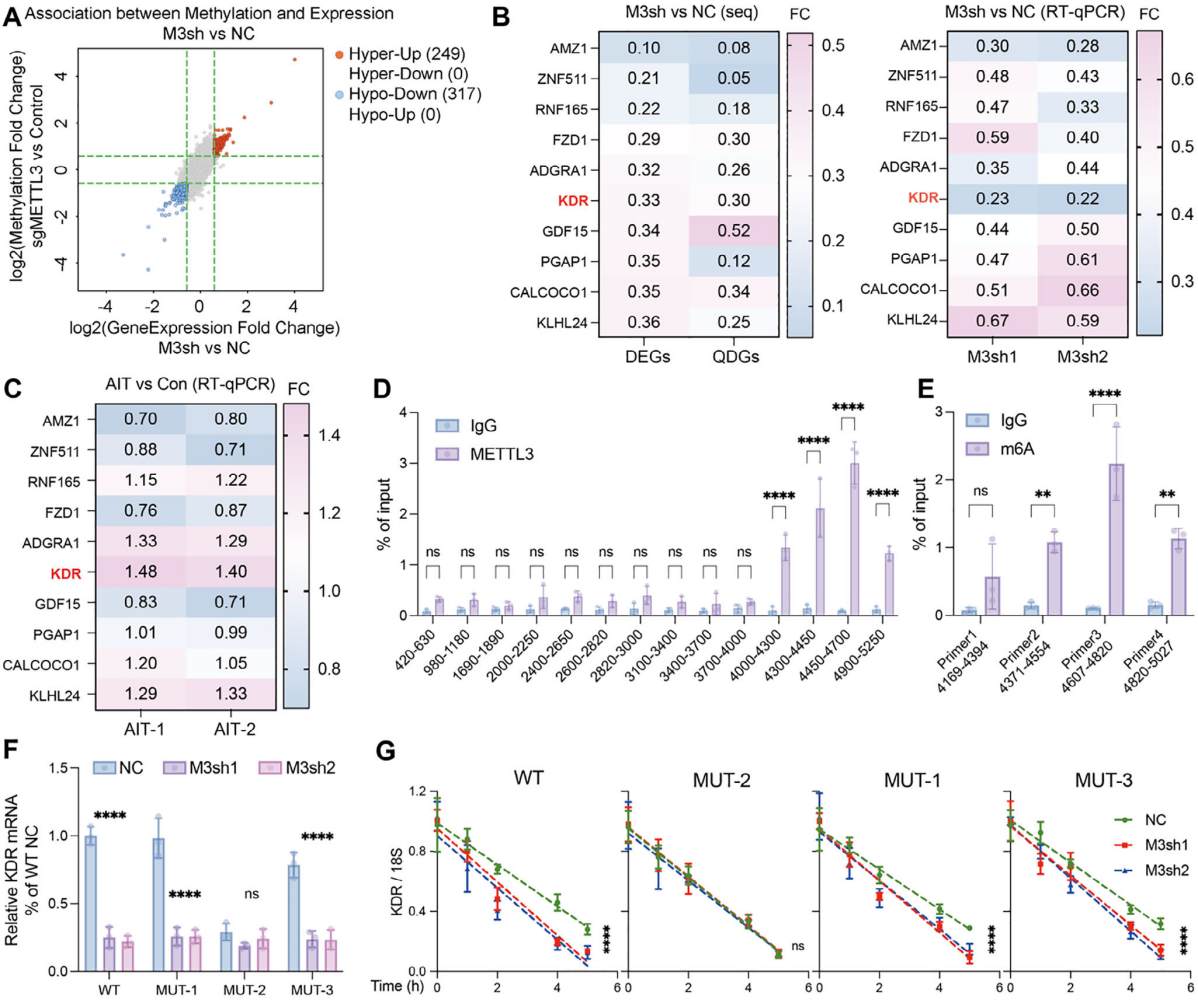
METTL3 promotes KDR expression by installing m6A modification at a critical site and enhancing KDR mRNA stability in Nthy‐ori 3‐1 cells. (A) Integrated analysis of m6A epitranscriptomic microarray and transcriptomic profiling data from NC and M3sh Nthy‐ori 3‐1 cells (*n* = 3), showing the association between differential quantitative m6A methylation genes (QDGs) and differential expression genes (DEGs). (B) Validation of the top 10 overlapping candidate transcripts (AMZ1, ZNF511, RNF165, FZD1, ADGRA1, KDR, GDF15, PGAP1, CALCOCO1, and KLHL24) identified from the DEGs and QDGs. Fold changes from sequencing data and RT‐qPCR validation are shown as heatmap. (C) RT‐qPCR analysis of the indicated candidate genes in TFCs after AIT‐mix treatment relative to control. (D) RIP assay confirming direct binding of METTL3 protein to KDR mRNA in TFCs. (E) MeRIP‐qPCR analysis of predicted m6A‐modified regions on KDR mRNA showing that m6A enrichment was highest in the region amplified by primer pair 3. This region overlapped with the METTL3‐binding region identified by RIP. (F) Site‐directed mutational analysis in KDR‐silenced cells reconstituted with wild‐type KDR or three site‐mutant constructs. METTL3 knockdown reduced KDR mRNA levels in the WT, site1M, and site3M groups, but not in the site2M group. (G) RNA stability assay showing that the effect of M3sh on KDR mRNA stability was abolished only when site 2 was disrupted, data are presented as Mean ± 95% CI. Data information: Data are presented as mean ± SD or as appropriate, *n* = 3. Statistical significance of D, E, and F was determined using unpaired two‐tailed Student's t‐test. Statistical significance of B, C, and G was using one‐way ANOVA followed by Tukey's multiple‐comparisons test for comparisons among multiple groups. And statistical significance of H was using two‐way ANOVA for time‐course analyses where applicable. ns, not significant; *p < 0.05; **p < 0.01; ***p < 0.001; ****p < 0.0001.

To further elucidate the downstream mechanisms by which METTL3 promotes AIT progression, we performed an m^6^A‐mRNA & lncRNA epitranscriptomic microarray using METTL3‐knockdown (M3sh) and control (NC) Nthy‐ori 3‐1 thyroid cells (*n* = 3). The results revealed that 249 genes exhibited hyper‐m^6^A modification accompanied by upregulated expression, whereas 317 genes showed hypo‐m^6^A modification with downregulated expression (Figure 4A; Figure S9A–D). The top 10 overlapping transcripts (AMZ1, ZNF511, RNF165, FZD1, ADGRA1, KDR, GDF15, PGAP1, CALCOCO1, and KLHL24) were consistently downregulated following METTL3 knockdown (Figure 4B). Among these, KDR displayed the most significant and pronounced change. In addition, AIT‐mix treatment upregulated the mRNA levels of RNF165, ADGRA1, KDR, and KLHL24 in TFCs (Figure 4C). KDR, also known as vascular endothelial growth factor receptor 2 (VEGFR2), is predominantly expressed in endothelial cells but is also present in non‐vascular tissues, including the thyroid gland [22]. These findings suggest that KDR may serve as a potential downstream target of METTL3. Because KDR is canonically expressed in endothelial cells, we examined the cellular source of its aberrant expression in AIT thyroid tissues by co‐immunostaining with Tg, CD31, and CD45. In both patients with AIT and EAT mice, the increased KDR signal was found predominantly in TFCs rather than endothelial or immune cells (Figure S9F,G).

Western blot analysis showed that KDR was downregulated upon METTL3 knockdown in TFCs (Figure S10A). Functionally, KDR overexpression rescued the inhibitory effects of METTL3 silencing on AIT‐mix‐induced cellular phenotypes, supporting the notion that METTL3 drives AIT progression at least partly through KDR (Figure S10B–I). In addition, wild‐type METTL3, but not the catalytically inactive D395A mutant, increased KDR expression (Figure S10J,K), indicating that this regulation is m6A‐dependent. Whereas CHX treatment did not reveal a substantial effect on KDR protein stability (Figure S10L), actinomycin D assays showed that KDR/*Kdr* mRNA decay was accelerated in METTL3‐knockdown TFCs (Figure S10M), suggesting that METTL3 primarily maintains KDR expression by enhancing mRNA stability. RIP assays confirmed the direct binding of METTL3 to KDR mRNA (Figure 4D). SRAMP analysis predicted multiple candidate m6A sites within the KDR transcript (Figure S11A and Table S3), and four MeRIP‐qPCR primer pairs were designed to cover 13 predicted sites (Figure S11B). Among these regions, the fragment amplified by primer pair 3 exhibited the highest m6A enrichment in TFCs. Importantly, this region coincided with the METTL3‐binding site identified by RIP, supporting the idea that METTL3 directly targets this region to install m6A modification on KDR mRNA (Figure 4E).

To pinpoint the functional site, three mutant plasmids were generated targeting the three putative m^6^A sites within this region (Figure S11C). We then overexpressed wild‐type KDR or three mutant constructs in KDR‐knockdown cells. METTL3 silencing reduced KDR mRNA levels in the WT, site 1 mutant, and site 3 mutant groups, but this effect was completely lost in the site 2 mutant, which disrupts the conserved +4731 m6A site (Figure 4F). Likewise, the effect of METTL3 knockdown on KDR mRNA stability disappeared only when site 2 was mutated (Figure 4G). These data indicate that METTL3 stabilizes KDR mRNA by installing m6A modification at the conserved +4731 site.

Regarding the m^6^A reader, PRIdictor analysis predicted a high‐affinity binding interaction between YTHDC2 and KDR mRNA, with the predicted binding region overlapping the identified m^6^A‐modified segment (Figure S12). YTHDC2 is a well‐established m^6^A reader that can bind thousands of m^6^A‐modified mRNAs to modulate their stability. Two independent shRNAs were used to silence YTHDC2, which significantly decreased both KDR mRNA and protein levels (Figure S13A,B). After actinomycin D treatment, YTHDC2 knockdown accelerated KDR mRNA decay, supporting a role for YTHDC2 in maintaining KDR mRNA stability (Figure S13C). Consistently, RIP assays showed that YTHDC2 was enriched at the m6A‐modified region of the KDR transcript, and this binding was diminished by METTL3 silencing (Figure S13D). Functionally, YTHDC2 knockdown abolished the ability of METTL3 to enhance KDR expression and mRNA stability (Figure S13E,F). Multiple sequence alignment of human, rat, and mouse KDR/*Kdr* transcripts showed that the DRACH motif encompassing the human +4731 m6A site is highly conserved across species (Figure S14A). Importantly, experiments in rat TFCs independently validated the METTL3‐binding site and the corresponding m6A‐modified region on KDR/*Kdr* mRNA (Figure S14B–G), indicating that this METTL3‐KDR m6A regulatory axis is evolutionarily conserved.

To further establish the functional importance of the +4731 m6A site in METTL3–YTHDC2‐dependent KDR regulation, we overexpressed either wild‐type WT or the +4731‐deficient mutant (MUT‐2) in KDR/*Kdr*‐knockdown TFCs (Figure S15A). METTL3 silencing reduced KDR/*Kdr* mRNA stability and m6A enrichment in the WT group, but had no detectable effect in the MUT‐2 group (Figure S15B,C). Likewise, AIT‐mix treatment induced KDR/*Kdr* upregulation in WT‐reconstituted cells, whereas this response was largely lost in the MUT‐2 group (Figure S15D). These data demonstrate that AIT‐mix/METTL3‐YTHDC2 regulates KDR expression through m6A modification at the conserved +4731 site.

### 2.5 METTL3 Enhances KDR‐VEGFA Signaling and Induce IL‐17A/Th17 Response

**FIGURE 5:**
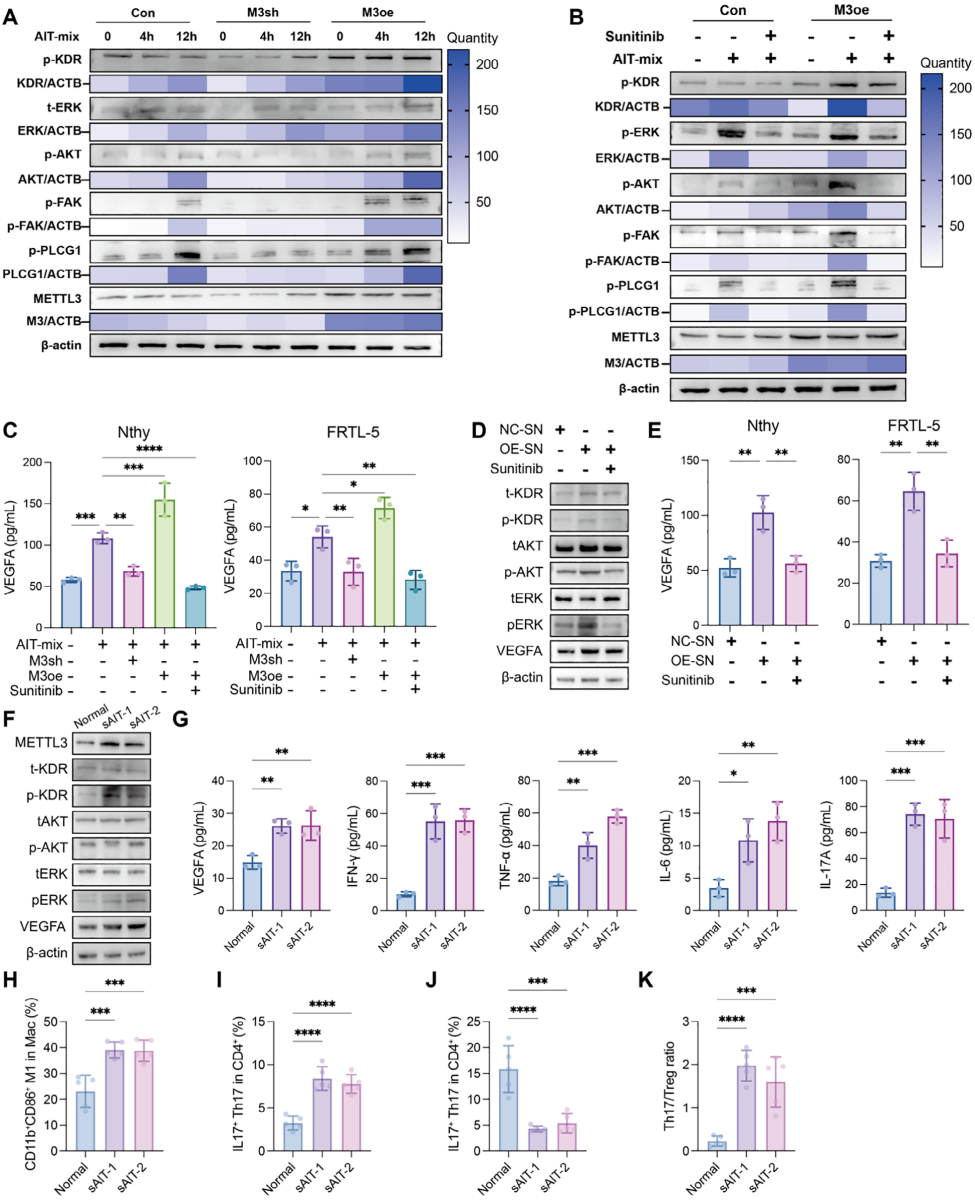
METTL3 amplifies a KDR‐VEGFA autocrine signaling loop to promote inflammatory activation of thyroid follicular cells. (A) Western blot analysis and corresponding heatmap quantification showing time‐dependent activation of KDR and its downstream kinases, including ERK1/2, AKT, FAK, p38, and PLCγ, in TFCs after AIT‐mix treatment. These signaling events were attenuated by METTL3 silencing and further enhanced by METTL3 overexpression. (B) Western blot analysis and heatmap quantification showing that pretreatment with sunitinib reduced AIT‐mix‐induced phosphorylation of KDR, ERK1/2, and AKT. (C) ELISA analysis showing that AIT‐mix treatment increased VEGFA secretion in TFCs supernatants, which was attenuated by METTL3 silencing or sunitinib pretreatment and further enhanced by METTL3 overexpression. (D) Western blot analysis showing that CM collected from AIT‐mix‐treated METTL3‐overexpressing TFCs activated KDR signaling in untreated recipient TFCs. This effect was abolished by sunitinib treatment. (E) ELISA analysis showing that conditioned medium from AIT‐mix‐treated METTL3‐overexpressing TFCs enhanced VEGFA secretion in recipient TFCs, and this effect was blocked by sunitinib. (F) Expression analysis of METTL3 and KDR in a 3D TFC model stimulated with serum from AIT patients, using corresponding healthy serum as controls. (G) ELISA analysis of culture supernatants showing that sAIT increased the secretion of VEGFA and TNF‐α, IFN‐γ, IL‐6, and IL‐17A in the 3D TFC model. (H) Flow cytometric quantitative analysis of immune cells in the co‐culture system showing that sAIT promoted M1 macrophage polarization. (I‐K) Flow cytometric quantitative analysis of immune cells in the co‐culture system showing that sAIT increased the proportion of Th17 cells, decreased Treg proportion, and increased Th17/Treg ratio. **Data information**: Data are presented as mean ± SD, *n* = 3. Statistical significance was determined using one‐way ANOVA followed by Tukey's multiple‐comparisons test for comparisons among multiple groups, and two‐way ANOVA where applicable. ns, not significant; *p < 0.05; **p < 0.01; ***p < 0.001; ****p < 0.0001.

KDR serves as the primary receptor mediating VEGF‐A signaling. The changes in KDR phosphorylation and its downstream signaling pathways were examined in TFCs with or without AIT‐mix treatment. Immunofluorescence staining of phosphorylated KDR (p‐KDR) showed that AIT‐mix markedly increased KDR activation, whereas this enhancement was substantially attenuated in M3sh‐TFCs (Figure S16A). Besides, the phosphorylation of KDR and its downstream kinases, including ERK, AKT, FAK, p38, and PLCγ, was activated under AIT‐mix treatment, in a time‐dependent manner (Figure 5A). These effects were reversed by METTL3 silencing and further amplified by METTL3 overexpression. Pretreatment with sunitinib, a multi‐target tyrosine kinase inhibitor that selectively blocks KDR, reduced AIT‐mix induced phosphorylation of KDR, ERK1/2, and AKT (Figure 5B). Interestingly, AIT‐mix treatment increased VEGFA secretion and VEGFA/*Vegfa* gene expression in TFCs (Figure 5C; Figure S16B,C). Consistently, conditioned medium from AIT‐mix‐treated TFCs significantly promoted HUVEC tube formation on Matrigel compared with control medium (Figure S16D). This pro‐angiogenic effect was attenuated by METTL3 silencing, indicating that METTL3 contributes to the angiogenic activity of inflamed TFCs.

As KDR‐VEGFA signaling has been reported to form a self‐amplifying feedback loop, we next examined whether this mechanism operates in AIT. rhVEGFA induced a time‐activation of KDR downstream signaling, which was weakened by METTL3 knockdown and enhanced by METTL3 overexpression. Consistently, sunitinib pretreatment markedly blocked rhVEGFA‐induced KDR activation (Figure S16E). Moreover, conditioned medium from AIT‐mix‐treated M3oe‐TFCs activated KDR signaling and promoted VEGFA secretion in untreated recipient TFCs (Figure 5D,E), and both effects were abolished by sunitinib. These findings support the existence of an autocrine KDR‐VEGFA positive feedback loop in inflamed TFCs.

To better model the clinical pathophysiological context of AIT, we stimulated Nthy cells in a 3D co‐culture system with serum from patients with AIT (sAIT), using healthy serum as a control. sAIT treatment significantly increased METTL3 and KDR expression in Nthy cells (Figure 5F), enhanced VEGFA and inflammatory cytokine secretion (Figure 5G), promoted M1 macrophage polarization (Figure 5H), increased Th17 cell abundance, and reduced Treg cells in the co‐cultured immune compartment (Figure 5I–K). These data support a role for the METTL3‐KDR‐VEGFA axis in sustaining early AIT‐associated inflammatory responses through M1 polarization and IL‐17A/Th17 shifting.

T‐cell subcluster analysis of the scRNA‐seq data showed increased proportions of Th17 cells, CD8^+^ T cells, and activated CD4^+^ T cells, accompanied by a reduction in Treg cells in EAT (Figure S17A,B). CellChat analysis further revealed that, compared with controls, TFCs displayed markedly enhanced interaction strength, but not interaction number, in the EAT microenvironment (Figure S17C). Notably, TFC‐derived signaling toward endothelial cells, T cells, and TFCs themselves was substantially increased (Figure S17D). Hierarchical network analysis identified VEGFA signaling as a prominent communication axis, in which TFCs acted not only as signal receivers but also as major signal senders, with particularly strong interactions involving endothelial cells, T cells, and neutrophils (Figure S17E). These findings support a model in which VEGF signaling functions as a key hub linking vascular remodeling, immune inflammation, and stromal responses in AIT.

### 2.6 Inhibition of the METTL3‐KDR Axis Restores Th17/Treg Balance and Alleviates Thyroid Inflammation

Given the established pathogenic role of IL‐17A/Th17 in AIT and the importance of Th17/Treg imbalance in disease development, we next explored the relationship between the METTL3‐KDR axis and Th17/Treg dysregulation. Flow cytometric analysis of PBMCs showed that patients with AIT had a mildly increased proportion of CD4^+^IL‐17A^+^ Th17 cells, a reduced proportion of CD4^+^FOXP3^+^ Treg cells, and an elevated Th17/Treg ratio compared with healthy controls (Figure S18A–C). In parallel, serum IL‐17A and IL‐6 levels were also increased in patients with AIT (Figure S18D). Consistent with these clinical observations, our earlier data showed that TFC‐specific METTL3 silencing restored Th17/Treg balance, lowered inflammatory cytokine levels, and delayed disease progression in EAT mice (Figure S4).

**FIGURE 6:**
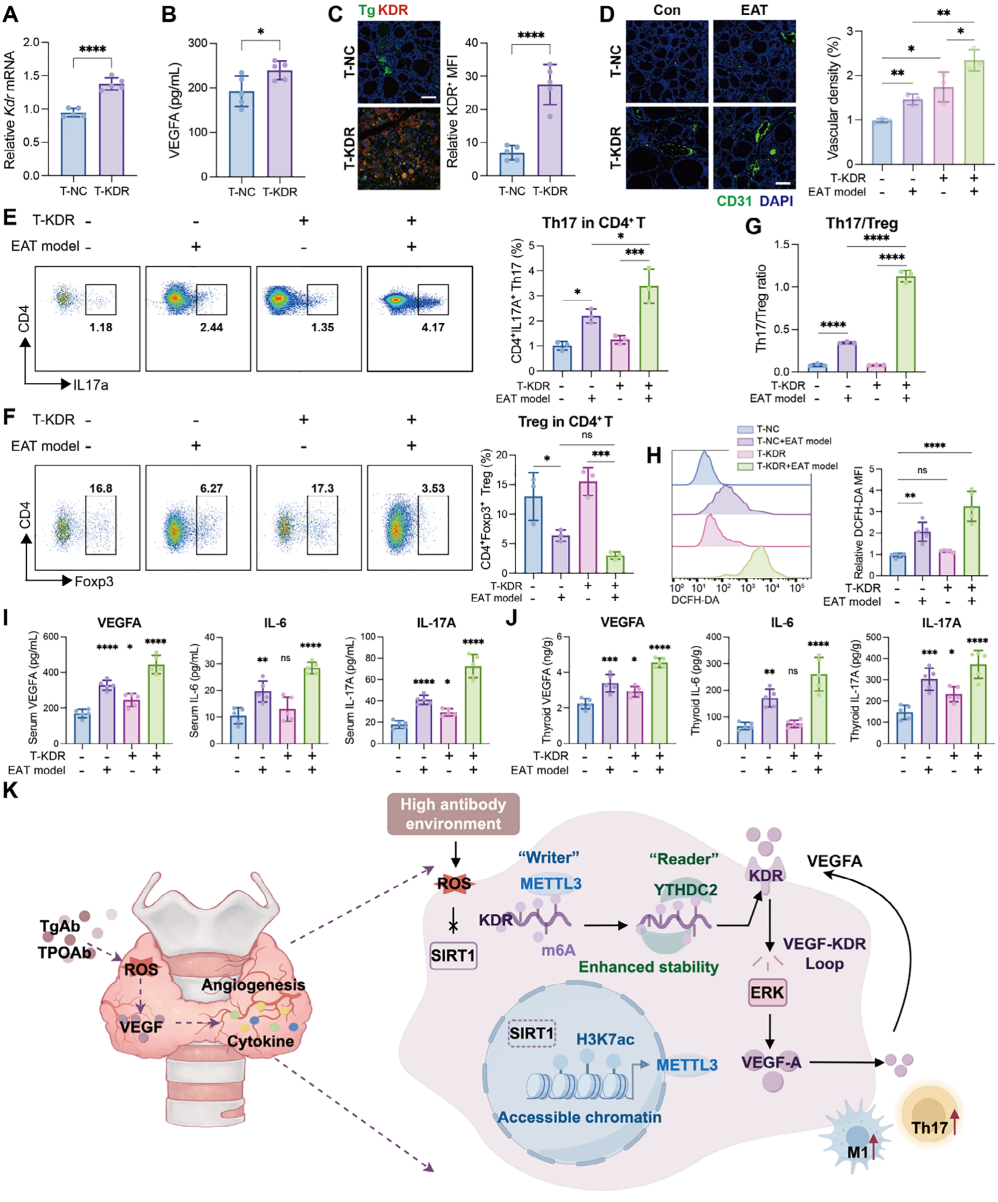
Thyroid‐specific KDR overexpression aggravates oxidative stress, vascular remodeling, and Th17/Treg imbalance in EAT. (A) RT‐qPCR analysis of sorted Tg^+^ TFCs isolated from thyroid tissues of mice injected with control vector (T‐NC) or AAV8‐*Tpo‐Kdr* (T‐KDR), showing successful thyroid‐specific overexpression of *Kdr* before EAT induction. (B) ELISA analysis of culture supernatants from sorted TFCs showing increased VEGFA secretion in T‐KDR group. (C) Representative immunofluorescence co‐staining of KDR and Tg in thyroid tissues confirming increased KDR expression in Tg^+^ thyroid follicular cells after AAV8‐TPO‐KDR administration. Scale bar, 50 µm. (D) Representative IF of CD31 (green) and DAPI (blue) in thyroid tissues showing increased vascular density in the thyroid glands of T‐KDR mice after EAT induction. Scale bar, 50 µm. (E, F) Representative flow cytometry plots and quantification of thyroid‐infiltrating Th17 cells (E) and Treg cells (F) showing that thyroid‐specific KDR overexpression increased the proportion of Th17 cells and reduced the proportion of Treg cells in EAT mice. (G) Quantification of the Th17/Treg ratio in thyroid tissues showing a marked increase in T‐KDR group after EAT induction. (H) Representative flow cytometry plots and quantification of DCFH‐DA fluorescence in thyroid tissues showing increased oxidative stress in T‐KDR mice. (I) ELISA analysis of mouse serum showing that thyroid‐specific KDR overexpression increased circulating VEGFA, IL‐17A, and IL‐6 levels in EAT mice. (J) ELISA analysis of thyroid tissue lysates showing increased local levels of VEGFA, IL‐17A, and IL‐6 in T‐KDR group. (K) Schematic model illustrating that the METTL3‐KDR axis promotes oxidative stress, VEGFA signaling, immune dysregulation, and tissue injury in autoimmune thyroiditis. Genetic or pharmacological inhibition of this pathway restores immune balance and attenuates inflammatory damage, highlighting METTL3 and KDR as potential therapeutic targets (Created by Figdraw). Data information: Data are presented as mean ± SD. Statistical significance was determined using unpaired two‐tailed Student's t‐test for two‐group comparisons (A–C) and one‐way ANOVA followed by Tukey's multiple‐comparisons test for comparisons among multiple groups (D–J), as appropriate. ns, not significant; *p < 0.05; **p < 0.01; ***p < 0.001; ****p < 0.0001.

To investigate the role of KDR activation in sustained thyroid injury during AIT, we induced thyroid‐specific *Kdr* overexpression by tail‐vein delivery of AAV8‐*Tpo*‐*Kdr* (T‐KDR) or T‐NC before EAT induction. Compared with T‐NC, isolated TFCs from T‐KDR mice showed elevated *Kdr* expression and increased VEGFA secretion (Figure 6A,B), which was further confirmed by co‐immunostaining in Tg^+^ thyroid cells (Figure 6C). Upon EAT stimulation, KDR overexpression markedly increased thyroid vascular density (Figure 6D), promoted Th17 cell accumulation, reduced Treg abundance (Figure 6E–G), and enhanced oxidative stress in thyroid tissues (Figure 6H). In parallel, serum and thyroid levels of VEGFA, IL‐17A, and IL‐6 were all significantly elevated (Figure 6I,J). These results demonstrate that thyroid‐specific KDR overexpression exacerbates oxidative stress, Th17/Treg imbalance, and persistent inflammatory injury in EAT.

In line with the genetic data, sunitinib, a pharmacological KDR inhibitor, conferred sustained anti‐inflammatory and antioxidative protection in EAT mice. Sunitinib suppressed p‐KDR and downstream pathway activation in thyroid tissues (Figure S19A), restored vascular homeostasis (Figure S19B), improved endothelial cell viability (Figure S19C), reduced immune cell infiltration (Figure S19D), and lowered VEGFA and inflammatory cytokine levels in both serum and thyroid tissues (Figure S19E,F). Together with the genetic intervention data, these results demonstrate that blockade of the METTL3‐KDR axis re‐establishes Th17/Treg immune balance and mitigates thyroid inflammatory injury. These findings highlight METTL3 and KDR as potential therapeutic targets for AIT (Figure 6K).

### 2.7 Tg‐Targeted DNA Nanoflowers Loaded with a METTL3 PROTAC for AIT Therapy

**FIGURE 7:**
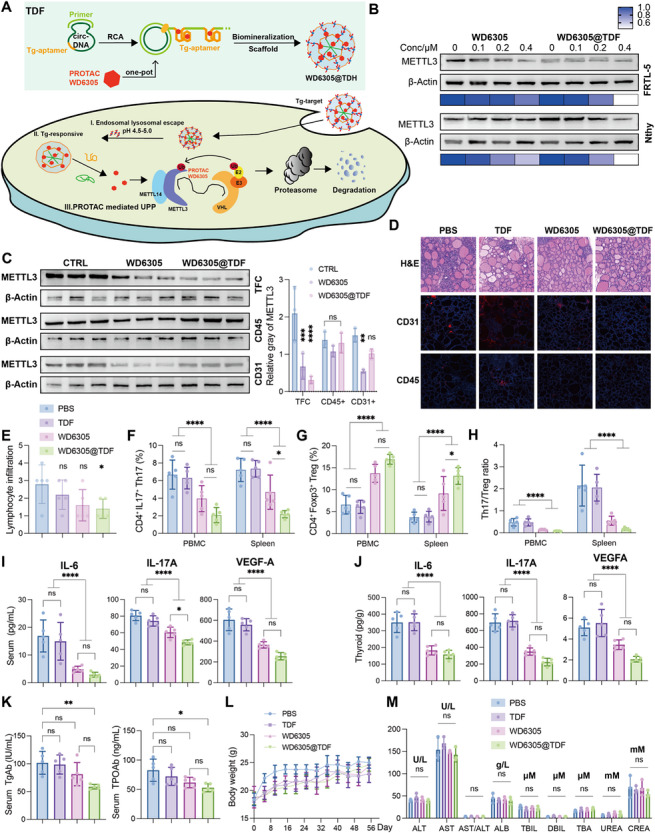
Tg‐targeted delivery of a METTL3 PROTAC selectively degrades METTL3 in TFCs and ameliorates autoimmune thyroiditis. (A) Schematic illustration of the design and working mechanism of WD6305@TDF. The thyroglobulin‐targeted DNA nanoflower (TDF) enables selective delivery of the METTL3 PROTAC degrader WD6305 into Tg‐high thyroid cells, where WD6305 recruits an E3 ubiquitin ligase to induce METTL3 degradation. (B) Western blot analysis of METTL3 protein levels in TFCs treated with WD6305 or WD6305@TDF for 24 h. WD6305 and WD6305@TDF induced dose‐dependent degradation of METTL3 in TFCs. (C) Western blot analysis of sorted TFCs (Tg^+^), immune cells (CD45^+^), and endothelial cells (CD31^+^) isolated from EAT mice after treatment with PBS, WD6305, or WD6305@TDF. WD6305@TDF selectively reduced METTL3 protein levels in TFCs with minimal effects on immune and endothelial cells, whereas free WD6305 reduced METTL3 without obvious cell selectivity. (D) Representative H&E, CD31, and CD45 staining of thyroid tissues showing that WD6305@TDF more effectively alleviated thyroid tissue injury, cell death, and immune infiltration than free WD6305. Scale bars, 100 µm. (E) Quantification of inflammatory scores in thyroid tissues from EAT mice treated with PBS, TDF, WD6305, or WD6305@TDF. (F–H) Flow cytometric quantification showing that WD6305@TDF restored immune balance in both thyroid tissues and peripheral blood, as reflected by decreased Th17 cell proportions (F), increased Treg cell proportions (G), and reduced Th17/Treg ratios (H). (I, J) ELISA analysis of VEGFA, IL‐6, and IL‐17A levels in serum (I) and thyroid tissue lysates (J). (K) ELISA analysis of serum TgAb and TPOAb levels in EAT mice. (L) Body weight curves recorded from day 0 to day 56, data are presented as Mean ± 95% CI. (M) Serum biochemical analyses showing no obvious adverse effects of WD6305@TDF on liver or kidney function, supporting its in vivo safety. **Data information**: Data are presented as mean ± SD or as appropriate. Statistical significance was determined using one‐way ANOVA followed by Tukey's multiple‐comparisons test for comparisons among multiple groups and two‐way ANOVA for longitudinal body weight analysis, where applicable. ns, not significant; *p < 0.05; **p < 0.01; ***p < 0.001; ****p < 0.0001.

Conventional METTL3 inhibitors may be limited by competition from the high intracellular SAM level [23]. Therefore, we turned to a PROTAC‐based strategy to induce METTL3 degradation through the ubiquitin‐proteasome pathway. Given that naked PROTACs often exhibit poor membrane permeability and insufficient lesion selectivity, we employed our previously reported Tg‐targeted DNA nanoflower (TDF) [24] to encapsulate the METTL3 degrader WD6305 [25], yielding WD6305@TDF. In this system, TDF mediates the selective delivery of WD6305 into Tg‐enriched thyroid cells, whereas WD6305 promotes METTL3 degradation by recruiting an E3 ubiquitin ligase (Figure 7A).

Flow cytometry showed that Cy5‐labeled TDF was preferentially taken up by Tg‐expressing TFCs (FRTL‐5 and Nthy‐ori 3‐1) and thyroid cancer cells (TPC1 and K1), but not by Tg‐negative cells, such as HEK293T or MCF7 (Figure S20A,B). Confocal imaging with LysoTracker further confirmed efficient endolysosomal escape of TDF after internalization (Figure S20C). Functionally, both WD6305 and WD6305@TDF induced dose‐dependent METTL3 degradation in TFCs after 24 h of treatment, reducing METTL3 levels by more than 60% at 0.4 µM, whereas no obvious degradation was detected in Tg‐negative cells (Figure 7B). These findings demonstrate that TDF encapsulation does not compromise the activity of WD6305 and instead enables Tg‐dependent cellular selectivity.

To assess the in vivo efficacy of WD6305@TDF, EAT mice were treated with WD6305, WD6305@TDF, TDF alone, or PBS. Analysis of sorted TFCs, immune cells, and endothelial cells showed that WD6305@TDF selectively depleted METTL3 in TFCs with minimal effects on immune cells, whereas free WD6305 reduced METTL3 expression non‐selectively (Figure 7C). Functionally, WD6305@TDF outperformed free WD6305 in alleviating thyroid injury, reducing immune infiltration, restoring vascular homeostasis (Figure 7D,E), correcting the Th17/Treg imbalance in both thyroid tissues and peripheral blood (Figure 7F–H; Figure S21), and lowering VEGF‐A and inflammatory cytokine levels in the thyroid and serum (Figure 7I,J). By contrast, TDF alone exhibited no detectable therapeutic benefit compared with PBS, indicating that the enhanced efficacy of WD6305@TDF is attributable to the combined effects of thyroid‐targeted delivery and METTL3 PROTAC‐mediated degradation. Importantly, WD6305@TDF also reduced autoantibody levels without affecting body weight or hepatic and renal function (Figure 7K–M), supporting its in vivo safety. Together, these results demonstrate that thyroid‐targeted METTL3 degradation by WD6305@TDF effectively reverses persistent autoimmune inflammation and mitigates thyroid tissue damage.

## 3 Discussion

AIT is a chronic autoimmune disease with a multifactorial and incompletely understood pathogenesis. In this study, we identified a previously unrecognized role of METTL3 in promoting thyroid vascular remodeling and inflammation in AIT. We revealed that METTL3‐dependent epitranscriptomic regulation enhances KDR expression and strengthens the VEGFA‐KDR positive feedback loop, thereby amplifying vascular activation and inflammatory responses that contribute to AIT pathogenesis. Targeting METTL3 or VEGFA‐KDR signaling represents a promising therapeutic strategy to halt AIT progression.

Clinical observations have demonstrated that patients with subclinical hypothyroidism and active AIT exhibit significantly elevated total oxidative status (TOS) and oxidative stress index (OSI) [26], suggesting that oxidative stress is a critical risk factor for disease progression. Previous studies have shown that oxidative stress suppresses SIRT1 in AIT [27]. Several non‐exclusive mechanisms may explain how ROS suppresses SIRT1. In our study, oxidative stress and AIT‐related inflammatory stimulation consistently reduced SIRT1 expression, which was accompanied by increased H3K27ac enrichment at the METTL3 promoter and increased METTL3 transcription. A plausible interpretation is that loss of SIRT1 weakens promoter deacetylation, increases chromatin accessibility [28], and thereby facilitates METTL3 upregulation. Although ChIP‐qPCR did not detect robust SIRT1 occupancy at the METTL3 promoter, this does not exclude a functional role, as SIRT1 is more likely to act as a transiently recruited stress‐responsive deacetylase than as a stable DNA‐binding factor. It is also possible that SIRT1 indirectly regulates METTL3 transcription through deacetylation of non‐histone substrates, such as YY1 or FOXO family transcription factors. Our rescue experiments further support this mechanism. Re‐expression of wild‐type SIRT1, but not the catalytically inactive H363Y mutant, reduced H3K27ac enrichment at the METTL3 promoter and suppressed METTL3 expression in SIRT1‐deficient cells, indicating that SIRT1 restrains METTL3 transcription through its deacetylase activity. More broadly, the epigenetic control of METTL3 is unlikely to be governed by a single modification. Previous studies in cancer have implicated H3K27ac, H3K4me3, and multiple transcription factors in METTL3 promoter regulation. A more complete understanding of the epigenetic landscape governing METTL3 in inflammatory diseases, including AIT, will therefore be an important subject for future study.

m^6^A modification is the most common modification in human mRNA and plays a crucial role in the pathophysiology of many diseases [29]. Recent evidence has established m6A modification as a key regulator of immune and inflammatory responses in autoimmune diseases. METTL3 has been implicated in controlling macrophage polarization, T‐cell differentiation, and cytokine production in rheumatoid arthritis, primary Sjögren's syndrome, and psoriasis [30, 31]. However, few studies have focused on the role of m^6^A modification in thyroid diseases. Here, we show that AIT‐related stimulation consistently increased METTL3 expression in cellular models, mouse models, and clinical AIT samples. Using a 3D TFC‐immune co‐culture system to mimic the inflammatory thyroid microenvironment, we found that METTL3 silencing in TFCs attenuated AIT‐mix‐induced M1 polarization, corrected Th17/Treg skewing, and reduced IL‐6 and IL‐17A production. These findings support an active role for TFC‐intrinsic METTL3 in shaping the immune‐inflammatory circuitry of AIT.

To further test this concept in vivo, we established AAV‐mediated TFC‐targeted METTL3 knockdown mice. Although flow‐based analyses indicated that the knockdown was largely restricted to TFCs rather than CD45^+^ or CD31^+^ cells, we cannot fully exclude off‐target effects associated with systemic AAV8 delivery. In addition, we did not generate a TFC‐specific conditional knockout model, mainly because of time, cost, and technical constraints. Future studies using cell type‐specific Cre‐loxP systems will therefore be needed to more rigorously define the contribution of METTL3 in different thyroid‐resident and infiltrating cell populations during AIT progression.

To further explore the downstream mechanism of METTL3, we used the m^6^A‐mRNA & lncRNA Epitranscriptomic Microarray and revealed that METTL3 silencing decreased the m^6^A abundance and stability of KDR mRNA. The fate of target transcripts typically depends on their specific recognition by m^6^A readers [28, 32]. In addition, the DRACH consensus sequence harboring the KDR m^6^A site was found to be evolutionarily conserved across species. It should be noted, however, that the microarray also identified several additional candidate targets of METTL3. Among them, KDR showed the strongest and most consistent response to both METTL3 depletion and AIT stimulation, which is why it was prioritized in the present work. Still, other candidates, including GDF15 and FZD1, may also contribute to disease progression. Future studies will be needed to determine whether these transcripts function independently, cooperatively, or in parallel with KDR, and to build a more comprehensive METTL3‐centered regulatory network in the context of thyroiditis.

Angiogenesis and increased vascular permeability are major drivers of immune dysregulation and chronic tissue damage. Pseudo‐bulk analysis of vascular regions from spatial transcriptomic data of AITD and control thyroid tissues revealed that genes associated with angiogenesis and endothelial permeability were most highly expressed in AIT, surpassing both Graves’ disease and normal controls [33, 34]. KDR, a receptor tyrosine kinase on the cell membrane, is most prominently expressed in endothelial cells [22]. Accumulating evidence indicates that KDR is not restricted to endothelial cells but can also function in diverse non‐endothelial cell types, thereby extending its role beyond angiogenesis to the broader regulation of tissue homeostasis and disease progression. In several solid tumors, aberrant KDR expression in tumor cells sustains VEGFA‐dependent autocrine/paracrine signaling, promoting cell survival, proliferation, stemness, migration, and invasion. Similar noncanonical functions have also been reported in inflammatory disorders, including atopic dermatitis, sepsis, rheumatoid arthritis, and inflammatory bowel disease, where KDR signaling contributes to inflammatory amplification, angiogenesis, and tissue injury [35, 36]. In thyroid disease, KDR is specifically upregulated in goiter, and the VEGFA/KDR axis in TFCs has been identified as a key regulator of TSH‐dependent follicular remodeling. Together, these observations support the concept that pathological KDR induction in non‐endothelial cells may represent an active driver, rather than a passive bystander, in disease progression. Notably, prior studies have also linked METTL3 or SIRT1 to the regulation of VEGFA expression. METTL3 has been shown to enhance VEGFA expression in multiple cellular contexts, whereas reduced SIRT1 activity has been associated with increased VEGFA production and immune dysregulation in autoimmune diseases [27]. In our study, AIT‐related stimulation, including AIT‐mix and patient‐ or model‐derived serum, consistently induced METTL3 and KDR upregulation in TFCs, together with activation of the KDR‐VEGFA signaling axis. To reinforce the translational relevance of this finding, we combined expanded clinical tissue validation, single‐cell transcriptomic analysis, and freshly isolated TFC‐based expression assays, which collectively established aberrant activation of the METTL3‐KDR axis in AIT. Moreover, using overexpression and knockdown approaches, m6A‐site mutagenesis, 3D cell models, and thyroid‐targeted in vivo genetic manipulation, we demonstrated that this axis functionally promotes inflammatory amplification and tissue damage. These findings position the METTL3‐KDR pathway as a pathogenic signaling module in AIT.

Targeted protein degradation (TPD) is an emerging strategy for eliminating proteins that are otherwise difficult to inhibit effectively. Conventional TPD approaches, however, are often limited by inefficient delivery and poor selectivity for intracellular targets [37]. By integrating a METTL3 PROTAC with our previously developed Tg‐targeted DNA nanoflower platform, we achieved thyroid‐selective METTL3 degradation in vivo. Compared with the free parental compound WD6305, this targeted delivery system exhibited superior efficacy and safety in suppressing persistent inflammation and mitigating thyroid tissue injury in AIT. Overall, our findings establish a mechanistic and translational basis for therapeutic targeting of the METTL3‐KDR axis in autoimmune thyroid disease.

Despite these findings, several limitations should be considered. First, although our data support a role for SIRT1 in regulating METTL3 promoter acetylation, we were unable to detect clear SIRT1 occupancy at the promoter, raising the possibility that this regulation is indirect or mediated through transient chromatin recruitment that is difficult to capture experimentally. Second, while AAV8‐based TFC‐directed knockdown and overexpression showed a degree of specificity by co‐staining analysis, more definitive validation will require conditional cell type‐specific genetic models. Third, although we observed activation of the METTL3‐KDR axis in clinical AIT samples, the cohort size was limited. Larger studies will therefore be necessary to clarify whether this pathway is associated with AIT progression, prognosis, and its potential utility as a diagnostic biomarker.

## 4 Materials and Methods

For more information on the materials and methods, including statistical analysis, please refer to the supplementary materials. Chemical and biological reagents (Table S4), antibodies (Table S5), shRNA sequences (Table S6), primers sequences (Tables S7–S9).

## Author Contributions

**Q.H**., **J.M**., and **T.H**. conceived and designed the study. **Q.H**. implemented the experimental design. **Q.H**., **H.L**., **A.R**., and **Z.T**. performed the experiments. **Q.H**. and **H.L**. were responsible for cell isolation, culture, and animal modeling. **Q.H**., **W.Y**., and **J.M**. collected the clinical tissue samples. **Q.H**. analyzed the data and drafted the manuscript. **Q.H**., **J.M**., and **T.H**. revised the paper. All authors read and approved the final manuscript.

## Funding

National Natural Science Foundation of China Grant (82403712), China Postdoctoral Science Foundation (2024M751029), National Natural Science Foundation of China Grant (82270830), (82573420), and Key Program of Natural Science Foundation of Hubei Province (2021BCA142).

## Ethics Statement

All animal experiments in this study were conducted in accordance with the Guidelines of Huazhong University of Science and Technology and were approved by the Institutional Animal Care and Use Committee (IACUC) of HUST (Approval No. 4558). Human thyroid paraffin‐embedded sample were obtained from the Union Hospital, Huazhong University of Science and Technology, and the study was conducted following approval by the institutional ethics committee (Approval No.1413). All samples were anonymized prior to analysis.

## Conflicts of Interest

The authors declare no conflicts of interest.

## Supporting information




**Supporting File**: advs75762‐sup‐0001‐SuppMat.docx.

## Data Availability

The data that support the findings of this study are available from the corresponding author upon reasonable request.
