# Supplementary material for: Targeting METTL3 Attenuates Thyroid Inflammatory Injury by Restoring Th17/Treg Balance through a YTHDC2‐m6A‐Dependent KDR/VEGFA Loop
Source: Adv Sci (Weinh). 2026 Jun 23:e22453. Online ahead of print. doi: 10.1002/advs.202522453 (PMC13335789; doi:10.1002/advs.202522453)
Supplement: Supplementary file 1 — Supporting File: advs75762‐sup‐0001‐SuppMat.docx. [file ADVS-9999-e22453-s001.docx]

Supporting Information

Targeting METTL3 Attenuates Thyroid Inflammatory Injury by Restoring Th17/Treg Balance Through a YTHDC2-m6A-Dependent KDR/VEGFA Loop

Qingyi Hu, Huan Liu, Anwen Ren, Zimei Tang, Jie Tan, Wen Yang, Jie Ming*, Tao Huang*

**Table of contents:**

Materials and methods

Supplementary Tables: Table S1-9

Supplementary Figures: Figure S1-21

1. **Materials and Methods**

**Patient enrollment and diagnostic criteria**

A total of 34 patients with autoimmune thyroiditis (AIT) who were treated at our center were enrolled in this study. The control group consisted of five thyroid tissue samples obtained from subjects with thyroid function parameters and thyroid autoantibodies within the normal reference ranges and without histopathological evidence of chronic inflammatory changes. Written informed consent was obtained from all participants, and the study was approved by the institutional ethics committee. The diagnosis of AIT was established based on an integrated evaluation of thyroid autoantibodies, thyroid function, and thyroid ultrasonographic findings. AIT was defined as elevated serum TPOAb and/or TgAb levels above the institutional upper reference limit, together with ultrasonographic features suggestive of chronic thyroiditis, including diffuse hypoechogenicity, heterogeneous echotexture, or parenchymal heterogeneity.

The inclusion criteria for AIT patients were as follows: age ≥18 years; fulfillment of the diagnostic criteria for AIT; availability of complete clinical data, including thyroid function parameters ^[1]^, thyroid autoantibodies (TPOAb and TgAb), and thyroid ultrasonography results; and provision of written informed consent.

The exclusion criteria were as follows: treatment with glucocorticoids, immunosuppressants, or immunomodulatory agents within the previous 6 months; current use of medications that may affect thyroid function or immune status; levothyroxine or antithyroid drug treatment for less than 3 months or unstable dose adjustment within the previous 3 months; coexistence of other organ-specific or systemic autoimmune diseases; acute infection within 4 weeks or chronic infection; thyroid nodules classified as TI-RADS category 4 or higher or suspected thyroid malignancy; a history of thyroid cancer or other cervical malignancies; pregnancy or lactation; severe cardiac, hepatic, renal, or hematologic disorders; smoking, alcohol abuse, or substance abuse; prior thyroid surgery, radioiodine therapy, or cervical radiotherapy; and incomplete clinical data precluding a definite diagnosis.

Control tissues were included only when the corresponding subjects had thyroid function parameters within the normal reference ranges, TPOAb and TgAb levels within the normal reference ranges, and no histopathological evidence of chronic inflammatory changes.

**Experimental autoimmune thyroiditis model**

Wistar rats and C57BL/6 mice were purchased from GemPharmatech (Jiangsu, China) and maintained unsder specific pathogen-free (SPF) conditions. The animals were fed the same standard mouse diet and water for one-week adaptation before the experiment. The experimental autoimmune thyroiditis (EAT) model used in this study was established based on a classical and widely adopted murine EAT induction protocol ^[2, 3]^, with further optimization through our laboratory’s preliminary investigations. The detailed modeling procedure was as follows: 8-week-old female C57BL/6J mice were provided with potassium iodide (KI)-supplemented drinking water (500 mg/L) for six consecutive weeks. Beginning in the second week, mice were immunized subcutaneously at multiple sites, including the neck, bilateral groins, and footpads, with 200 μg bovine thyroglobulin (bTg) emulsified in complete Freund’s adjuvant. Three hours after the first immunization, mice received a tail-vein injection of 20 μg lipopolysaccharide (LPS) dissolved in 100 μL phosphate-buffered saline (PBS) to potentiate the autoimmune response. During weeks 3-6, mice received weekly subcutaneous booster immunizations using 200 μg bTg emulsified in incomplete Freund’s adjuvant and weekly tail-vein injections of 20 μg LPS in PBS. Control mice were injected at identical sites with an equal volume of PBS. For mechanistic studies, mice received tail-vein injections every 2 weeks (1 × 10^9^ PFU per mouse) of AAV-TPO-Mettl3 shRNA, AAV-TPO-KDR, or a combination of both. Recombinant adenoviruses were purchased from GeneChem (Shanghai, China). For sunitinib treatment, EAT model mice were additionally administered sunitinib (80 mg/kg) by intraperitoneal injection every other day for 2 weeks. At the end of the experimental protocol, all animals were euthanized, and peripheral blood and thyroid tissues were collected for subsequent analyses. Because Tg-driven autoimmune injury disrupts thyroid follicles and exposes additional thyroid autoantigens, such as TPO, it can trigger secondary autoantibody responses against non-priming antigens. Therefore, increased serum TgAb and/or TPOAb levels were used as serological indicators of successful EAT induction.

**Primary cell isolation**

Thyroid tissues were finely minced using ophthalmic scissors and digested in 1 mL of enzyme solution containing collagenase IV (1 mg/mL) and DNase I (0.1 mg/mL) at 37 °C in a 5% CO_2_ incubator for 30-40 min, with gentle pipetting every few minutes. Digestion was terminated by adding 2 mL of RPMI-1640 medium supplemented with 10% FBS. The cell suspension was passed through a 70 μm cell strainer to remove undigested debris and centrifuged at 1,500 rpm for 5 min at 4 °C. The supernatant was discarded, and the cell pellet was resuspended in flow cytometry staining buffer, counted, and adjusted to a final concentration of 1 × 10^6^ - 1 × 10^7^ cells/mL.

For PBMCs isolation, cells were isolated by density-gradient centrifugation according to the manufacturer’s standard protocol. After centrifugation, the mononuclear cell layer was carefully collected, washed with RPMI-1640 medium, and centrifuged at 1,500 rpm for 5 min at 4 °C. The supernatant was discarded, and the cells were resuspended in staining buffer to prepare a single-cell suspension for downstream experiments.

Fresh spleens were mechanically dissociated by gently grinding the tissues through a 70 μm cell strainer using the plunger end of a sterile syringe in cold RPMI-1640 medium. The resulting cell suspension was collected and centrifuged at 1500 rpm for 5 min at 4 °C. After removal of the supernatant, the cell pellet was treated with red blood cell lysis buffer for 1-3 min at room temperature to remove erythrocytes. Lysis was terminated by adding excess RPMI-1640 medium containing 10% FBS, followed by centrifugation at 1,500 rpm for 5 min at 4 °C. The final cell pellet was resuspended in staining buffer, filtered if necessary, counted, and adjusted to the appropriate concentration for subsequent assays.

**Histological examination**

After total thyroidectomy, mouse thyroid tissues were fixed in 4% phosphate-buffered paraformaldehyde (PFA), embedded in paraffin, and sectioned at 4 μm. The sections were stained with hematoxylin and eosin (H&E) for routine histopathological evaluation. The degree of lymphocytic infiltration was assessed under a light microscope and graded according to the following criteria ^[4]^: 0 = normal thyroid; 1 = thyroid lymphocytic infiltration < 1%; 2 = thyroid lymphocytic infiltration 1-20%; 3 = thyroid lymphocytic infiltration 20-50%; and 4 = thyroid lymphocytic infiltration >50%. Immunofluorescence and immunohistochemistry (IHC) were performed as described previously. The antibodies used are listed in the Supplementary Materials, with the following dilutions: anti-METTL3 (1:50), anti-SIRT1 (1:50), anti-Tg (1:200), anti-4-HNE (1:400), anti-CD45 (1:100), anti-CD31 (1:100), anti-KDR (1:100), and anti-Ki-67 (1:100). At least five carefully selected fields of view from stained sections were imaged and examined at 200× magnification. The proportion of positive areas was calculated using ImageJ.

**Measurement of thyroid related antibodies, hormones and cytokines**

Serum concentrations of thyroglobulin antibody (TgAb), thyroid peroxidase antibody (TPOAb) were determined using commercially available mouse ELISA kits according to the manufacturers’ instructions. Levels of pro- and anti-inflammatory cytokines, including interferon-γ (IFN-γ), interleukin-6 (IL-6), interleukin-4 (IL-4), interleukin-17A (IL-17A), and tumor necrosis factor-α (TNF-α), were quantified by ELISA in thyroid tissue homogenates, serum samples, and cell culture supernatants. All cytokine ELISA kits were listed in Supplementary Materials, and assays were performed strictly following the manufacturers’ protocols.

**RNA m^6^A quantitative measurement**

According to the manufacturer’s protocol, m^6^A levels were quantitatively analyzed using the EpiQuik m^6^A RNA Methylation Quantification Kit. The absorbance at 450 nm was measured using a microplate reader. The m^6^A amount was calculated using the following formula:

$$\text{m}\text{6}\text{A}\text{ \%=}\frac{\left( \text{Sample}\text{ }\text{OD}\text{-}\text{NC}\text{ }\text{OD} \right)\text{÷}\text{S}}{\left( \text{PC}\text{ }\text{OD}\text{-}\text{NC}\text{ }\text{OD} \right)\text{÷}\text{P}}\text{ × 100 \%}$$

S is the amount of sample RNA input (ng), and p is the amount of positive control (PC) input (ng).

**Dot blot assay**

RNA was extracted and quantified using a Nanodrop 2000 spectrophotometer (ThermoFisher) to determine the RNA concentration. Equal amounts of RNA were loaded onto a nitrocellulose membrane (NC membrane; MerckMillipore). The membrane was air-dried and crosslinked under UV light at 3 J/cm² for 10 min. After washing with PBST, the membrane was stained with 0.3 M sodium acetate (pH 5.2) containing 0.02% methylene blue to visualize total RNA content. The membrane was blocked with PBST containing 5% non-fat dry milk for 1 h. A rabbit anti-m^6^A antibody diluted 1:1000 in PBST was added to the membrane and incubated overnight at 4 °C. After washing with PBST, the membrane was incubated with a secondary antibody diluted 1:5000 in blocking buffer at room temperature for 2 hours. RNA m^6^A levels were visualized using ECL detection solution. Equal amounts of RNA were spotted onto the membrane, and methylene blue staining was used as a loading control.

**Cell culture**

Rat thyroid follicular FRTL-5 cells were cultured in Coon’s modified F-12 medium (Gibco) containing 1 mU/mL TSH and 10 μg /mL insulin. Human normal thyroid cells (Nthy-ori 3-1, Nthy) were cultured in RPMI-1640 medium (BI). HUVEC were cultured in Dulbecco's modified Eagle medium (DMEM, BI). The culture medium was supplemented with 10% fetal bovine serum (FBS) (Gibco), and penicillin-streptomycin-amphotericin B solution (1:100, Beyotime). All cell lines were cultured in a humidified atmosphere at 37℃ with 5% CO_2_.

**In Vitro 3D Co-Culture System**

Peripheral blood was collected from healthy donors and Wistar rats. The obtained PBMCs were resuspended in RPMI-1640 complete medium supplemented with 10% fetal bovine serum (FBS). For T-cell activation, PBMCs were pre-stimulated with anti-CD3/CD28 monoclonal antibodies (1 μg/mL) for 24 h before co-culture. For macrophage induction, PBMC-derived monocytes were treated with M-CSF (20 ng/mL) for 5 days to obtain resting M0 macrophages. The immune cells were then seeded into the upper chamber of the Transwell system. For thyroid follicular cells (TFCs) spheroid formation, Nthy-ori 3-1 and FRTL-5 cells were cultured in a rotary culture system and transferred into U-bottom 96-well plates to generate multicellular spheroids. After stimulation with thyroid-stimulating hormone (TSH, 1.0 mU/mL) for 5 days, the spheroids were exposed to AIT-mix, consisting of lipopolysaccharide (LPS, 1 μg/mL) and IFN-γ (4 IU/mL), for 24 h. Subsequently, thyroid-derived DNA/RNA damage-associated molecular patterns (DAMPs) and bovine thyroglobulin (bTg) peptide antigens (10 μg/mL) were added to mimic autoantigen exposure. Activated TFCs spheroids were then co-cultured with the corresponding species-matched immune cells in 0.4 μm Transwell inserts for 48 h, allowing paracrine communication while preventing direct cell-cell contact. All co-culture experiments were conducted in a species-matched manner.

**Plasmid constructs and cell transduction**

For the construction of overexpression cell lines, protein expression plasmids containing the open reading frames (ORFs) of METTL3 and SIRT1 were purchased from MiaoLing Bio (Table S4). The SIRT1 H363Y ^[5, 6]^ mutation was based on previous studies. To generate the mutant METTL3 D395A construct, the cDNA fragment was amplified and inserted into the pcDNA3.1 vector. Transfer plasmids and packaging vectors (pMD2.G and psPAX2) were transfected into HEK293T cells at 40% confluence using polyethyleneimine (PEI) . After 48 and 72 h of transfection, the culture medium containing lentiviral particles was collected and used to infect target cells, with 8 μg/ml polybrene added to enhance infection efficiency. To stably knock down the target gene, we used lentiviral vectors encoding short hairpin RNA (shRNA) targeting the gene of interest or scrambled shRNA (shNC). Cells were seeded into 6-well plates and cultured to 40% confluence. Infection was carried out at a multiplicity of infection (MOI) ranging from 20 to 200 in the presence of 4 μg/ml polybrene to generate target gene knockdown and control cells. The shRNA target sequences were shown in Table S4. Stable cells were selected by treatment with 2.5 μg/ml puromycin (Selleck) for 2 weeks. Stable cell lines were confirmed by RT-qPCR and Western blot analysis.

**RNA isolation and quantitative reverse transcription PCR (RT-qPCR)**

Total RNA was extracted using TRIzol reagent according to the manufacturer's instructions, and 1 μg of total RNA was reverse transcribed into cDNA. RT-qPCR was performed using SYBR Green (Takara) on the CFX96 Real-Time RT-qPCR Detection System (Bio-Rad) to measure the expression levels of target genes. The relative genes expression levels were calculated using the 2 ^−ΔΔCt^ method and normalized to GAPDH. The primer sequences used for RT-qPCR in this study are listed in Table S7, and all DNA primers were synthesized by Sangon Biotech (Shanghai, China).

**Western blot**

Cells were lysed using Western/IP lysis buffer and subjected to sonication. After centrifugation, the protein concentration in the lysates was quantified using the bicinchoninic acid (BCA) assay kit. 30 μg of protein were separated by 10% SDS-PAGE and transferred onto a 0.4 μm NC membrane. After blocking with 5% non-fat milk solution for approximately 2 hours at room temperature, the membrane was incubated overnight with specific primary antibodies at 4°C. The membrane was then incubated with HRP-conjugated secondary antibodies at room temperature for 1 hour. Target protein levels were detected using ECL detection reagent. The antibodies used in this study are listed in Table S3.

**Cell viability assay**

Cells were seeded in 96-well plates at a density of 8000 cells/well and cultured overnight to allow adherence. After treatment with different formulations, cell viability was assessed using the Cell Counting Kit-8 (CCK-8) assay. Briefly, 10 μL of CCK-8 reagent and 90 μL of RPMI-1640 medium were mixed and added to each well, followed by incubation for 1 h at 37 °C in a 5% CO₂ incubator. Absorbance at 450 nm was measured using a microplate reader (ThermoFisher Scientific).

**Single-Cell RNA Sequencing Analysis**

After euthanasia, mouse thyroid tissues were rapidly isolated under sterile conditions, transferred into pre-chilled PBS, and carefully stripped of surrounding fat and connective tissue. The tissues were then minced into approximately 1 mm3 pieces and placed in MACS® Tissue Storage Solution (Miltenyi). Single-cell RNA sequencing and data analysis were conducted by Seqhealth Technology Co., LTD (Wuhan, China). Downstream analyses were conducted in R using the Seurat package. Briefly, low-quality cells and potential doublets were removed during quality control. The filtered data were then normalized, and highly variable genes were identified. The expression matrix was subsequently scaled and centered, with potential confounding factors, including UMI counts, mitochondrial gene proportion, and cell-cycle effects, regressed out when necessary. Principal component analysis (PCA) was then performed to capture the major sources of transcriptional variation. To minimize batch effects across samples, datasets were integrated using the Harmony algorithm. Unsupervised clustering was conducted using FindNeighbors and FindClusters, and the clustered cells were visualized by uniform manifold approximation and projection (UMAP). Cell identities were assigned based on the expression of canonical marker genes in each cluster. For selected cell populations, including TFCs and T cells, differential expression analysis was performed between the EAT and control groups to identify DEGs, using the thresholds of adjusted P value < 0.05 and |log2 fold change| > 0.5. Pseudotime trajectory analysis was further performed on TFCs to investigate cell-state transitions and differentiation dynamics under disease conditions. To further characterize intercellular communication within the EAT microenvironment, CellChat analysis was applied to infer cell-cell interaction networks, with particular emphasis on ligand-receptor interactions between immune cells and TFCs.

**Bulk RNA-seq**

After completion of animal modeling, mice were anesthetized with isoflurane inhalation and euthanized by cervical dislocation. Thyroid glands were rapidly dissected, rinsed with ice-cold enzyme-free PBS to remove residual blood, blotted dry on sterile filter paper, and immediately transferred into 1 mL of pre-chilled TRIzol reagent for total RNA extraction. For each sample, 30-50 mg of thyroid tissue was used. UID RNA-seq experiments, high-throughput sequencing, and data analysis were conducted by Seqhealth Technology Co., Ltd (Wuhan, China). Differentially expressed genes (DEGs) were identified using the DESeq2 package (v1.34.0) in R. Genes with |log_2_(fold change)| > 1.0 and adjusted P value (Padj) < 0.05 were considered significantly differentially expressed. Functional enrichment analyses, including Gene Ontology (GO) and Kyoto Encyclopedia of Genes and Genomes (KEGG) pathway analyses, were conducted using the clusterProfiler R package (v4.2.2). Gene set enrichment analysis (GSEA) was performed using hallmark and curated gene sets retrieved from the Molecular Signatures Database (MSigDB), and gene sets relevant to the research focus were selected for visualization of enrichment plots.

**Flow cytometry analysis and gating strategy**

An aliquot of 100 μL of single-cell suspension containing approximately 1 × 10^6^ cells was transferred into each flow tube, including sample tubes, single-stain controls, and blank controls. Fluorescently labeled antibodies listed in Supplementary Table S5 were added, and samples were incubated at 4 °C for 30 min in the dark. After washing, cells were resuspended in staining buffer and kept on ice in the dark until acquisition using a Sony 700 flow cytometer. Data were analyzed with FlowJo software following the gating strategy described below.


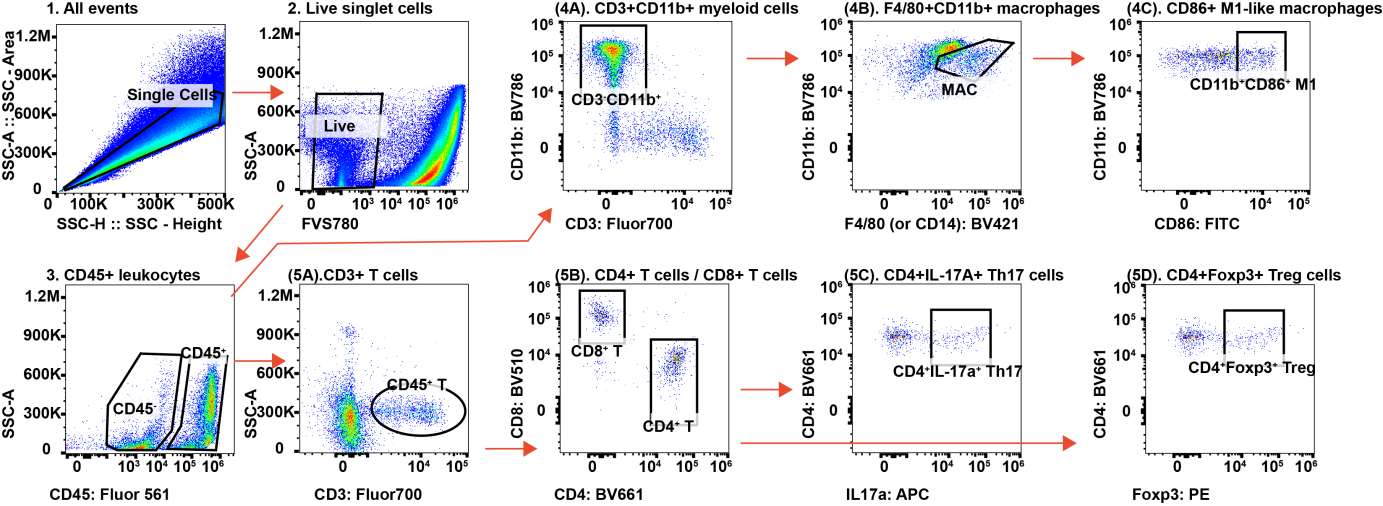


**ChIP assay**

ChIP-qPCR was performed using the EZ-ChIP Kit (Magnetic Beads; Sigma-Aldrich) according to the manufacturer's instructions. Briefly, 5 × 10^6^ cells were crosslinked with 1% formaldehyde at 37 °C for 10 min. The cell lysates were sonicated to fragment the chromatin. After sonication, DNA-protein complexes were immunoprecipitated overnight using the following specific antibodies: anti-histone H3 acetyl K27 antibody - ChIP grade, anti-histone H3 tri methyl K4 antibody - ChIP Grade, with IgG antibody as a negative control. DNA fragments were purified using a DNA purification kit, and DNA content was detected by RT-qPCR. The primer sequences for the METTL3 promoter region used in the ChIP-qPCR are listed in Table S5.

**Human m^6^A -mRNA&lncRNA Epitranscriptomic microarray analysis**

Arraystar Human m^6^A -mRNA&lncRNA epitranscriptomic microarray analysis was supported by Aksomics. Briefly, Nthy-ori 3-1 cells were transfected with M3sh and shNC in three independent wells, then total RNA was extracted. The purity and concentration of total RNA samples were quantified using the NanoDrop 2000 spectrophotometer. RNA was fragmented and purified. Protein A/G magnetic beads were used for co-immunoprecipitation with m^6^A antibody. The immunoprecipitated “IP” fraction contained enriched m^6^A -methylated RNA, whereas the supernatant “SUP” fraction contains unmethylated RNA. Using the Arraystar Super RNA labeling kit, the “IP” and “SUP” labeled RNA were amplified into cRNA and labeled with Cy5 and Cy3, respectively. The Cy3 and Cy5 labeled cRNA were combined and hybridized with the Arraystar Human mRNA and lncRNA transcriptomic array (8 × 60K, Arraystar) in an Agilent hybridization oven at 65°C for 17 h. After washing, the array was scanned using an Agilent Scanner G2505C. Data extraction was performed using Agilent Feature Extraction software. Differentially m6A-methylated RNAs were subjected to clustering heatmap analysis. Differentially m6A-methylated mRNAs were further analyzed using GO and pathway enrichment analyses.

**RIP-qPCR and MeRIP-qPCR**

Following the manufacturer's instructions, RIP was performed using the Magna RIP RNA-binding protein immunoprecipitation kit (Millipore, Bedford, MA, USA), and MeRIP was performed using the Magna MeRIP™ m^6^A kit (Millipore, Bedford, MA, USA). For RIP, a total of 20 μg RNA samples were incubated with 1 μg of anti-YTHDC2 antibody, anti-METTL3 antibody, or IgG (negative control). For MeRIP, 20 μg of total RNA samples were incubated with 1 μg of anti-m^6^A antibody conjugated to magnetic beads at 4 °C for m^6^A immunoprecipitation. The immunoprecipitated RNA was purified and analyzed by RT-qPCR. PCR amplification of GAPDH was used as a negative control. The primers used for RIP-qPCR and MeRIP-qPCR are listed in Table S6.

**ROS Detection**

Intracellular ROS levels were assessed using the Reactive Oxygen Species Assay Kit according to the manufacturer's instructions.

**Immunofluorescence (IF)**

Cells were seeded on a glass coverslip in 24-well plate and fixed with 4% paraformaldehyde for 30 min. After washing with PBS, the slides were blocked with 10% goat serum and permeabilized with PBST (PBS containing 0.1% Triton X-100), followed by incubation with the primary antibody and then the fluorescently labeled secondary antibody. Antifade Mounting Medium (Beyotime, P0126) was applied to the slides, which were then covered and observed under a fluorescence microscope.

**RNA and protein stability assays**

To measure the RNA decay rate of KDR mRNA, 5 μg/mL of actinomycin D was added to the cell culture medium to block de novo RNA synthesis. Cell samples were collected at designated time points, RNA was extracted, and detected by real-time PCR. To measure the protein decay rate, cells were treated with 100 μg/mL cycloheximide (CHX), and protein was extracted at designated time points for Western blot analysis.

**Measurement of VEGFA secretion**

Equal numbers of cells (1×10^5 cells/well) were seeded in a 6-well plates and serum-starved for 24 h. The cells were then treated with 30 ng/mL recombinant human VEGFA in serum-free medium containing 10 μM sunitinib or vehicle for 2 h. After washed with PBS, the cells were incubated in fresh serum-free medium for 24 h. The supernatant was collected and VEGFA secretion was measured using a human VEGFA ELISA kit according to the manufacturer's instructions.

**Conditioned medium culture and HUVEC tube formation assay**

Stably transfected cells were cultured in complete medium to 80% confluence. After washing with PBS, the cells were treated with rhVEGFA and/or sunitinib as described above. After 24 h of serum starvation, the conditioned medium was collected and filtered through a 0.22 µm filter (Millipore). A 96-well plate was pre-coated with 60 µL/well growth factor-reduced Matrigel (BD Biosciences). HUVECs were serum-starved for 3-6 h, then resuspended in conditioned medium containing 1% FBS and added to the 96-well plate (3-5×10^4 cells/well). The plate was incubated at 37°C for 6 h, and capillary network formation was imaged under an optical microscope. The number of branch points in three random fields per well was calculated.

**Statistical analysis**

Data analysis and graph generation were performed using GraphPad Prism 10. All data are presented as mean ± SD. Statistical significance was determined using an unpaired two-tailed Student’s t-test for two-group comparisons or one-way ANOVA followed by Tukey’s multiple-comparisons test for comparisons among multiple groups. Correlations were analyzed using Spearman’s rank correlation test. Two-way ANOVA for time-course analyses were applicable. Statistical significance for all tests is indicated as follows: ns, not significant; *P < 0.05; **P < 0.01; ***P < 0.001; ****P < 0.0001.

1. **Supplementary Tables**

**Table S1. Inflammation scores of EAT model in each groups of mice.**

|  | Group |  | No.1 | No.2 | No.3 | No.4 | No.5 | Mean ± SD | P |
| --- | --- | --- | --- | --- | --- | --- | --- | --- | --- |
| Fig1 | CTRL |  | 0 | 0 | 0 | 0 | 0 | 0.00 ± 0.000 | *** |
|  | EAT |  | 4 | 3 | 2 | 1 | 3 | 2.60 ± 0.5099 |  |
| Fig S19 | PBS |  | 3 | 4 | 2 | 2 | 3 | 2.80 ± 0.3742 | * |
|  | Su |  | 2 | 1 | 2 | 1 | 2 | 1.60 ± 0.2499 |  |
| Fig 7 | PBS |  | 3 | 3 | 4 | 2 | 2 | 2.80 ± 0.3742 | * |
|  | TDF |  | 3 | 2 | 2 | 3 | 2 | 2.40 ± 0.2449 |  |
|  | WD6305 |  | 1 | 2 | 1 | 3 | 1 | 1.60 ± 0.4000 |  |
|  | WD6305@TDF |  | 1 | 2 | 1 | 1 | 2 | 1.40 ± 0.2449 |  |

Statistical significance was determined using unpaired two-tailed Student’s t-test for two-group comparisons (Fig1 and Fig S19) and one-way ANOVA for comparisons among multiple groups (Fig 7). ns, not significant; *P < 0.05; **P < 0.01; ***P < 0.001; ****P < 0.0001.

**Table S2. Demographic data and clinical parameters from patients with autoimmune thyroiditis (AIT).**

| Patients | Gender | Age (y) | TSH  (mUI/L) | FT3  (pmol/L) | FT4  (pmol/L) | TPO Ab (UI/mL) | Tg Ab (UI/mL) |
| --- | --- | --- | --- | --- | --- | --- | --- |
| A01 | F | 33 | 5.76 | 5.61 | 7.62 | 115.71 | 2120.70 |
| A02 | F | 52 | 1.96 | 5.41 | 10.45 | 137.82 | ＞1000 |
| A03 | F | 59 | 1.60 | ND | 13.31 | ＞1000 | 210.48 |
| A04 | F | 26 | 0.21 | 4.10 | 13.23 | 152.24 | 5.75 |
| A05 | F | 41 | 0.90 | 4.53 | 11.91 | 661.38 | ＞4000 |
| A06 | F | 49 | 0.82 | ND | 12.35 | 210.59 | 693.73 |
| A07 | F | 46 | 1.57 | 4.92 | 12.47 | 575.16 | 18.49 |
| A08 | F | 52 | 0.95 | ND | 12.21 | 189.76 | 300.75 |
| A09 | M | 49 | 2.00 | ND | 13.50 | ND | ND |
| A10 | F | 54 | 0.26 | 4.40 | 3.65 | 693.64 | 15.34 |
| A11 | F | 54 | 1.21 | 3.18 | 11.91 | 186.41 | 77.79 |
| A12 | F | 26 | 3.33 | ND | ND | 0.62 | 61.31 |
| A13 | F | 46 | 0.91 | 3.85 | 14.82 | 167.03 | 86.41 |
| A14 | F | 66 | 0.70 | 4.26 | 12.68 | ＞1000 | ND |
| A15 | F | 59 | 3.72 | 4.40 | 15.97 | ND | 1651.48 |
| A16 | M | 36 | 0.65 | 4.18 | 11.26 | 126.18 | 621.18 |
| A17 | F | 67 | 1.63 | ND | 10.81 | 912.52 | 18.52 |
| A18 | F | 57 | 1.27 | ND | 13.45 | 258.95 | 5.44 |
| A19 | F | 64 | 1.04 | 4.72 | 5.36 | ＞1000 | 0.61 |
| A20 | F | 30 | 1.66 | 3.94 | 3.17 | ＞1000 | 0.47 |
| A21 | F | 46 | ＜0.004 | 3.99 | 11.82 | 13.44 | ＞1000 |
| A22 | F | 62 | 2.45 | 3.93 | 11.03 | 587.86 | ND |
| A23 | F | 56 | 1.25 | 4.70 | ND | 63.76 | 37.52 |
| A24 | F | 44 | 1.92 | 4.29 | 11.45 | 57.23 | 27.31 |
| A25 | F | 53 | 1.08 | ND | 12.36 | 540.22 | 0.73 |
| A26 | M | 48 | 1.25 | 11.75 | 32.15 | ＞4000 | 139.7 |
| A27 | F | 59 | 0.83 | ND | 10.47 | ND | ND |
| A28 | F | 56 | 1.66 | 4.25 | 12.50 | ND | ND |
| A29 | F | 43 | 1.65 | 4.27 | 12.45 | 1692.47 | 241.37 |
| A30 | F | 35 | 1.79 | 4.64 | 15.91 | 2.55 | 51.82 |
| A31 | F | 31 | 0.01 | 5.17 | 13.34 | 2.81 | 634.47 |
| A32 | F | 38 | 1.35 | 3.67 | 10.38 | 9.93 | 37.34 |
| A33 | M | 39 | 2.14 | 4.75 | 13.51 | 1.48 | ND |
| A34 | F | 34 | 0.66 | 4.28 | 12.16 | 58.36 | 112.16 |

F, female; M, male; TSH, thyroid-stimulating hormone (Reference: 0.27 - 5.0 mUI/L); FT4, free thyroxine (Reference: 10.3 - 31.0 pmol/L); FT3, Free Triiodothyronine (Reference: 2.0 - 6.8 pmol/L); TPO Ab, anti-thyroid peroxidase antibodies (Reference: <9 UI/mL); Tg Ab, anti-thyroglobulin antibodies (Reference: <4 UI/mL); y, years; ND, not determined.

**Table S3. Potential m^6^A modification sites on KDR mRNA predicted by SRAMP.**

| Position | Score | Confidence | Position | Score | Confidence |
| --- | --- | --- | --- | --- | --- |
| 467 | 0.591 | Moderate | 2872 | 0.603 | High |
| 475 | 0.655 | High | 2877 | 0.579 | Moderate |
| 481 | 0.641 | High | 2892 | 0.656 | High |
| 531 | 0.593 | Moderate | 2957 | 0.646 | High |
| 561 | 0.534 | Low | 3120 | 0.578 | Moderate |
| 592 | 0.564 | Moderate | 3151 | 0.706 | Very high |
| 624 | 0.541 | Low | 3193 | 0.696 | Very high |
| 628 | 0.581 | Moderate | 3295 | 0.689 | Very high |
| 1007 | 0.585 | Moderate | 3385 | 0.589 | Moderate |
| 1050 | 0.658 | High | 3439 | 0.581 | Moderate |
| 1055 | 0.622 | High | 3669 | 0.580 | Moderate |
| 1072 | 0.613 | High | 3724 | 0.754 | Very high |
| 1115 | 0.621 | High | 3752 | 0.534 | Low |
| 1129 | 0.536 | Low | 3776 | 0.604 | High |
| 1731 | 0.535 | Low | 3823 | 0.660 | High |
| 1771 | 0.640 | High | 3849 | 0.673 | Very high |
| 1839 | 0.540 | Low | 3875 | 0.684 | Very high |
| 2023 | 0.548 | Low | 3979 | 0.552 | Low |
| 2116 | 0.652 | High | 4040 | 0.541 | Low |
| 2170 | 0.543 | Low | 4078 | 0.671 | High |
| 2209 | 0.627 | High | 4113 | 0.579 | Moderate |
| 2218 | 0.669 | High | 4206 | 0.549 | Low |
| 2239 | 0.586 | Moderate | 4240 | 0.562 | Moderate |
| 2258 | 0.622 | High | 4246 | 0.592 | Moderate |
| 2431 | 0.616 | High | 4277 | 0.715 | Very high |
| 2458 | 0.550 | Low | 4339 | 0.555 | Low |
| 2587 | 0.670 | High | 4347 | 0.582 | Moderate |
| 2664 | 0.650 | High | 4403 | 0.671 | High |
| 2690 | 0.664 | High | 4500 | 0.570 | Moderate |
| 2732 | 0.640 | High | 4507 | 0.645 | High |
| 2747 | 0.616 | High | 5180 | 0.628 | High |
| 2797 | 0.589 | Moderate |  |  |  |

**Table S4. Chemical and biological reagents used in this study.**

| Chemicals | Company | Cat. No | Application |
| --- | --- | --- | --- |
| Complete Freund’s Adjuvant (CFA) | Macklin | F850325 | animal model |
| Incomplete Freund’s Adjuvant (IFA) | Macklin | F850326 | animal model |
| Potassium iodide (KI) | Beyotime | Y000255 | animal model |
| IFN-γ | Beyotime | P5664 | Cell culture |
| H_2_O_2_ | Merck | 18304 | Cell culture |
| N-acetyl-L-cysteine (NAC) | Beyotime | S0077 | Cell culture |
| VEGF165 / rhVEGFA | MCE | HY-P7110A | Cell culture |
| Penicillin-Streptomycin-Amphotericin B | Beyotime | C0224 | Cell culture |
| LPS | Beyotime | ST1470 | Cell and animal model |
| Porcine thyroid (pTg) | Merck | T1126 | Cell and animal model |
| Matrigel (growth factor-reduced) | BD | 354230 | Cell and animal model |
| Cell Counting Kit-8 | Selleck | B34302 | cell viability |
| Magna ChIP Kit | Merck | 17-10085 | ChIP |
| Methylene Blue, Sodium Acetate | Aladdin | M196499 | Dot blot |
| Human TgAB ELISA | mlbio | ml025773 | TgAB level |
| mouse TgAB ELISA | mlbio | ml037680 | TgAB level |
| Human TPOAB ELISA | mlbio | ml025777 | TPOAB level |
| mouse TPOAB ELISA | mlbio | ml002215 | TPOAB level |
| Human T3 ELISA | mlbio | ml105456 | T3 level |
| Human T4 ELISA | mlbio | ml106408 | T4 level |
| EpiQuik m^6^A RNA Methylation Kit | Epigentek | P-9005 | m^6^A RNA level |
| Human IFN-γ ELISA | mlbio | ml077386 | IFN-γ level |
| mouse IFN-γ ELISA | mlbio | ml059749 | IFN-γ level |
| Rat IFN-γ ELISA | mlbio | ml064291 | IFN-γ level |
| Human TNFα ELISA | mlbio | ml077385 | TNFα level |
| mouse TNFα ELISA | mlbio | ml002095 | TNFα level |
| Rat TNFα ELISA | mlbio | ml002859 | TNFα level |
| Human IL-6 ELISA | mlbio | ml058097 | IL-6 level |
| mouse IL-6 ELISA | mlbio | ml063159 | IL-6 level |
| Rat IL-6 ELISA | mlbio | ml064292 | IL-6 level |
| Rat IL-1β ELISA | mlbio | ml037361 | IL-1β level |
| mouse IL-4 ELISA | mlbio | ml064310 | IL-4 level |
| mouse IL-17A ELISA | mlbio | ml037864 | IL-17A level |
| mouse VEGFA ELISA Kit | mlbio | ml037273 | VEGFA level |
| Human VEGFA ELISA Kit | mlbio | ml060752 | VEGFA level |
| GSK1120212 (Trametinib) | MCE | HY-10999 | ERK inhibition |
| MK-2206 | MCE | HY-108232 | AKT inhibition |
| Annexin V-FITC/PI Apoptosis Kit | Vazyme | A211 | FASC |
| Reactive Oxygen Species Assay Kit | Beyotime | S0033S | FASC |
| Sodium butyrate (NaBu) | MCE | HY-B0350A | HDAC inhibition |
| BCA Protein Assay Kit | Beyotime | P0009 | Immunoblot |
| ECL Chemiluminescence kit | Biosharp | BL523B | Immunoblot |
| Triton X-100 | Beyotime | P0096 | IHC/IF |
| Goat serum | Boster | AR1009 | IHC/IF |
| DAPI | Boster | AR1176 | IHC/IF |
| Dual-luciferase reporter assay | Beyotime | RG027 | Luciferase activity |
| RIPA | Beyotime | P0038 | Cell and tissue lysate |
| Phosphatase Inhibitor Cocktail I&II | MCE | HY-K0021/22 | Cell and tissue lysate |
| PMSF | MCE | HY-B0496 | Cell and tissue lysate |
| Collagenase IV | BioFroxx | 2091MG100 | Cell and tissue lysate |
| DNase I | Beyotime | D7073 | Cell and tissue lysate |
| Western/IP Lysis Buffer | Beyotime | P0013 | Cell and tissue lysate |
| Peripheral Blood Monocyte Isolation | Solarbio | P8680 / P6700 | PBMC isolation |
| Human M-CSF protein | MCE | HY-P7085 | PBMC isolation |
| Magna RIP / MeRIP Kits | Merck | 17-700 | RIP/MeRIP |
| Trizol reagent | Vazyme | R401 | RNA extraction |
| HiScript II Reverse Transcriptase | Vazyme | R201 | Reverse transcription |
| ChamQ SYBR qPCR Master Mix | Vazyme | Q711 | RT-qPCR |
| actinomycin D | MCE | HY-17559 | Transcription inhibition |
| Lipofectamine 3000 | ThermoFisher | L3000015 | Transfection |
| Polyethyleneimine (PEI) | Beyotime | C0541 | Transfection |
| Polybrene | Beyotime | C0351 | Transfection |
| Sunitinib | MCE | HY-10255A | VEGFR2 inhibition |
| Cycloheximide (CHX) | MCE | HY-12320 | Translation inhibition |
| DMSO | Merck | D8418 | Solvent |
| pECE-FLAG-SIRT1-H363Y | MiaoLing | P1088 | Gene over-expression |
| pCMV-3-METTL3(human)-Puro | MiaoLing | P83910 | Gene over-expression |
| pEF1a-Mettl3(rat)-EGFP | MiaoLing | P50169 | Gene over-expression |
| pLV3-CMV-Sirt1(rat)-Puro | MiaoLing | P43878 | Gene over-expression |
| pCMV-SIRT1(human)-Neo | MiaoLing | P34489 | Gene over-expression |

**Table S5. Antibodies used in this study.**

| Antibody | manufacturer | Cat No |
| --- | --- | --- |
| N6-methyladenosine (m^6^A) | Abcam | ab284130 |
| METTL3 | Proteintech | 67733-1-Ig |
| METTL14 | Proteintech | 26158-1-AP |
| FTO | Proteintech | 68111-1-Ig |
| ALKBH5 | Proteintech | 67811-1-Ig |
| WTAP | Proteintech | 60188-1-Ig |
| SIRT1 | Proteintech | 60303-1-Ig |
| SIRT2 | Proteintech | 66410-1-Ig |
| SIRT4 | Proteintech | 66543-1-Ig |
| SIRT7 | Proteintech | 12994-1-AP |
| HDAC4 | Proteintech | 66838-1-Ig |
| HDAC5 | Proteintech | 68437-1-Ig |
| HDAC8 | Proteintech | 17548-1-AP |
| KAT2B | Proteintech | 28770-1-AP |
| KAT8 | Proteintech | 13842-1-AP |
| VEGFR2/KDR | Proteintech | 83049-4-RR |
| Phospho-VEGF Receptor 2-Y1175 | ABclonal | AP1385 |
| Phospho-ERK1/2 (Thr202/Tyr204) | Proteintech | 80031-1-RR |
| ERK1/2 | Proteintech | 66192-1-Ig |
| Phospho-AKT-S473 | ABclonal | AP0637 |
| AKT | Proteintech | 10176-2-AP |
| Phospho-FAK-Y397 | ABclonal | AP1447 |
| Phospho-p38 MAPK-T180/Y182 | ABclonal | AP1311 |
| Phospho-PLC gamma 2 (PLCG2)-Y759 | ABclonal | AP1519 |
| Histone H3 (acetyl K27) | Abcam | ab4729 |
| Histone H3 (tri methyl K4) | Abcam | ab8580 |
| CD31/PECAM1 | ABclonal | A19014 |
| Caspase-3 | ABclonal | A25309 |
| Ki-67 | ABclonal | A20018 |
| VE-cadherin | ABclonal | A12416 |
| Ms CD45 APC-Cy7 30-F11 | BD | 557659 |
| APC/Cyanine7 anti-rat CD45 | Biolegend | 202216 |
| APC/Cyanine7 anti-human CD45 | Biolegend | 304014 |
| V450 -Anti-Mouse/Human CD11b | Elabscience | E-AB-F1081Q |
| V450 Rat anti-CD11b | BD | 560455 |
| Ms F4/80 PE T45-2342 | BD | 565410 |
| PE anti-rat CD172a (SIRPα) | Biolegend | 204706 |
| PE-Anti-Human HLA-DR (L243) | Elabscience | E-AB-F1111E |
| PE-Cyanine5 CD86 (B7-2) | eBioscience™ | 15-0862-82 |
| PE-Cyanine5 Anti-Human CD86 | Elabscience | E-AB-F1012M |
| FITC anti-rat CD86 | Biolegend | 200305 |
| Human CD3/CD28 T Cell Activation Beads | Biolegend | 422603 |
| Goat Anti-Rabbit IgG H&L (HRP) | Abcam | ab6721 |
| Goat Anti-Rabbit IgG H&L (FITC) | Abcam | ab6717 |
| Goat Anti-Rabbit IgG H&L (Cy3) | Abcam | ab6939 |

Table S6. The shRNA target sequences in this study.

| shRNAs | Sequence (5’-3’) |
| --- | --- |
| METTL3 sh1 (homo) | GCTGCACTTCAGACGAATTAT |
| METTL3 sh2 (homo) | CCAGTCATAAACCAGATGAAA |
| *Mettl3* sh1 (rat) | GGATTGCGATGTGATTGTA |
| *Mettl3* sh2 (rat) | CAGTGGATCTGTTGTGATA |
| *Mettl3* sh1 (mouse) | CGATGTTGATCTGGAGATA |
| *Mettl3* sh2 (mouse) | CGAAAGAACAGCAGAGCAA |
| KDR sh1 (homo) | GACTGGCTTTGGCCCAATAAT |
| KDR sh2 (homo) | GATGAAAGTTACCAGTCTATT |
| *Kdr* sh1 (rat) | GGTCAAGATTGATGAAGAA |
| *Kdr* sh2 (rat) | CGTTTATGTCTATGTTCAA |
| SIRT1 sh1 (homo) | CAGGTCAAGGGATGGTATTTA |
| SIRT1 sh2 (homo) | GAGACTGTGATGTCATAATTA |
| *Sirt1* sh1 (rat) | GGCAGTTAATGAAGCTATA |
| *Sirt1* sh2 (rat) | GAACAAAGTTGACGATTTA |
| YTHDC2 sh1 (homo) | CCCTCGTCACATCTCTTATAT |
| YTHDC2 sh2 (homo) | GCCCACAGATTGGCTTATTTA |
| *Ythdc2* sh1 (rat) | GGATATACAAACAAAGAAA |
| *Ythdc2* sh2 (rat) | GGAGCAAATAGATACCTAA |

Table S7. The primer sequences for the METTL3 promoter region in the ChIP-qPCR.

|  | Forward Primer | Reverse Primer |
| --- | --- | --- |
| Site 1 | TAGCTTCCAAGAAAGCGCGA | TTTCTCTAGGGATCCCGCCC |
| Site 2 | CCAGAGTCACTGCCCTTTACC | CGCAGTCTGAAGAGGAGTGG |
| Site 3 | TTTCTTGGCAGCTGTGGGTA | TCAATGCCGGTAGGGGTACT |
| Site 4 | ATGTGCTCGTATTCTGGCCC | AGCGTCTCCCCTTGAATCCT |

Table S8. The primers used for the KDR transcripts in RIP-qPCR and MeRIP-qPCR.

| Position | Forward Primer | Reverse Primer | Length |
| --- | --- | --- | --- |
| 363-636 | TCTGTGGGTTTGCCTAGTGT | AGGCCAAGTCAGTTTCCCG | 274 |
| 974-1205 | TGATGTGGTTCTGAGTCCGTC | GTCACTCCGGGTTACACCAT | 232 |
| 1672-1923 | ACTGGTATTGGCAGTTGGAGG | TCTCTCCTCTCCCGACTTTGT | 252 |
| 2000-2241 | CGTGTCTTTGTGGTGCACTG | TGTCTTGAGCAAGGCAGACA | 242 |
| 2371-2668 | CATCTGGGAATCCCCCTCCA | ACGGTCCGTAGGATGATGAC | 298 |
| 2569-2818 | GTGCCCAGGAAAAGACGAAC | GGCTTACCTAGCTTCAGCCG | 250 |
| 2806-3024 | AGCTAGGTAAGCCTCTTGGC | GCTTGGTACAGGCACCTAGAA | 219 |
| 3159-3369 | GGAGCAATCCCTGTGGATCT | TTCGCGATGCCAAGAACTCC | 211 |
| 3386-3684 | CCTGGCGGCACGAAATATCC | GGGCCCTCATTCTAGTTCCT | 299 |
| 3751-3999 | GACCCACGTTTTCAGAGTTGG | GCCGGCTCTTTCGCTTACTG | 249 |
| 4067-4329 | CAACCAGACGGACAGTGGTA | GAATCTGGGCTGTGCTACCG | 263 |
| 4292-4474 | AGAGATTGGAGTGCAAACCG | TCAAATGCGGCTACTTCCTGC | 183 |
| 4452-4689 | CAGCAGGAAGTAGCCGCATT | GATGGGGCCATTTCTTGAACG | 238 |
| 4920-5216 | TGCATTGTGTTTGCTCTGGTG | AACTCTTCAACACGGCAGGG | 297 |
| 4162-4369 | CCAGCAAAAGCAGGGAGTCT | ACAGGAGGAGAGCTCAGTGT | 208 |
| 4387-4512 | CACCCCCAACTCCTGGACAT | TGCAGTCCGAGGTCCTTTTTC | 126 |
| 4577-4837 | TCCCAGTGTTGACCTGATCC | TTGGGACCCACGTCCTAAAC | 261 |
| 4788-5090 | TTGCAGGGCTGAGTCTATCC | ACACGTAACGGTCTGGAAGG | 155 |
| 4521-4675 (Rat *Kdr*) | TATATTGTGCCCTGCTGCGG | CATCTCACCCATCCCAACACA | 161 |

Table S9. The primer sequences used for RT-qPCR in this study.

| Gene | Forward Primer | Reverse Primer |
| --- | --- | --- |
| GAPDH | TGACATCAAGAAGGTGGTGA | TCCACCACCCTGTTGCTGTA |
| TPO | GCAGTTGGCTGAGAAGAGGA | TCCCTCTCGAGATGAAGGGG |
| Tg | TTCTCCTCCTTCCTCCCAGG | TCAGAAAGGCCGTTTCCCTC |
| PTPRC | CACCTAGCAGTTCATGCAGC | GGCAAAGCCAAATGCCAAGA |
| PECAM1 | TTTCTGCTTTTCACAGGGCG | AGAGTGAAGACTGCAGGCAC |
| METTL3 | CAAGGCTTCAACCAGGGTCT | GGGTTGCACATTGTGTGGTC |
| METTL14 | GTAGCACAGACGGGGACTTC | GCCAGCCTGGTCGAATTGTA |
| WTAP | CAGCTGCTCCATTGTGCCTC | ATCTCAGTTGGGCAACGCTC |
| KAT1 | TCGGAAATGGCGGGATTTGG | ACGGAACATTGTTGACAGGC |
| KAT2A | AAGCTAGGGGTCTTCTCGGC | AAGTGGGATACGTGGTCAGC |
| KAT2B | CCGAATCGCCGTGAAGAAAG | AGCTAGGGCATGGCTACAAC |
| KAT5 | AGTGGAGGGAGGGAAGATGG | TCTTCGTTGTCCTGGTTCCG |
| KAT7 | CTCTTCGGCAGACTCGTTCA | TTCCAGTTGGAGTTCGAGGC |
| KAT8 | GGAGAAGCCACTGTCTGACC | GGAGTCCACTGTGATGGGTG |
| HDAC1 | GACCGACTGACGGTAGGGAC | ATTGGCTTTGTGAGGGCGAT |
| HDAC2 | CTTCCCCGCGGGACTATC | CCGCCTCCTTGACTGTACG |
| HDAC3 | GGCCTATTTCTACGACCCCG | GCATATTGGTGGGGCTGACT |
| HDAC4 | CCCTCTACACATCGCCATCC | TACCAGTCTGTGACGAGGGG |
| HDAC5 | TCCCGTCCGTCTGTCTGTTA | GACCTGACATCCCATCCGAC |
| HDAC6 | TGGCGGAGTGGAAGAACCG | TGCAGTCCCACGATTAGGTC |
| HDAC7 | CTTCCTCTCGCCGTCTCAC | CAGCACTTCGCTTGCTCTTG |
| HDAC8 | GCAGCATCTCCAGAAGGTCA | GCACATTCCGTCAATCAGGC |
| HDAC9 | GCAACAAAACCCTAGCAGCC | CCCGGAAACTACAAGCAGGT |
| HDAC10 | ATGGCCAGGGGATCCAGTAT | AGTCAAATCCTGCCGAGACC |
| HDAC11 | GCTCAAGTGGTCCTTTGCTG | CAGAAACTTGATGGCGAGCG |
| SIRT1 | TGGGTACCGAGATAACCTTCT | TGCCAATCATAAGATGTTGCTG |
| SIRT2 | GACACAGTGGTTGGTGACGG | TGGATGGAGAGCGAAAGTCG |
| SIRT3 | GGGTAGTTGAACGGGTCGAG | AAGTCTGGAATGCCACTGGG |
| SIRT4 | ATGCAATCAGACGGTCCCAC | AGCCTACGAAGTTTCTCGCC |
| SIRT5 | CGGGGCCCAAGTAAATGGAA | AGAGGTCGCATCAGGGTTTG |
| SIRT6 | GATGTCGGTGAATTACGCGG | ACACCACACTGGAAGACTGC |
| SIRT7 | CTTGGTCGTCTACACAGGCG | GGAACGCAGGAGGTACAGAC |
| AMZ1 | ACTTTGCACTAGGTGCTGACA | GAACTCCTGTGCGGGTCTAC |
| ZNF511 | GGAGGCCATGGAAATCTGCT | TTAAATCCTCGAGCGGCACC |
| RNF165 | GCTTCCAGTGTTTGGCTCTG | GGTAGGAAGGAGGGACCTGT |
| FZD1 | ACCAACAGCAAACAAGGGGA | AGGTCTGTCCATCCTCCCTC |
| ADGRA1 | TTGATAACTCGGTCCCAGCTC | AAGGTCAAAGTCCCAGACGG |
| KDR | CGGTCAACAAAGTCGGGAGA | CAGTGCACCACAAAGACACG |
| KDR | GATGACACAGACACCACCGT | GTGATGTCCAGGAGTTGGGG |
| GDF15 | GCAAGAACTCAGGACGGTGA | TGGAGTCTTCGGAGTGCAAC |
| PGAP1 | TGTGGGATGTCTTCTTCGGC | GGCCACCCATAGAATGACCA |
| CALCOCO1 | AGGCTCAGCGACTGAAAGAC | CCTTCAAGTGCAAACCGAGC |
| KLHL24 | CGAGGAACCGGTGTGGAAAT | AGCACAGCTCTATGGCAAGG |
| *Actb* (rat) | TGAGCTGCGTTTTACACCCT | GCCTTCACCGTTCCAGTTTT |
| *Tpo* (rat) | CGGTGTGGGGACAGTACATC | TCCGTGAGGAGTTTGAGGGA |
| *Tg* (rat) | TGTGAGATAAGAAGCCGCCG | GAATGGCCAGGAATCGTCTCT |
| *Ptprc* (rat) | TTCGGCTTTGCCTTTCTGGA | TGGGAGTGAGAATGCAGTGG |
| *Pecam1* (rat) | AGTGTGGAAACCAACAGCCA | ATCAAGGGAGCCTTCCGTTC |
| *Mettl3* (rat) | CGTAGTGATAGTCCCGTGCC | TGGCAAGACGGATGGAAACA |
| *Kdr* (rat) | CGTGTACTCCAGCGACGAG | TCAAATGCGGCTACTTCCGA |
| *Ythdc2* (rat) | CGACGAGGAGGTGAAGATCG | GGTCATCATTGCATGAGCTGT |
| *Mettl14* (rat) | AGTTTGGGAGCTGAGAGTG | GTATCATAGGAAGCCCTGCA |
| *Wtap* (rat) | AGAACATCCTTGTCATGCGGCTAG | CGGCTTCAAGCTGTGCAATACG |
| *Fto* (rat) | AATGAAGACGCTGTGCCGTT | GAAGCTGGACTCGTCATCGC |
| *Alkbh5* (rat) | TACTTCTTCGGCGAGGGCTA | CGTTGATGACTGCGCTGTTG |
| *Tpo* (mouse) | AGGGTCCTCCTGTGCGAATA | CTGGAGTCTATGCCAGCGTC |
| *Tg* (mouse) | GGACTGCCTTGTCACTACCC | CACCTTGATGACCTGGGACC |
| *Ptprc* (mouse) | AGATAGGCGCATCAGAAGGG | CAGCCTGAAGGTACATCAGCA |
| *Pecam1* (mouse) | CAAGGCCAAACAGAAACCCG | TCGACCTTCCGGATCTCACT |
| *Mettl3* (mouse) | CCGTAGTGATAGTCCCGTGC | TGGCGTAGAGATGGCAAGAC |
| *Mettl14* (mouse) | TGGATTTGCATTTTGGCGGG | ATGCTATCCGCACTCTCAGC |
| *Wtap* (mouse) | CTTCCGCGGACTGTCTCC | TCGTTGGTCATCTTGCACCC |
| *Fto* (mouse) | GGCTCACAGCCTCGGTTTAG | ATTTCAACGAGACGTCGCCA |
| *Alkbh5* (mouse) | CCACATTGCCACCCAGCTAT | AGACCGCCGGTTTTCTTCTT |
| *Tnf* (mouse) | TGTAGCCCACGTCGTAGCAA | ATAGCAAATCGGCTGACGGT |
| *Il12a* (mouse) | GCACTTGTCCTTGAGATGTAGA | AGCTCCCTCTTGTTGTGGAAG |
| *Il6* (mouse) | CCCCAATTTCCAATGCTCTCC | CGCACTAGGTTTGCCGAGTA |
| *Il1b* (mouse) | TGCCACCTTTTGACAGTGATG | AAGGTCCACGGGAAAGACAC |
| *Il4* (mouse) | CCCCCAGCTAGTTGTCATCC | AGGACGTTTGGCACATCCAT |
| *Il17a* (mouse) | GCTGACCCCTAAGAAACCCC | GAAGCAGTTTGGGACCCCTT |
| *Il12b* (mouse) | TGGAGCACTCCCCATTCCTA | GAGCTTGCACGCAGACATTC |
| *Ifng* (mouse) | CGGCACAGTCATTGAAAGCC | TGCATCCTTTTTCGCCTTGC |
| *Ccl2* (mouse) | AGGTGTCCCAAAGAAGCTGT | AAGACCTTAGGGCAGATGCAG |
| *Ccl5* (mouse) | GCTCCAATCTTGCAGTCGTG | GAGCAGCTGAGATGCCCATT |
| *Cxcl1* (mouse) | ACTCAAGAATGGTCGCGAGG | GTGCCATCAGAGCAGTCTGT |
| *Cxcl3* (mouse) | CCAACGGTGTCTGGATGTGT | CACCGGCATGACCTTGTTTG |
| *Cxcl9* (mouse) | AACGTTGTCCACCTCCCTTC | CACAGGCTTTGGCTAGTCGT |
| *Cxcl10* (mouse) | GCAGGATGATGGTCAAGCCA | AGAGCTAGGACAGCCATCCC |
| *Cxcl12* (mouse) | GCCCTTCAGATTGTTGCACG | CATCTCCCACGGATGTCAGC |

1. **Supplementary Figures**

**Supplementary figure 1.**


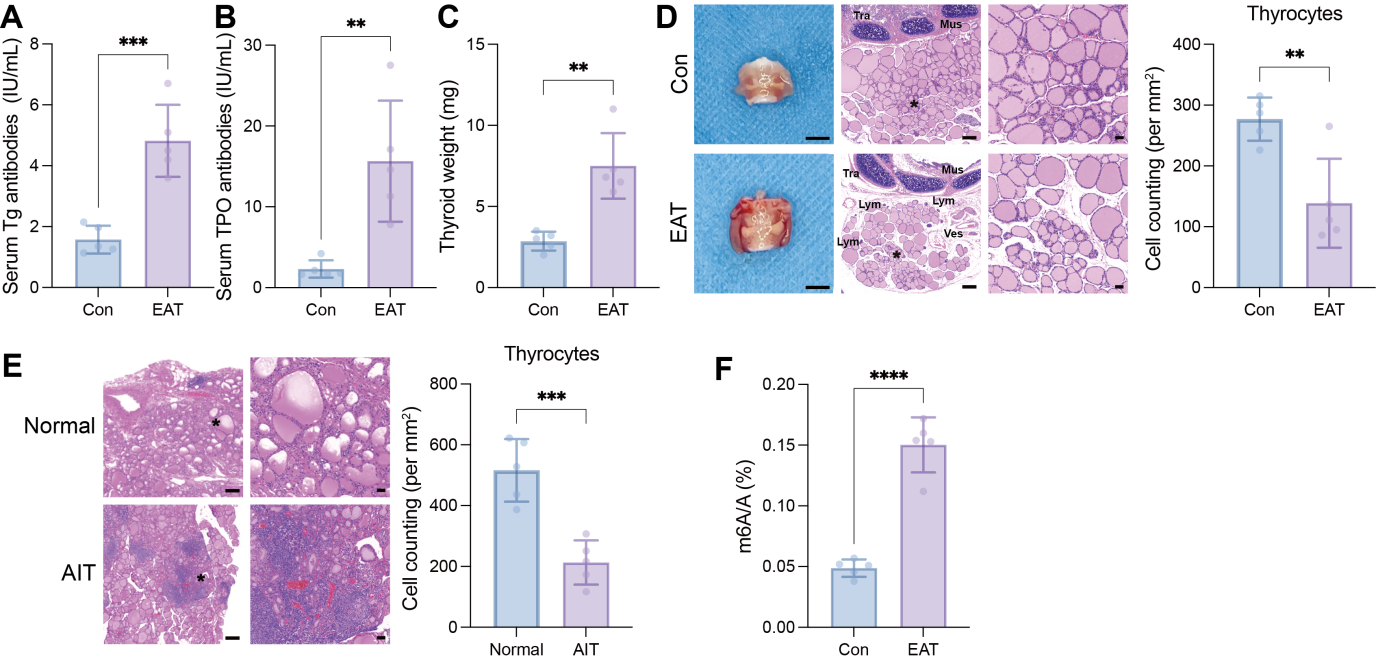


**Figure S1. Establishment of the EAT model and increased m6A RNA modification in thyroid tissues.** (A, B) Plasma TgAb and TPOAb levels in control and EAT mice. (C) Thyroid weight in the indicated groups. (D) Representative gross morphology, H&E staining images and thyrocyte counts of thyroid tissues. Macroscopic scale bar, 2 mm; scale bars, 100 μm for low-magnification images and 20 μm for high-magnification images. At low magnification, the overall anatomical structure of the thyroid gland is shown, including its spatial relationship with surrounding tissues, such as the trachea (Tra) and adjacent muscle (Mus). At high magnification, compared with control tissues, EAT tissues exhibited disrupted follicular structures, increased lymphocytic infiltration (Lym), and expanded vessels (Ves) within and surrounding the follicles. (E) Representative H&E staining images and thyrocytes counts of thyroid tissues from normal and AIT. Scale bars, 200 μm for low-magnification and 50 μm for high-magnification. Black asterisks in the low-magnification panels indicate the areas shown at higher magnification. (F) ELISA analysis of m6A RNA modification levels in thyroid tissues from control and EAT mice. **Data information**: Data are presented as mean ± SD, n = 3. Statistical significance was determined using an unpaired two-tailed Student’s t-test for two-group comparisons. ns, not significant; *P < 0.05; **P < 0.01; ***P < 0.001; ****P < 0.0001.

**Supplementary figure 2.**


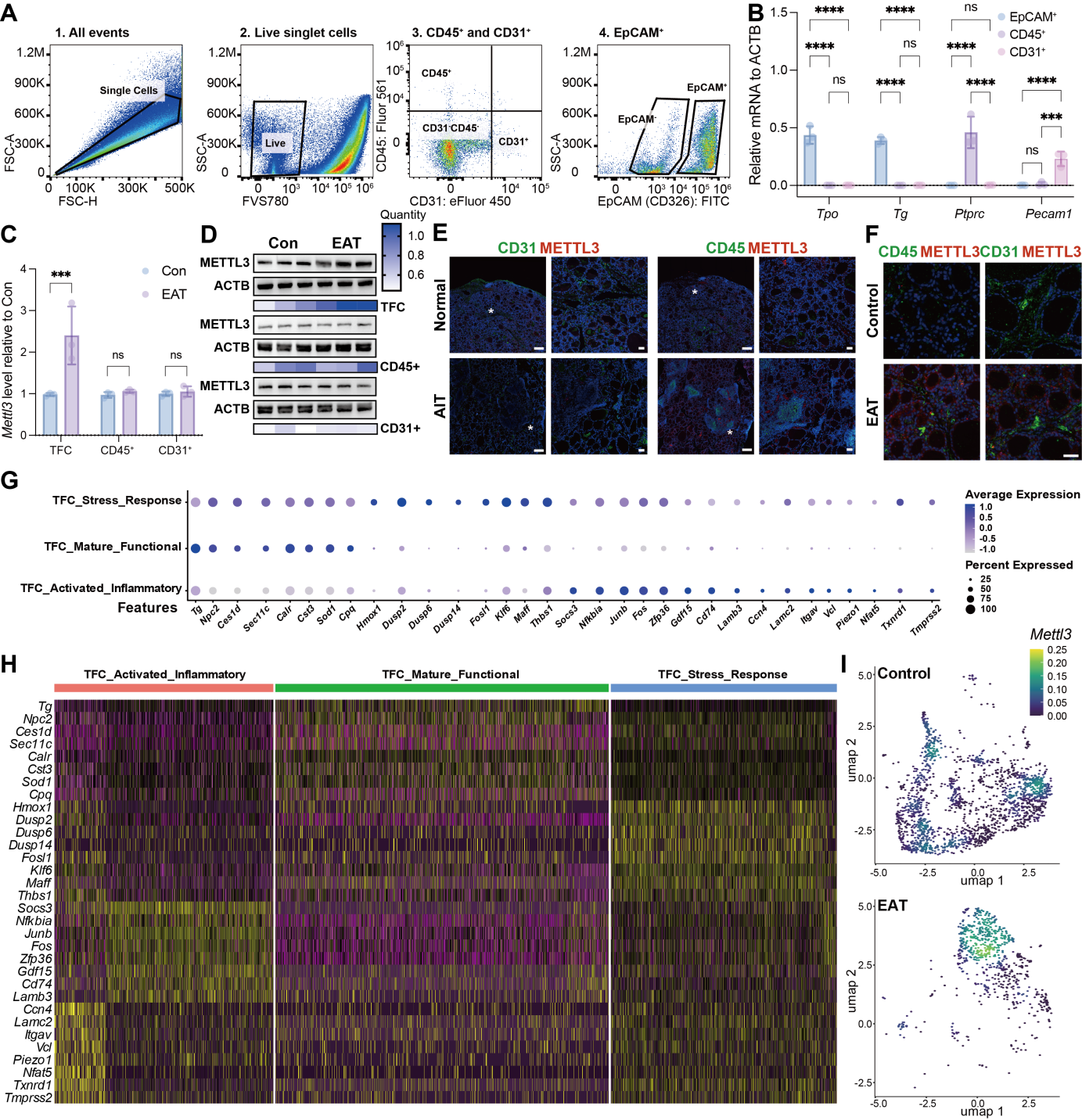


**Figure S2. METTL3 upregulation in autoimmune thyroiditis is predominantly restricted to TFCs.** **(A)** Flow cytometric gating strategy for sorting EpCAM^+^ epithelial cells, CD45^+^ immune cells, and CD31^+^ endothelial cells from thyroid tissues. **(B)** RT-qPCR analysis of *Tpo*, *Tg*, *Ptprc*/CD45, and *Pecam1*/CD3**1** in sorted cell populations to verify cell identity and sorting purity. **(C)** RT-qPCR analysis of *Mettl3* expression in sorted TFCs, CD45^+^ immune cells, and CD31^+^ endothelial cells from control and EAT mice. **(D)** Western blot analysis of METTL3 protein expression in the indicated sorted cell populations. (E) Representative IF co-staining of METTL3 with CD31 and CD45 in thyroid tissues from AIT patients. Scale bars, 200 μm for low-magnification images and 50 μm for high-magnification images. White asterisks in the low-magnification panels indicate the areas shown at higher magnification. **(F)** Representative IF co-staining of METTL3 with CD31, and CD45 in thyroid tissues from control and EAT mice. Scale bar, 50 μm. (G) Dot plot showing representative marker genes defining three TFCs subpopulations (Mature, Stress, and Inflammatory) identified by scRNA-seq. Dot size represents the percentage of expressing cells, and color indicates scaled average expression. (H) Feature plot showing the expression distribution of *Mettl3* across TFCs subpopulations identified by scRNA-seq. Cells are projected onto a UMAP embedding and colored according to normalized *Mettl3* expression levels. **Data information**: Values are mean ± SD, n=3. Statistical analyses were performed using one-way ANOVA with Tukey’s post hoc test for B, and unpaired two-tailed Student’s t-test for others. ns, not significant; *P < 0.05; **P < 0.01; ***P < 0.001; ****P < 0.0001.

**Supplementary figure 3.**


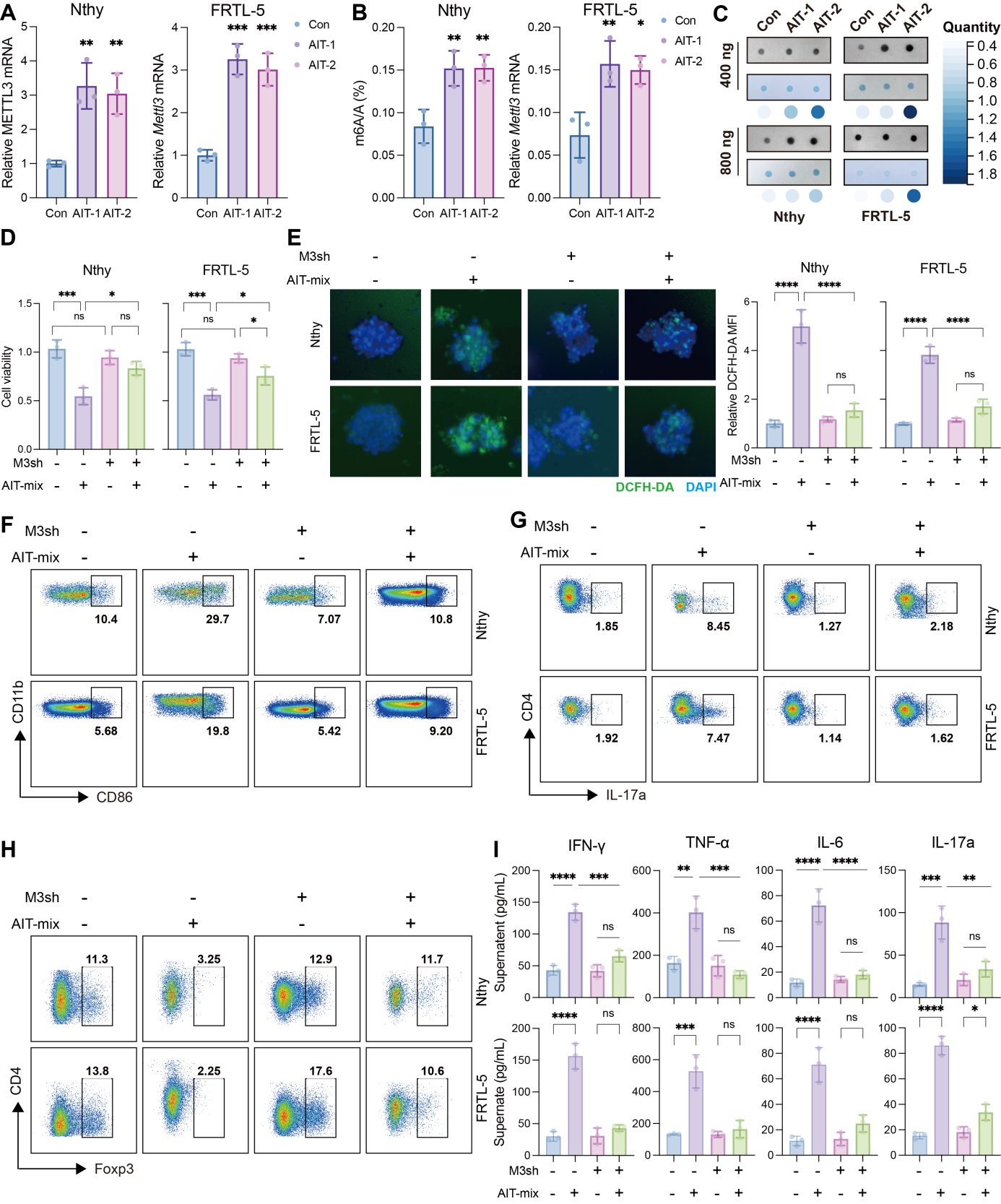


**Figure S3. AIT-mix induces METTL3 upregulation, and immune dysregulation in vitro, which are alleviated by METTL3 knockdown. (A)** RT-qPCR analysis of **METTL3** expression in TFCs after AIT-mix stimulation. **(B)** ELISA-based quantification of total m6A RNA methylation in TFCs after AIT-mix treatment. **(C)** Dot blot analysis further confirming increased m6A RNA methylation in AIT-mix-treated TFCs. **(D)** CCK-8 assay evaluating TFCs viability under the indicated conditions. **(E)** Representative DCFH-DA fluorescence images and quantitative analysis showing intracellular ROS accumulation in TFCs. Scale bars, 300 µm. **(F)** Representative flow cytometry plots of CD11b^+^CD86^+^ macrophages showing enhanced M1 polarization in the co-culture system. **(G)** Representative flow cytometry plots of CD4^+^IL-17A^+^ Th17 cells. **(H)** Representative flow cytometry plots of CD4^+^FOXP3^+^ Treg cells. **(I)** ELISA analysis of TNF-α, IFN-γ, IL-6, and IL-17A levels in culture supernatants of Nthy co-culture and FRTL-5 co-culture system. **Data information**: Data are presented as mean ± SD. Statistical analyses were performed using uone-way ANOVA with Tukey’s post hoc test as appropriate. ns, not significant; *P < 0.05; **P < 0.01; ***P < 0.001; ****P < 0.0001.

**Supplementary figure 4.**


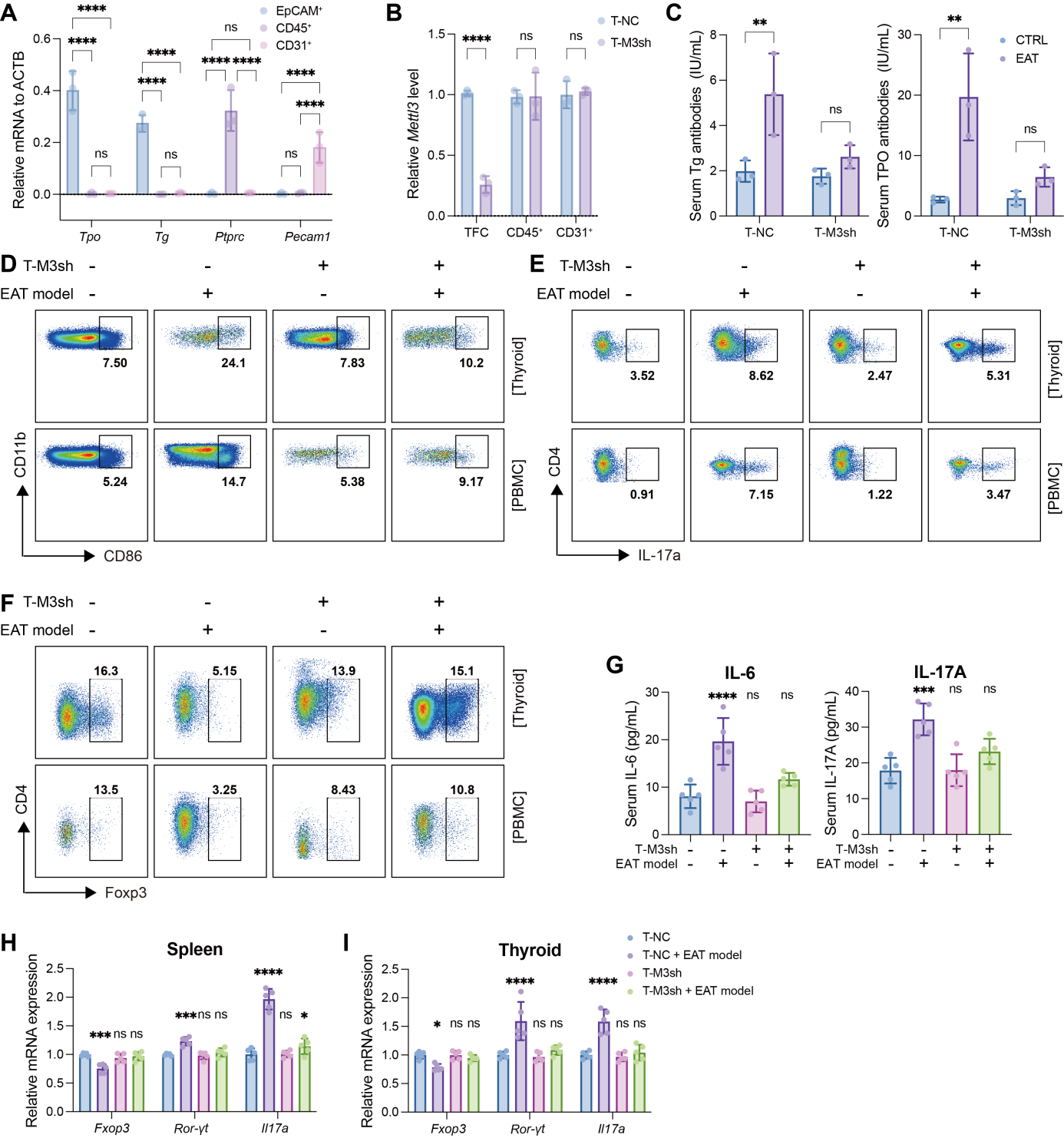


**Figure S4. In vivo thyroid-targeted METTL3 knockdown is selective for TFCs and mitigates autoimmune inflammation. (A)** RT-qPCR validation of ***Tpo*,** ***Tg***, ***Ptprc***, and ***Pecam1*** expression in sorted EpCAM^+^ TFCs, CD45^+^ immune cells, and CD31^+^ endothelial cells, confirming cell identity and sorting purity. **(B)** RT-qPCR analysis showing selective reduction of **METTL3** expression in TFCs, but not in CD45^+^ immune cells or CD31^+^ endothelial cells, from T-M3sh mice relative to T-NC controls. **(C)** Serum TgAb and TPOAb levels measured by ELISA. **(D, E, F)** Representative flow cytometry plots of M1, Th17 and Treg cells showing restoration of M1 polarization, Th17/Treg balance after thyroid-targeted **METTL3** knockdown. **(G)** ELISA analysis of serum IL-6, and IL-17A levels showing reduced inflammatory cytokine production in T-M3sh EAT mice. **(H, I)** RT-qPCR analysis of **FOXP3, RORγt,** and **IL-17A** expression in spleen **(H)** and thyroid tissues **(I)**. **Data information**: Data are presented as mean ± SD, n=3. Statistical analyses were performed using unpaired two-tailed Student’s t-test for B, C and one-way ANOVA with Tukey’s post hoc test for others. ns, not significant; *P < 0.05; **P < 0.01; ***P < 0.001; ****P < 0.0001.

**Supplementary figure 5.**


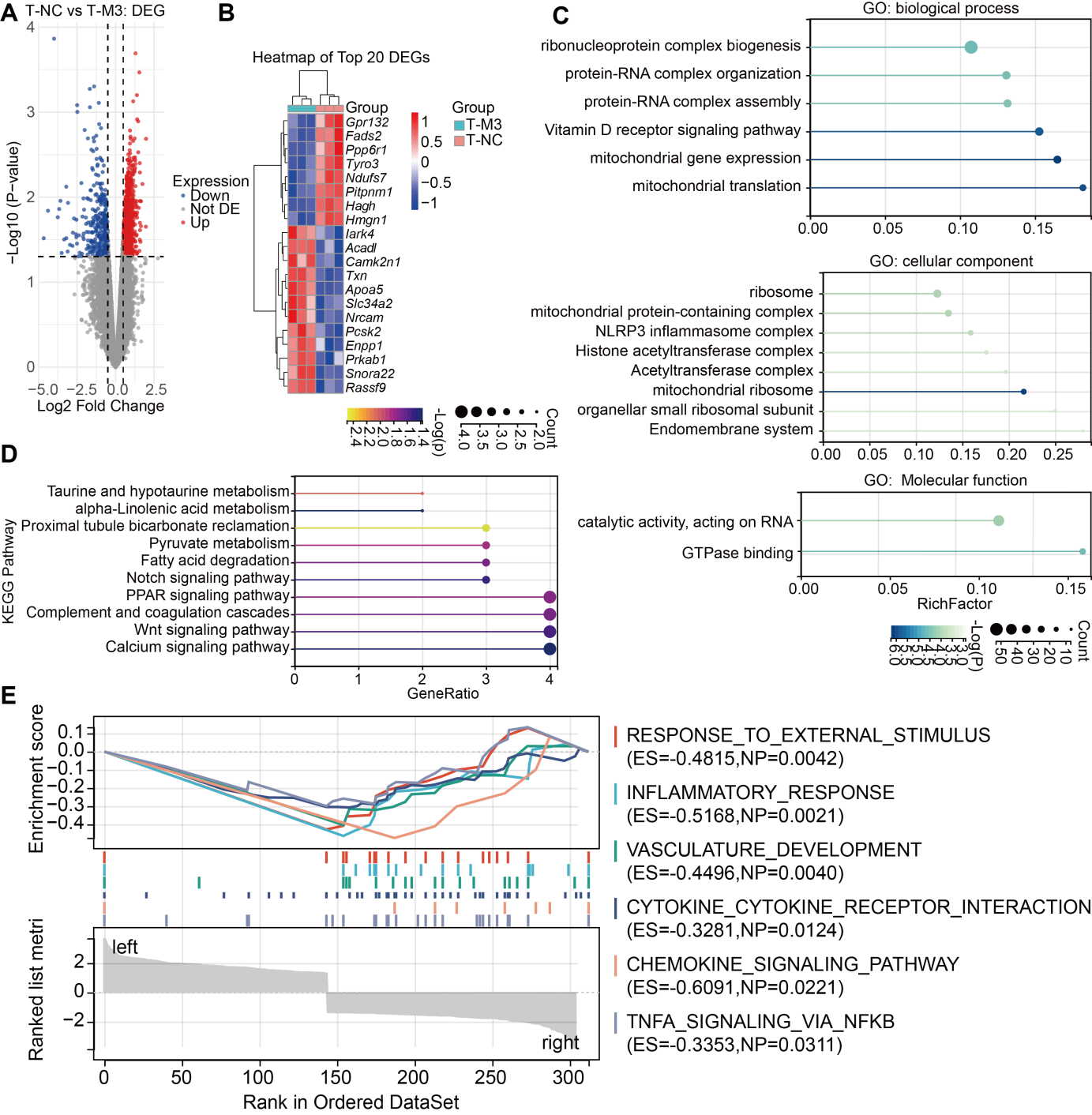
**Figure S5. RNA-seq analysis identifies inflammatory and vascular programs downstream of METTL3 in EAT thyroids. (A, B)** Volcano plot and heatmap plot of RNA-seq analysis of thyroid tissues from EAT mice treated with T-NC or T-M3sh (n = 3). **(C)** GO enrichment analysis of DEGs. **(D)** KEGG pathway analysis revealing enrichment pathways. **(E)** GSEA analysis of DEGs.

**Supplementary figure 6.**


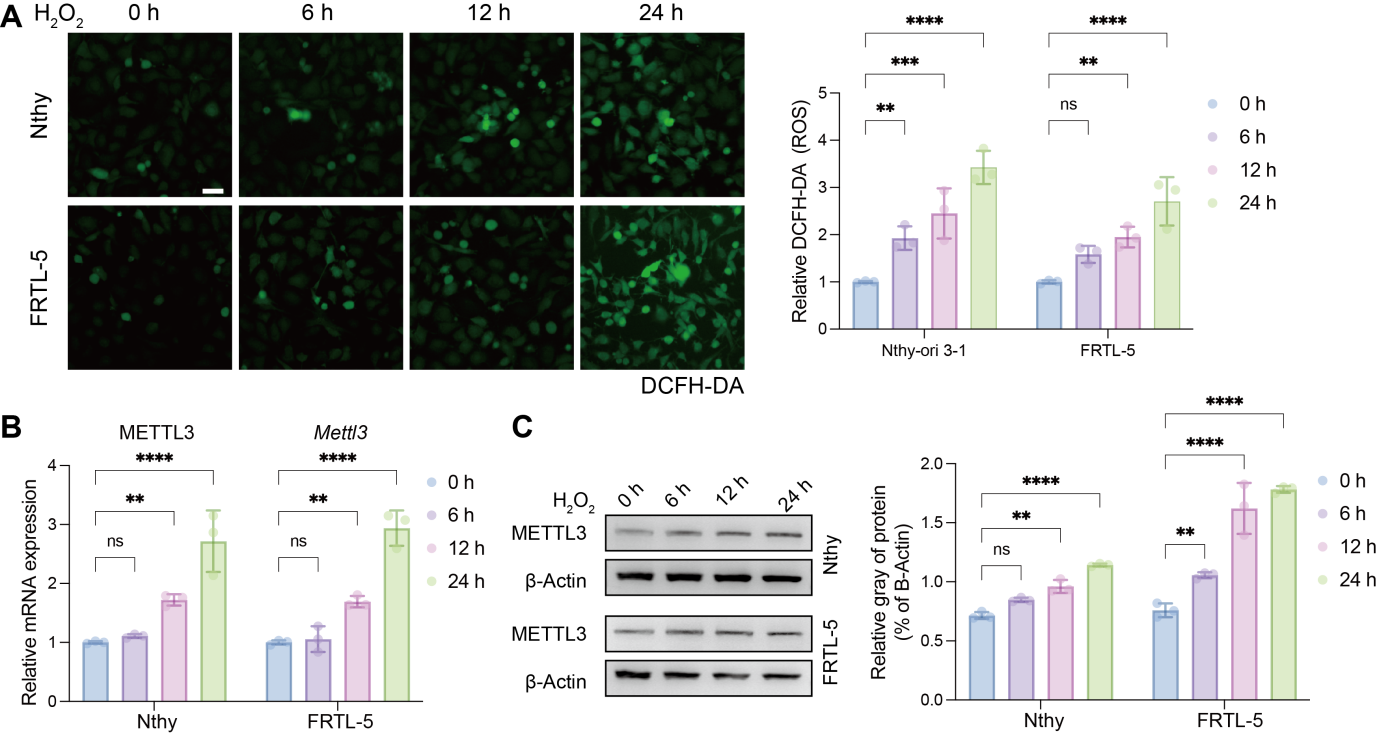


**Figure S6. Oxidative stress induces METTL3 expression in TFCs**. (A) Intracellular ROS levels in TFCs after H2O2 treatment, showing a progressive increase in ROS accumulation over time. (B) RT-qPCR analysis of METTL3 mRNA expression in TFCs treated with H_2_O_2_ for 6, 12, and 24 h, showing a time-dependent increase in METTL3 transcript levels. (C) Western blot analysis of METTL3 protein expression in TFCs after H_2_O_2_ treatment for the indicated time points, showing progressive upregulation of METTL3 protein under oxidative stress. **Data information**: Data are presented as mean ± SD, n=3. Statistical analyses were performed using one-way ANOVA with Tukey’s post hoc test. ns, not significant; *P < 0.05; **P < 0.01; ***P < 0.001; ****P < 0.0001.

**Supplementary figure 7.**


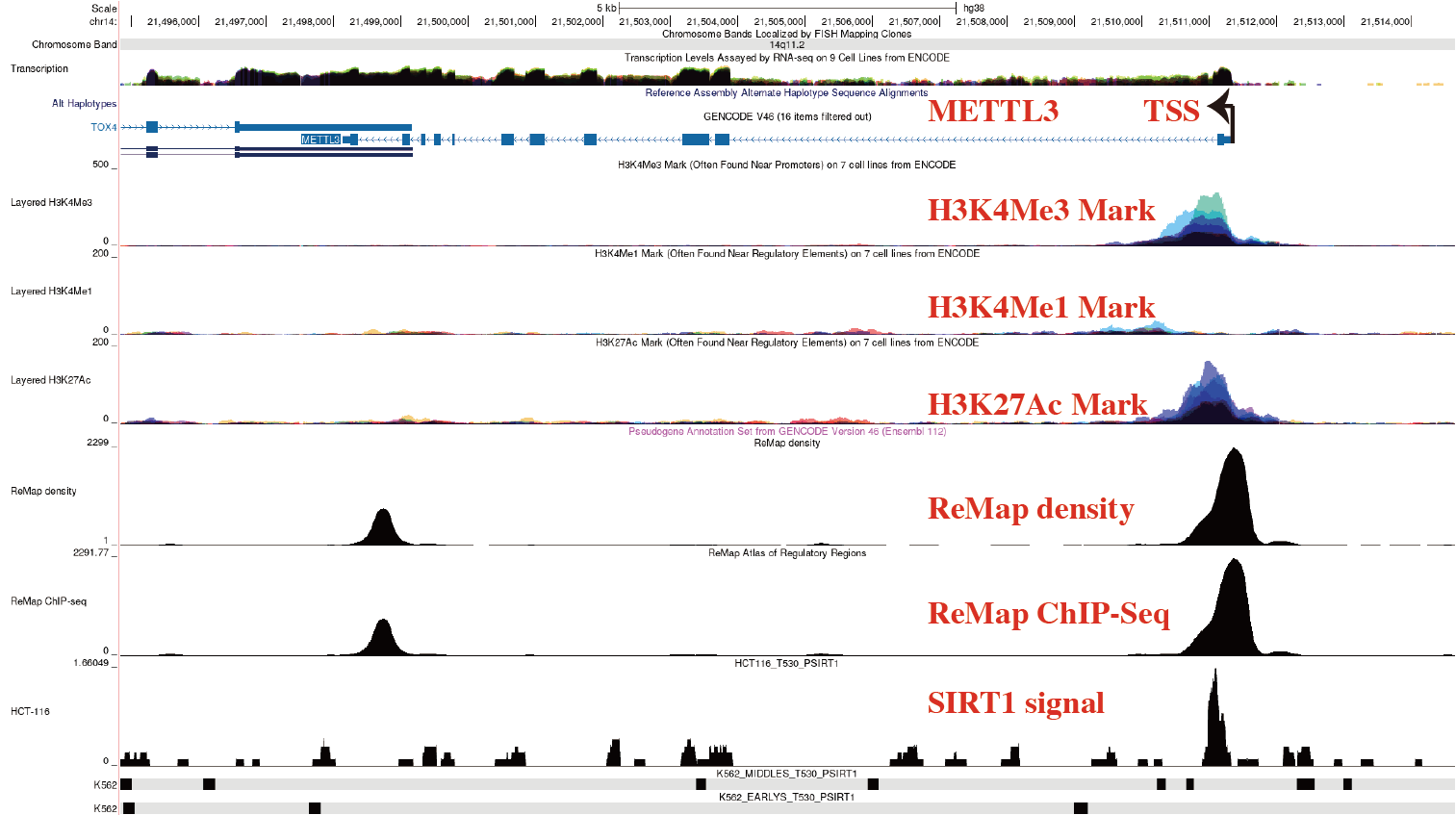


**Figure S7. Enrichment of transcriptionally active histone marks at the METTL3 promoter region.** UCSC Genome Browser-based bioinformatic analysis showing significant enrichment of H3K27ac and H3K4me3 at the METTL3 promoter region. Both modifications are characteristic of transcriptionally active chromatin and support potential epigenetic activation of METTL3 transcription.

**Supplementary figure 8.**


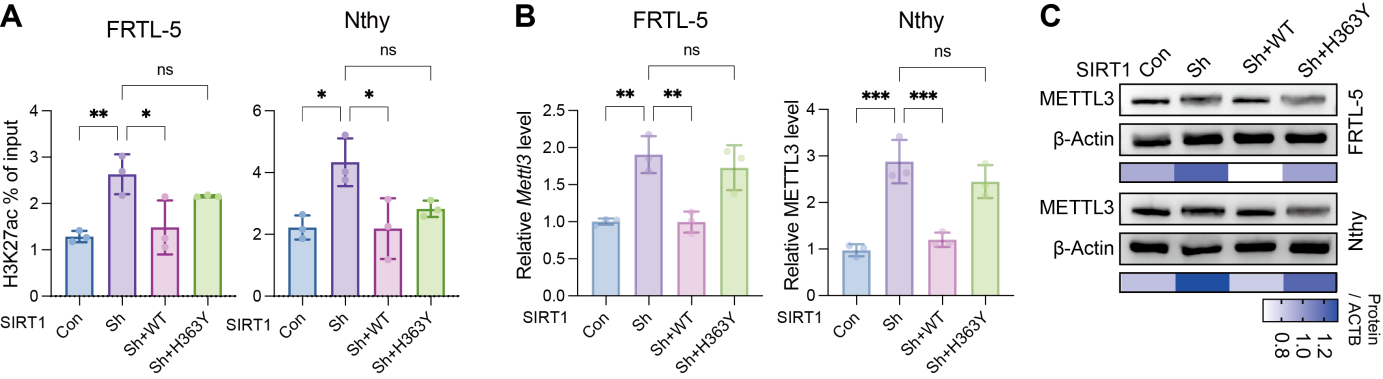


**Figure S8. The inhibitory effect of SIRT1 on METTL3 depends on its deacetylase activity.** (A) ChIP-qPCR analysis of H3K27ac enrichment at the METTL3 promoter in SIRT1-deficient TFCs reconstituted with SIRT1-WT or the deacetylase-defective mutant SIRT1-H363Y. (B-C) RT-qPCR (B) and Western blot (C) analysis of METTL3 expression. Reconstitution with SIRT1-WT, but not SIRT1-H363Y, suppressed METTL3 expression, indicating that SIRT1 regulates METTL3 transcription in a deacetylase activity-dependent manner. **Data information**: Data are presented as mean ± SD, n=3. Statistical analyses were performed using one-way ANOVA with Tukey’s post hoc test. ns, not significant; *P < 0.05; **P < 0.01; ***P < 0.001; ****P < 0.0001.

**Supplementary figure 9.**


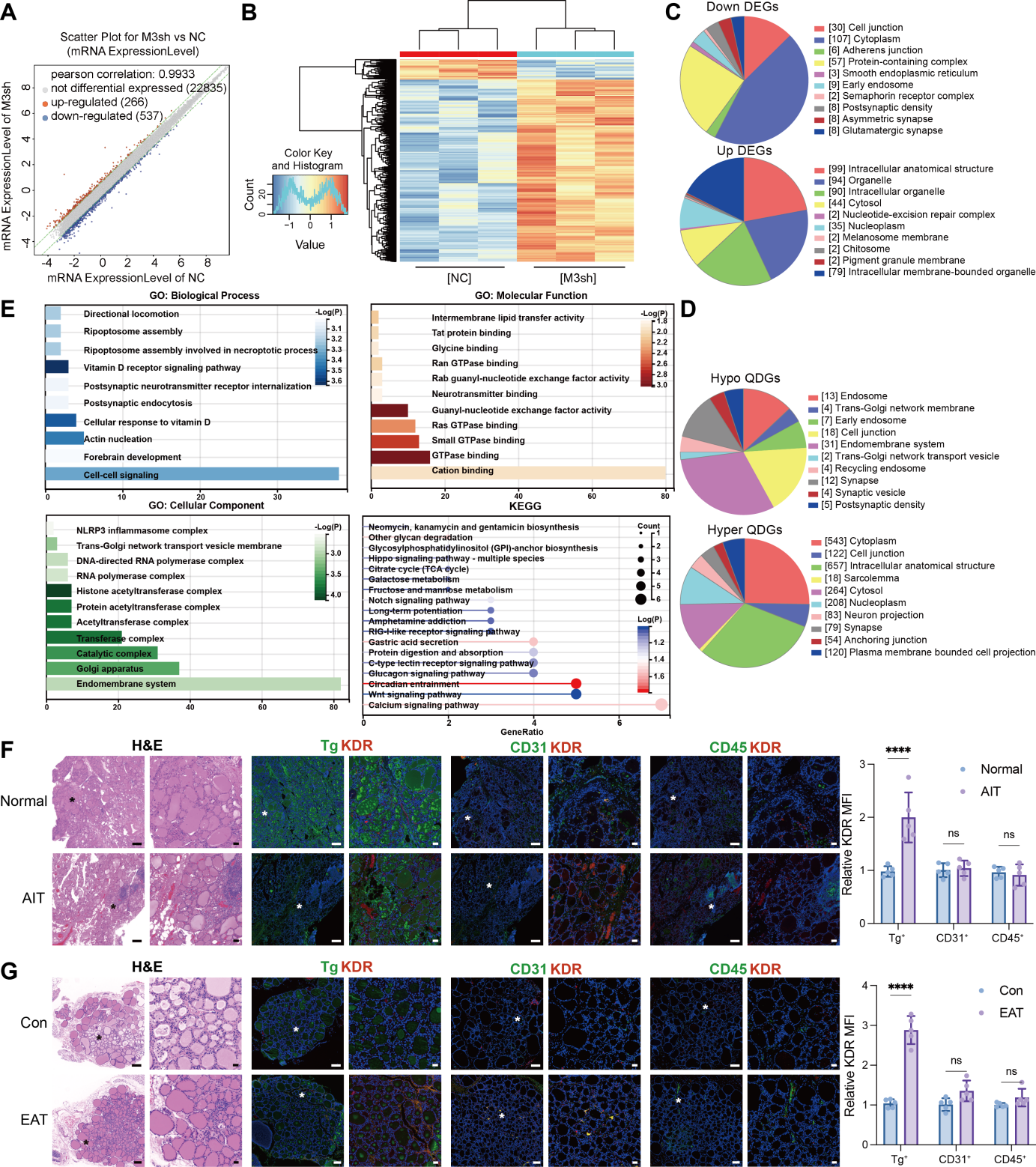


**Figure S9. Integrated m6A epitranscriptomic and transcriptomic profiling identify METTL3-regulated differentially expressed genes (DEGs) and quantitatively differentially methylated genes (QDGs) in autoimmune thyroiditis.** (A) Overview of DEGs in NC and M3sh Nthy-ori 3-1 thyroid cells identified by m6A-mRNA & lncRNA epitranscriptomic microarray. (B) Heatmap of DEGs between NC and M3sh cells. (C) Subcellular localization analysis of DEGs. (D) Subcellular localization analysis of QDGs. (E) GO and KEGG enrichment analyses of overlapping genes between DEGs and QDGs. (F) Representative low- and high-magnification multiplex IF images showing CD31×KDR, CD45×KDR, and Tg×KDR staining in normal and AIT thyroid tissues, together with the corresponding H&E images from the same or closely adjacent tissue regions. Scale bar, 200 μm for low-magnification and 50 μm for high-magnification. (G) Representative low- and high-magnification multiplex IF images showing CD31×KDR, CD45×KDR, and Tg×KDR staining in Control and EAT mice thyroid tissues, together with the corresponding H&E images from the same or closely adjacent tissue regions. Scale bar, 100 μm for low-magnification and 25 μm for high-magnification. In the low-magnification panels, black/white asterisks indicate the areas enlarged in the corresponding high-magnification images. **Data information**: Data are presented as mean ± SD, n=3. Statistical analyses were performed using one-way ANOVA with Tukey’s post hoc test. ns, not significant; *P < 0.05; **P < 0.01; ***P < 0.001; ****P < 0.0001.

**Supplementary figure 10.**


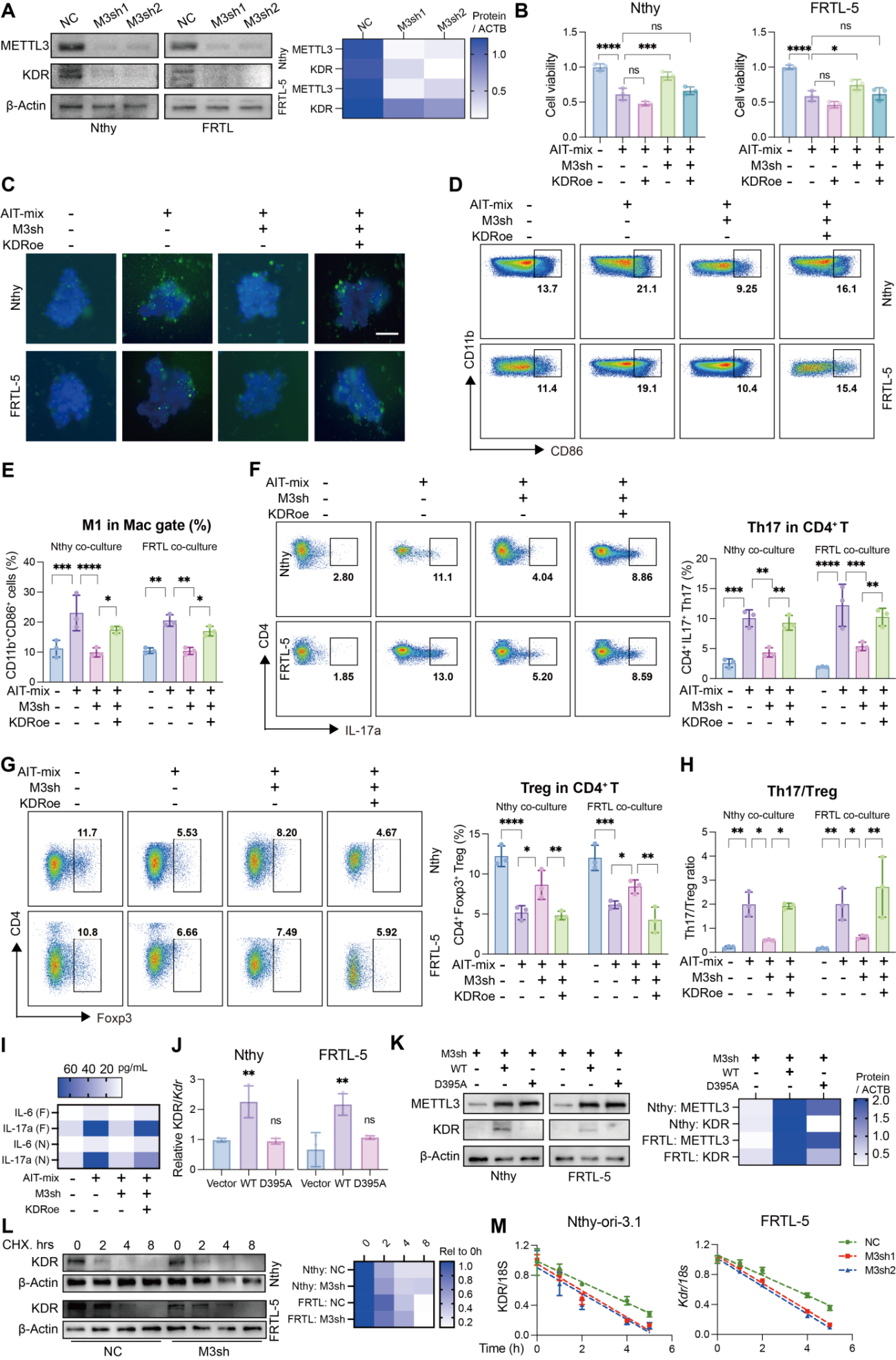


**Figure S10. METTL3 promotes AIT-associated inflammatory phenotypes through m6A-dependent upregulation of KDR in thyroid follicular cells. (A)** Western blot analysis of **KDR** protein expression in TFCs transduced with **NC** or two independent **METTL3** shRNAs (**M3sh1** and **M3sh2**), showing that **KDR** protein levels were decreased after **METTL3** knockdown. **(B)** CCK-8 assay showing that AIT-mix reduced TFCs viability, whereas **METTL3** knockdown alleviated this effect; re-expression of **KDR** partially restored the loss of viability induced by AIT-mix. **(C)** Representative DCFH-DA fluorescence images showing ROS levels in TFCs in the co-culture system under the indicated conditions. AIT-mix-induced ROS accumulation was attenuated by **METTL3** knockdown and partially rescued by **KDR** overexpression. 300 µm. **(D, E)** Representative flow plots and quantitative analysis showing the proportion of CD11b^+^CD86^+^ M1 macrophages in the co-culture system. **(F, G, H)** Representative flow cytometric plots showing the proportion of CD4^+^IL-17A^+^ Th17 cells and CD4^+^Foxp3^+^Treg cell in the co-culture system, corresponding quantitative analysis of Th17, Treg proportion and Th7/Treg ratio. METTL3 knockdown suppressed the effect induced by AIT-mix, which was partially restored by **KDR** overexpression. **(I)** ELISA detection of IL-6, and IL-17A cytokine levels (pg/mL) in co-culture supernatants under the indicated conditions. AIT-mix increased inflammatory cytokine secretion, whereas **METTL3** knockdown reduced these responses; **KDR** overexpression partially restored the inflammatory cytokine profile. **(J, K)** analyses showing that overexpression of wild-type **METTL3** (**METTL3-WT**), but not the catalytically inactive mutant **METTL3-D395A**, increased **KDR/*Kdr*** mRNA and protein levels, indicating that **METTL3** regulates **KDR** expression in an m6A-dependent manner. **(L)** Cycloheximide (CHX) chase assay showing that inhibition of translation did not support a major effect of **METTL3** on **KDR** protein stability. **(M)** Actinomycin D assay followed by RT-qPCR analysis showing accelerated **KDR** mRNA decay in **M3sh1**- and **M3sh2**-transduced TFCs compared with **NC** cells. Scatter plots were fitted with linear regression lines to illustrate the rate of **KDR/*Kdr*** mRNA degradation, indicating that **METTL3** maintains **KDR/*Kdr*** expression by stabilizing its mRNA. **Data information**: Data are presented as mean ± SD or as appropriate. Statistical significance was determined using one-way ANOVA followed by Tukey’s multiple-comparisons test for comparisons among multiple groups, and two-way ANOVA for time-course analyses where applicable. ns, not significant; *P < 0.05; **P < 0.01; ***P < 0.001; ****P < 0.0001.

**Supplementary figure 11.**


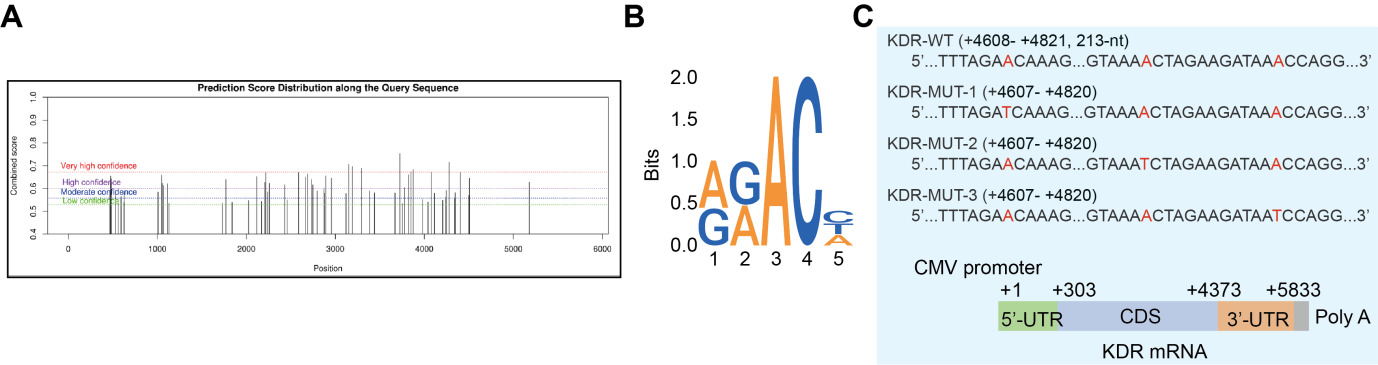


**Figure S11. Identification of the candidate METTL3-dependent m6A-modified region on KDR mRNA.** (A) SRAMP-based prediction of potential m6A sites on the KDR transcript. (B) Schematic illustration of the predicted m6A motifs on KDR mRNA. (C) Schematic of three mutant constructs generated to target the three putative m6A sites within the primer 3-enriched region.

**Supplementary figure 12.**


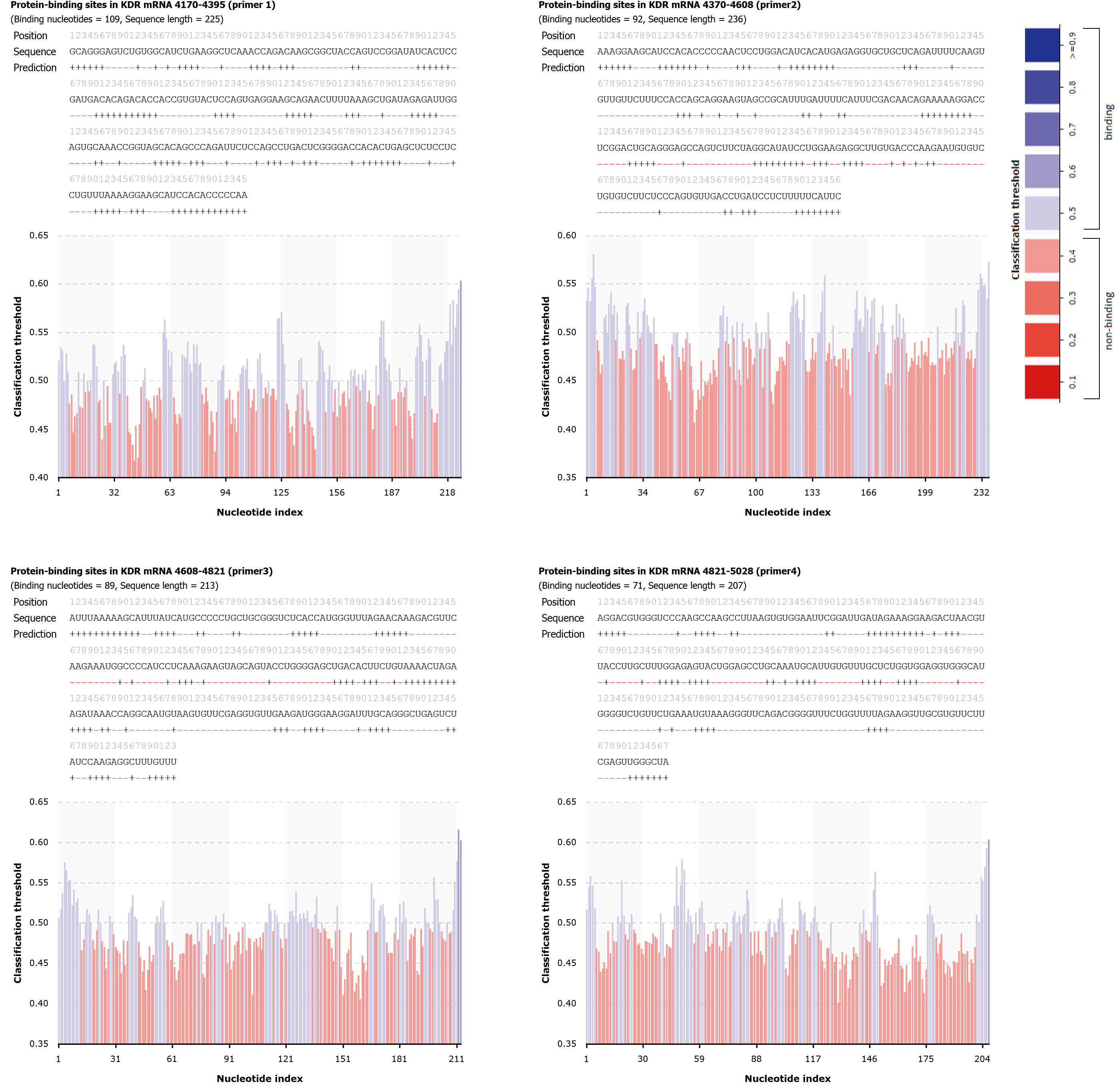
**Figure S12. Prediction of YTHDC2 as a candidate m6A reader for KDR mRNA.** Bioinformatic analysis using the PRIdictor platform predicted a high-affinity interaction between **YTHDC2** and **KDR** mRNA. The predicted **YTHDC2**-binding region overlapped with the m6A-modified segment identified on the **KDR** transcript, suggesting that **YTHDC2** may function as a candidate m6A reader mediating **KDR** RNA regulation.

**Supplementary figure 13.**


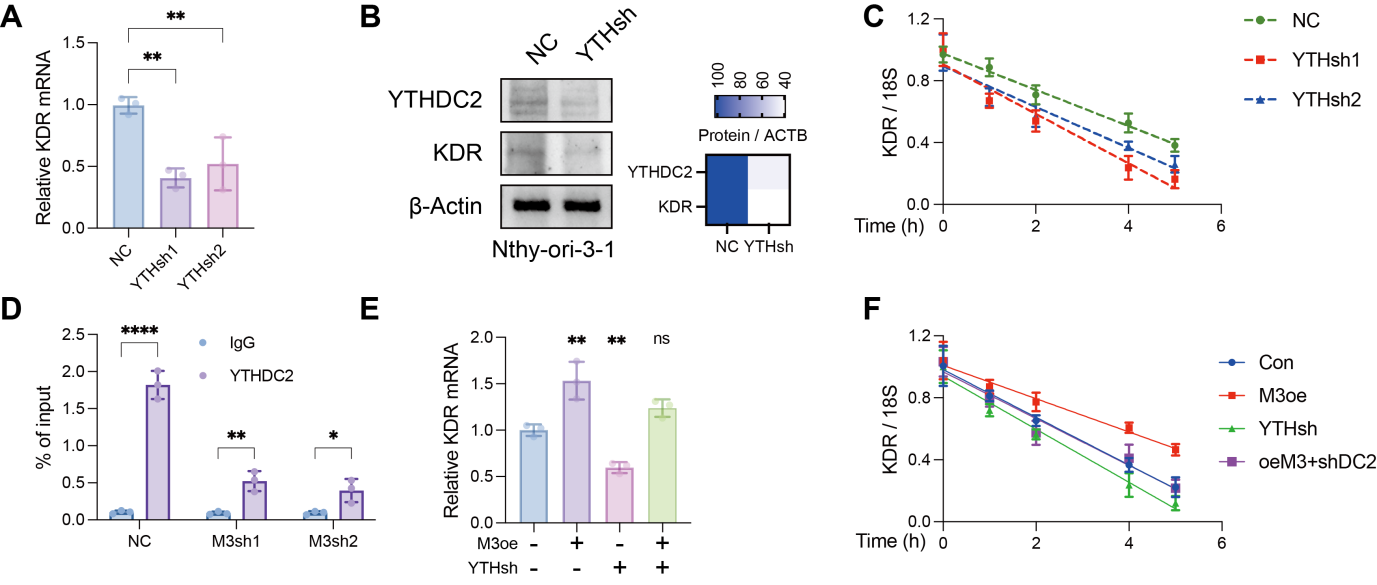


**Figure S13. YTHDC2 binds METTL3-dependent m6A-modified KDR mRNA and promotes its stability in Nthy-ori 3-1**. **(A, B)** RT-qPCR **(A)** and western blot **(B)** analyses showing that knockdown of **YTHDC2** using two independent shRNAs significantly reduced **KDR** mRNA and protein expression. **(C)** Actinomycin D chase assay followed by RT-qPCR analysis showing that **YTHDC2** knockdown accelerated **KDR** mRNA decay, data are presented as Mean ± 95% CI. **(D)** RIP assay showing that **YTHDC2** was enriched at the m6A-modified region of the **KDR** transcript, and this enrichment was reduced upon **METTL3** knockdown. **(E)** RT-qPCR analysis showing that **YTHDC2** knockdown abolished the **METTL3**-mediated upregulation of **KDR** expression. **(F)** mRNA stability assay showing that **YTHDC2** knockdown abrogated the **METTL3**-mediated stabilization of **KDR** mRNA, data are presented as Mean ± 95% CI. **Data information**: Data are presented as mean ± SD or as appropriate, n=3. Statistical significance was determined using one-way ANOVA followed by Tukey’s multiple-comparisons test for comparisons among multiple groups, and two-way ANOVA for time-course analyses where applicable. ns, not significant; *P < 0.05; **P < 0.01; ***P < 0.001; ****P < 0.0001.

**Supplementary figure 14.**


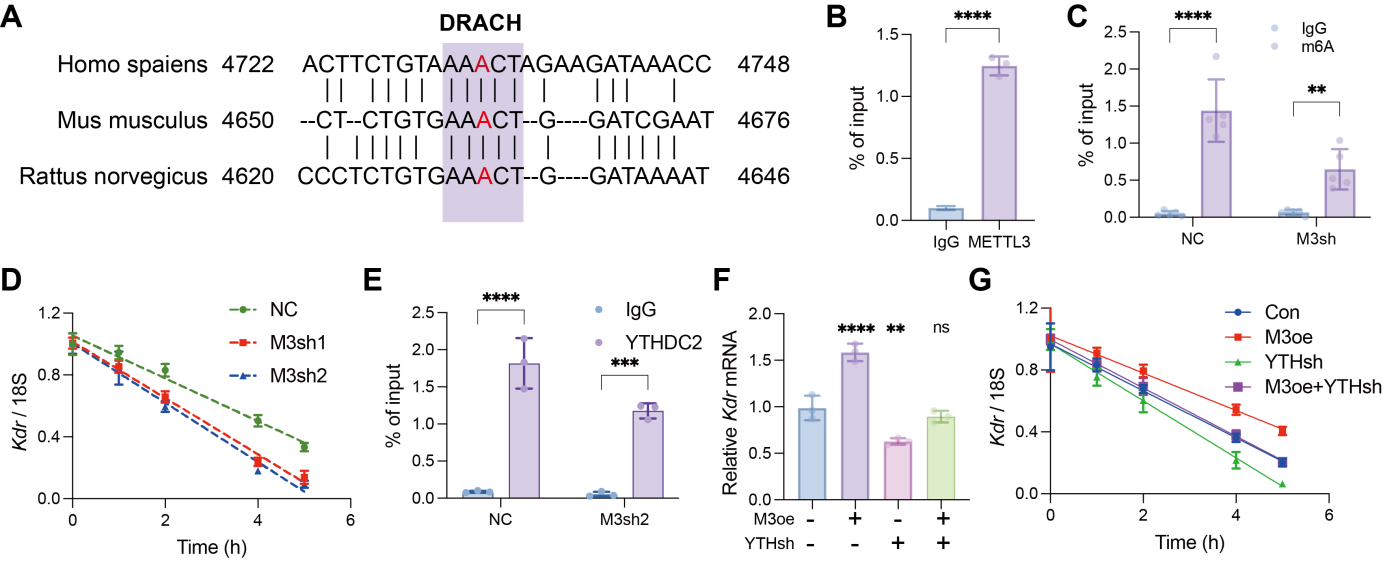
**Figure S14. The conserved m6A site on *Kdr*** **mRNA mediates METTL3/YTHDC2-dependent regulation across species.** **(A)** Sequence alignment showing conservation of the DRACH motif corresponding to the KDR/***Kdr*** +4731 m6A site in human, rat, and mouse transcripts. **(B)** RIP assay showing **METTL3** binding to ***Kdr*** mRNA in rat FRTL-5 cells. **(C)** MeRIP-qPCR showing reduced m6A modification of ***Kdr*** mRNA after **METTL3** knockdown (M3sh). **(D)** Actinomycin D assay showing accelerated ***Kdr*** mRNA decay in **M3sh1**- and **M3sh2**-cells compared with NC, data are presented as Mean ± 95% CI. **(E)** RIP assay showing that **YTHDC2** binds ***Kdr*** mRNA and that this enrichment is reduced in M3sh compared with NC. **(F)** RT-qPCR showing that **YTHDC2** knockdown (YTHsh) reversed the increase in ***Kdr*** mRNA induced by **METTL3** overexpression (M3oe). **(G)** mRNA stability assay showing that **YTHsh** abolished the stabilizing effect of **M3oe** on ***Kdr*** mRNA, data are presented as Mean ± 95% CI. **Data information**: Data are presented as mean ± SD or as appropriate, n=3. Statistical significance was determined using one-way ANOVA followed by Tukey’s multiple-comparisons test for comparisons among multiple groups, and two-way ANOVA for time-course analyses where applicable. ns, not significant; *P < 0.05; **P < 0.01; ***P < 0.001; ****P < 0.0001.

**Supplementary figure 15.**


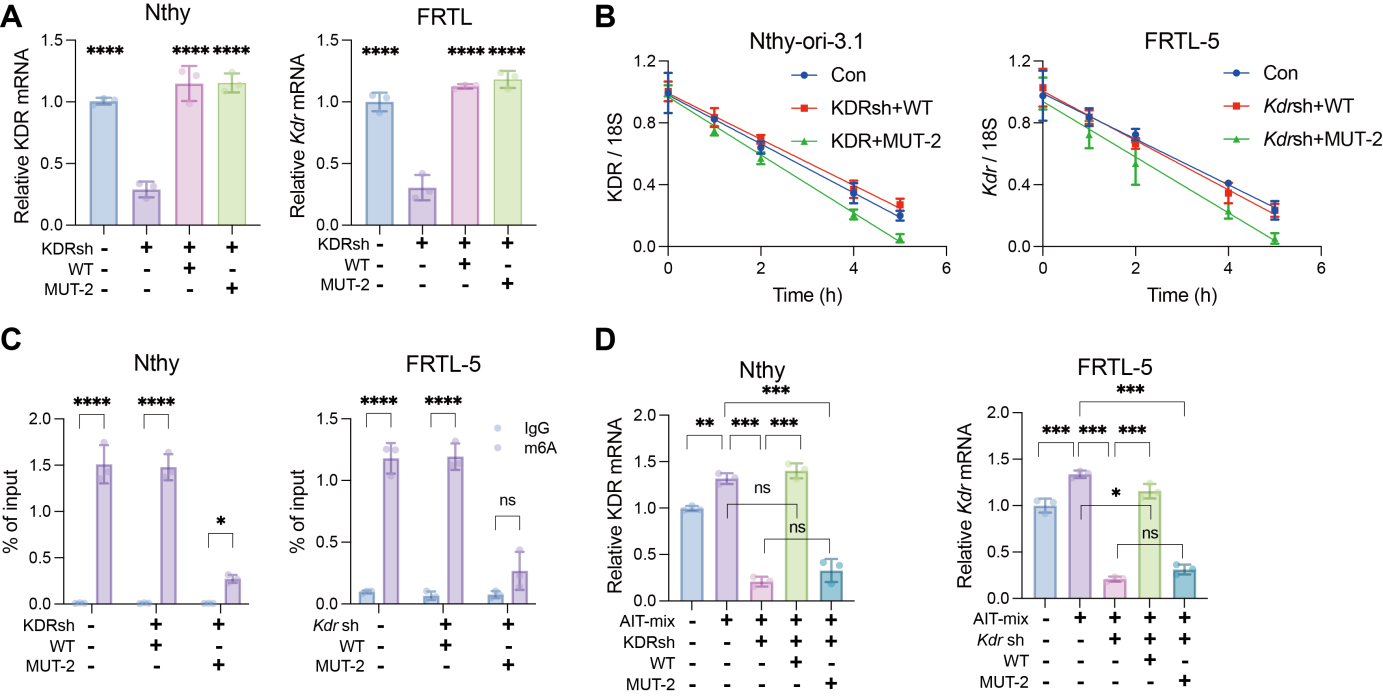


**Figure S15. The +4731 m6A site is required for METTL3–YTHDC2-mediated regulation of KDR expression. (A)** RT-qPCR validation of exogenous transcript expression in **KDR**-silenced TFCs, followed by reconstitution with wild-type (**WT**) or the **+4731 site-deficient mutant** (MUT-2). **(B)** Actinomycin D chase assay showing that **METTL3** knockdown shortened the half-life of **WT** mRNA, whereas no obvious effect was observed on MUT-2 transcript stability, data are presented as Mean ± 95% CI. **(C)** MeRIP-qPCR analysis showing that **METTL3** knockdown reduced m6A modification of **WT**, but had little or no effect on the **MUT-2** transcript. **(D)** RT-qPCR analysis showing that AIT-mix treatment upregulated **WT** expression, whereas MUT-2 showed no significant response. **Data information**: Data are presented as mean ± SD or as appropriate, n=3. Statistical significance was determined using one-way ANOVA followed by Tukey’s multiple-comparisons test or two-way ANOVA for time-course analyses, as appropriate. ns, not significant; *P < 0.05; **P < 0.01; ***P < 0.001; ****P < 0.0001.

**Supplementary figure 16.**


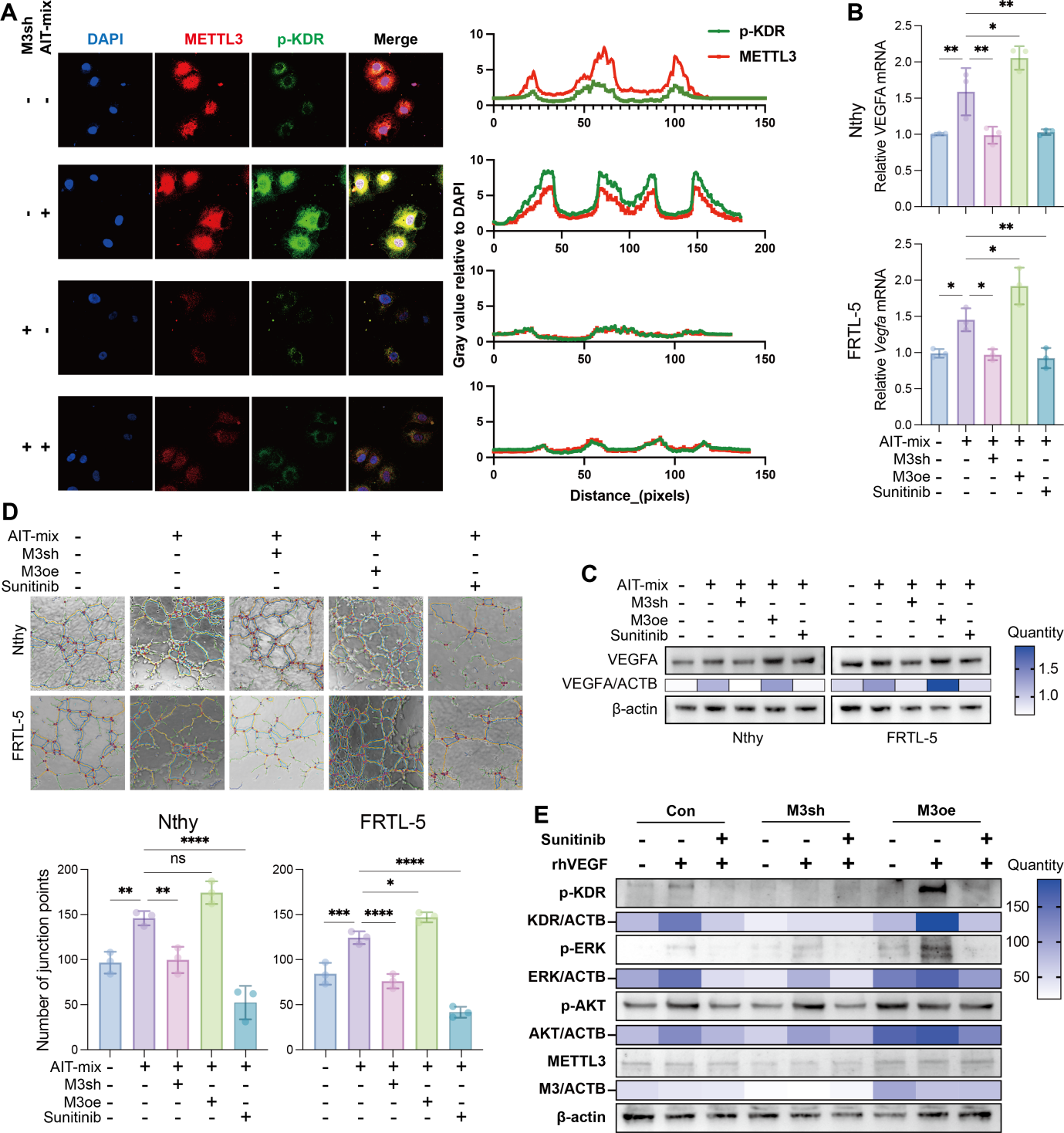


**Figure S16. METTL3 potentiates VEGFA–KDR signaling in TFCs.** **(A)** IF of **METTL3**, **p-KDR**, and DAPI in TFCs under the indicated conditions. AIT-mix increased **KDR** phosphorylation, which was attenuated by **METTL3** knockdown. **(B, C)** RT-qPCR and western blot analyses showing AIT-mix-induced **VEGFA** upregulation in TFCs, which was enhanced by M3oe, and reversed by M3sh or sunitinib pre-treatment. **(D)** HUVEC tube formation assay using CM from TFCs under the indicated treatments, showing that AIT-mix enhanced pro-angiogenic activity in a **METTL3**-dependent manner and that **sunitinib** suppressed this effect. **(E)** Western blot analysis showing that **sunitinib** blocked VEGFA-induced **KDR** signaling in **NC**, **M3sh**, and **M3oe** TFCs. Data information: Data are presented as mean ± SD, n=3. Statistical analyses were performed using one-way ANOVA with Tukey’s post hoc test or two-way ANOVA where appropriate. ns, not significant; *P < 0.05; **P < 0.01; ***P < 0.001; ****P < 0.0001.

**Supplementary figure 17.**


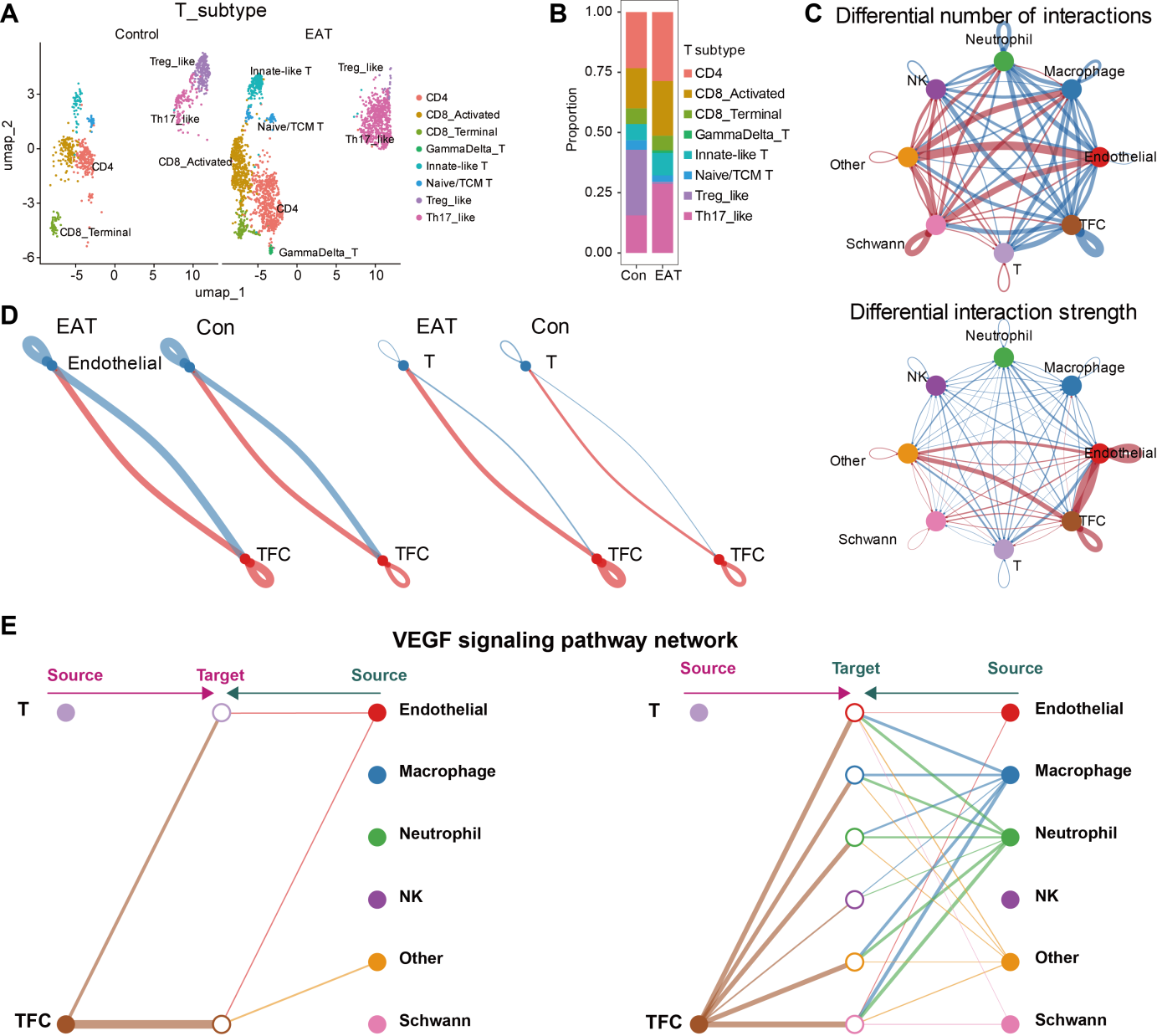
**Figure S17. Single-cell analysis reveals T cell remodeling and enhanced VEGF-centered intercellular communication driven by TFCs in EAT**. **(A, B)** Subclustering analysis of T cells in thyroid tissues based on scRNA-seq data. UMAP visualization and corresponding cell composition analysis showed increased proportions of **Th17 cells, CD8**^+^ **T cells,** and **activated CD4**^+^ **T cells**, together with a reduced proportion of **Treg** cells in EAT compared with control mice. **(C)** Circle plot of the CellChat analysis of scRNA-seq data showing that, compared with controls, **interaction strength** involving TFCs was markedly increased in EAT, whereas the overall **interaction number** showed no obvious increase. **(D)** Cell–cell communication analysis showing that TFCs, as signaling senders, exhibited enhanced outgoing interactions toward **endothelial cells, T cells,** and **TFCs themselves** in EAT. **(E)** Hierarchical CellChat network analysis of the **VEGF signaling pathway** showing that VEGF-mediated communication formed a broad intercellular signaling network in the EAT microenvironment. Incoming VEGF signaling toward TFCs was relatively limited and mainly originated from **endothelial cells** and **TFCs themselves**, whereas outgoing VEGF signaling from TFCs was substantially broader, targeting multiple cell populations. Interactions between TFCs and **endothelial cells, T cells,** and **neutrophils** were particularly prominent.

**Supplementary figure 18.**


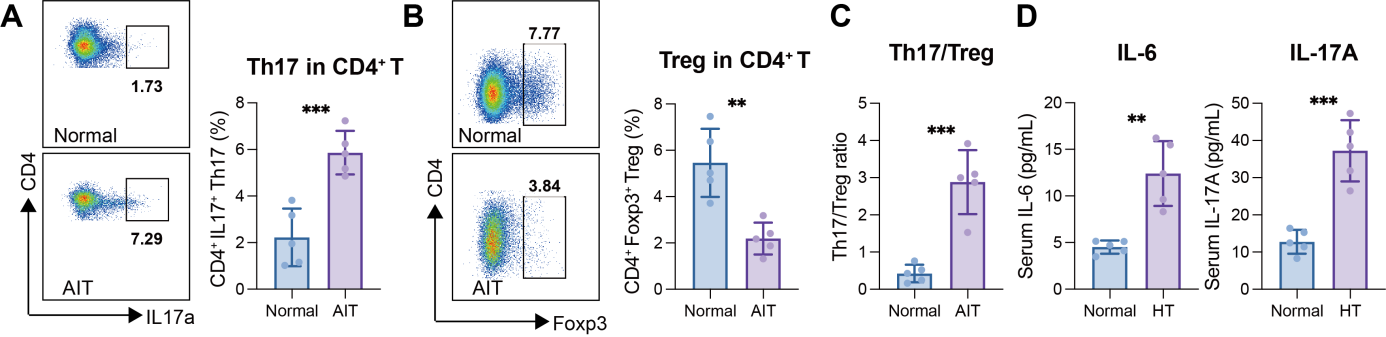


**Figure S18. TFC-specific METTL3 knockdown alleviates Th17/Treg imbalance in AIT and EAT.** **(A, B)** Representative flow cytometry plots and quantification of **CD4**^+^**IL-17A**^+^ **Th17** cells **(A)** and **CD4**^+^**FOXP3**^+^ **Treg** cells **(B)** in peripheral blood mononuclear cells (PBMCs) from AIT patients and normal controls. **(C)** Quantification of the **Th17/Treg ratio** in PBMCs showing an increased ratio in AIT patients compared with normal controls. **(D)** ELISA analysis of serum **IL-17A** and **IL-6** levels in AIT patients and normal controls. **Data information**: Data are presented as mean ± SD. Statistical significance was determined using unpaired two-tailed Student’s t-test for two-group comparisons. ns, not significant; *P < 0.05; **P < 0.01; ***P < 0.001; ****P < 0.0001.

**Supplementary figure 19.**


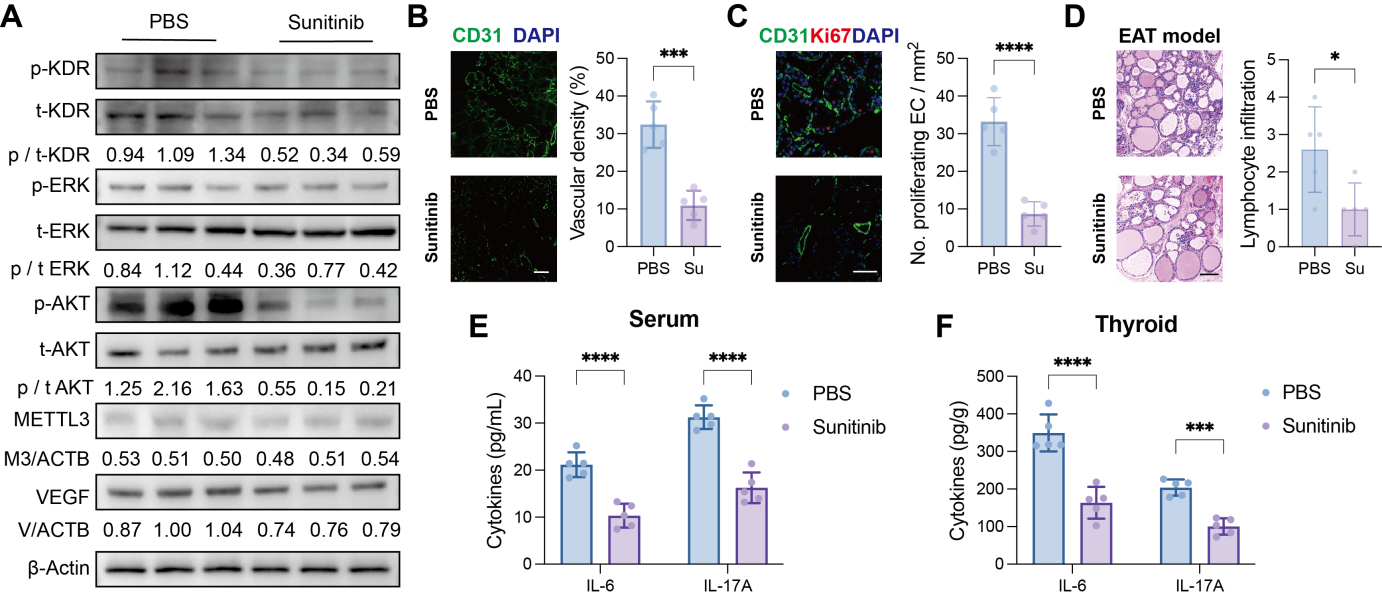


**Figure S19. Pharmacological inhibition of KDR by sunitinib attenuates vascular, inflammatory, and oxidative injury in EAT mice. (A)** Western blot analysis of thyroid tissues from EAT mice treated with **PBS** or **sunitinib**, showing that sunitinib reduced phosphorylation of **KDR** and its downstream signaling pathways. **(B)** Representative IF of **CD31** (green) and DAPI (blue) in thyroid tissues showing that sunitinib treatment normalized vascular density in EAT thyroids. Scale bar, 100µm. **(C)** Analysis of endothelial cell viability in thyroid tissues from PBS or sunitinib treated EAT mice, Ki67 (red), CD31 (green) and DAPI (blue). Scale bar, 50 µm. **(D)** Representative H&E staining and corresponding inflammatory score quantification of thyroid tissues in EAT mice treated with PBS or sunitinib. Scale bar, 100 µm. **(E, F)** ELISA analysis showing that sunitinib treatment reduced **VEGFA**, **IL-6**, and **IL-17A** levels in serum **(E)** and thyroid tissues **(F)** from EAT mice treated with PBS or sunitinib. **Data information**: Data are presented as mean ± SD, in A-D n = 3, in E-F n = 5. Statistical significance was determined using unpaired two-tailed Student’s t-test for two-group comparisons. ns, not significant; *P < 0.05; **P < 0.01; ***P < 0.001; ****P < 0.0001.

**Supplementary figure 20.**


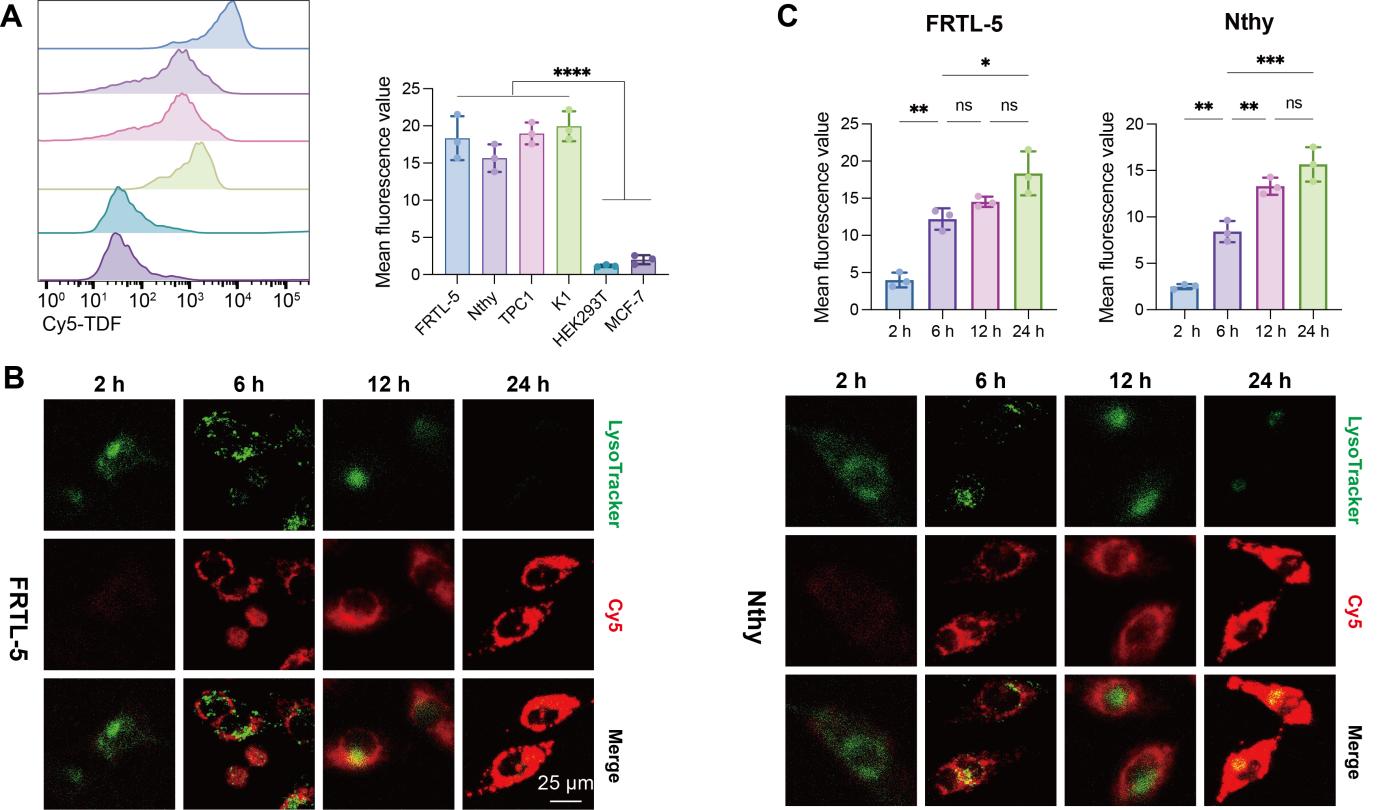


**Figure S20. Thyroglobulin-targeted DNA framework is selectively internalized by Tg-expressing thyroid cells and escapes from lysosomes.** **(A)** Representative flow cytometry image showing cellular uptake of **Cy5-labeled TDF** in **Tg-high thyroid follicular cells** (**FRTL-5** and **Nthy-ori 3-1**) and **thyroid cancer cells** (**TPC1** and **K1**), but minimal uptake in **Tg-negative** cells (**HEK293T** and **MCF7**). **(B-C)** CLSM images and quantitative analysis showing limited co-localization of internalized **TDF (Cy5-labeled, red)** with lysosomes (**LysoTracker, green)**, indicating efficient endosomal/lysosomal escape after cellular uptake. Scale bar, 25 µm. **Data information**: Data are presented as mean ± SD, n = 3. Statistical significance was determined using one-way ANOVA followed by Tukey’s multiple-comparisons test for comparisons among multiple groups. ns, not significant; *P < 0.05; **P < 0.01; ***P < 0.001; ****P < 0.0001.

**Supplementary figure 21.**


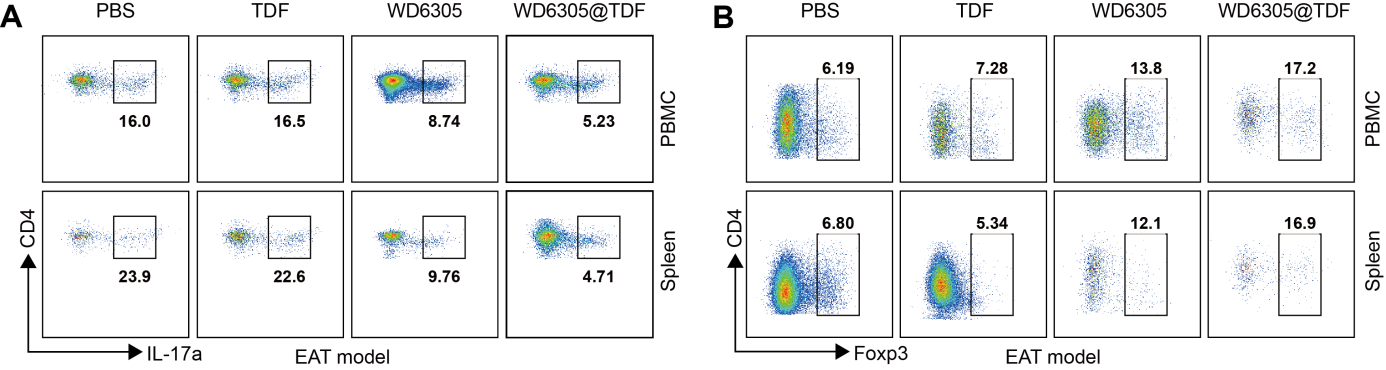


**Figure S21. Representative Flow cytometric images showing that WD6305@TDF restored immune balance in both thyroid tissues and peripheral blood, as reflected by decreased Th17 cell proportions (A), increased Treg cell proportions (B).**

**Reference**

[1] H. T. Celik, S. Abusoglu, S. F. Burnik, et al. Increased serum interleukin-33 levels in patients with Graves' disease [J]. Endocr Regul, 2013, 47(2): 57-64.doi: 10.4149/endo_2013_02_57.

[2] C. He, Y. Li, L. Gan, et al. Notch signaling regulates Th17 cells differentiation through PI3K/AKT/mTORC1 pathway and involves in the thyroid injury of autoimmune thyroiditis [J]. J Endocrinol Invest, 2024, 47(8): 1971-1986.doi: 10.1007/s40618-023-02293-z.

[3] P. Zaccone, Z. Fehérvári, A. Cooke. Tumour necrosis factor-alpha is a fundamental cytokine in autoimmune thyroid disease induced by thyroglobulin and lipopolysaccharide in interleukin-12 p40 deficient C57BL/6 mice [J]. Immunology, 2003, 108(1): 50-54.doi: 10.1046/j.1365-2567.2003.01547.x.

[4] N. Zhao, Z. Wang, X. Cui, et al. In Vivo Inhibition of MicroRNA-326 in a NOD.H-2(h4) Mouse Model of Autoimmune Thyroiditis [J]. Front Immunol, 2021, 12: 620916.doi: 10.3389/fimmu.2021.620916.

[5] E. J. Kim, J. H. Kho, M. R. Kang, et al. Active regulator of SIRT1 cooperates with SIRT1 and facilitates suppression of p53 activity [J]. Mol Cell, 2007, 28(2): 277-290.doi: 10.1016/j.molcel.2007.08.030.

[6] L. Cheng, G. Wu, W. Yao, et al. Nuclear-localized SIRT1 inhibits apoptosis via deacetylating p53 [J]. Int J Biochem Cell Biol, 2025, 187: 106841.doi: 10.1016/j.biocel.2025.106841.
